# Supplementary figures and images for: Early steps of protein disaggregation by Hsp70 chaperone and class B J-domain proteins are shaped by Hsp110 (part 2 of 3)
Source: eLife. 2024 Oct 15;13:RP94795. doi: 10.7554/eLife.94795 (PMC11479587; doi:10.7554/eLife.94795)

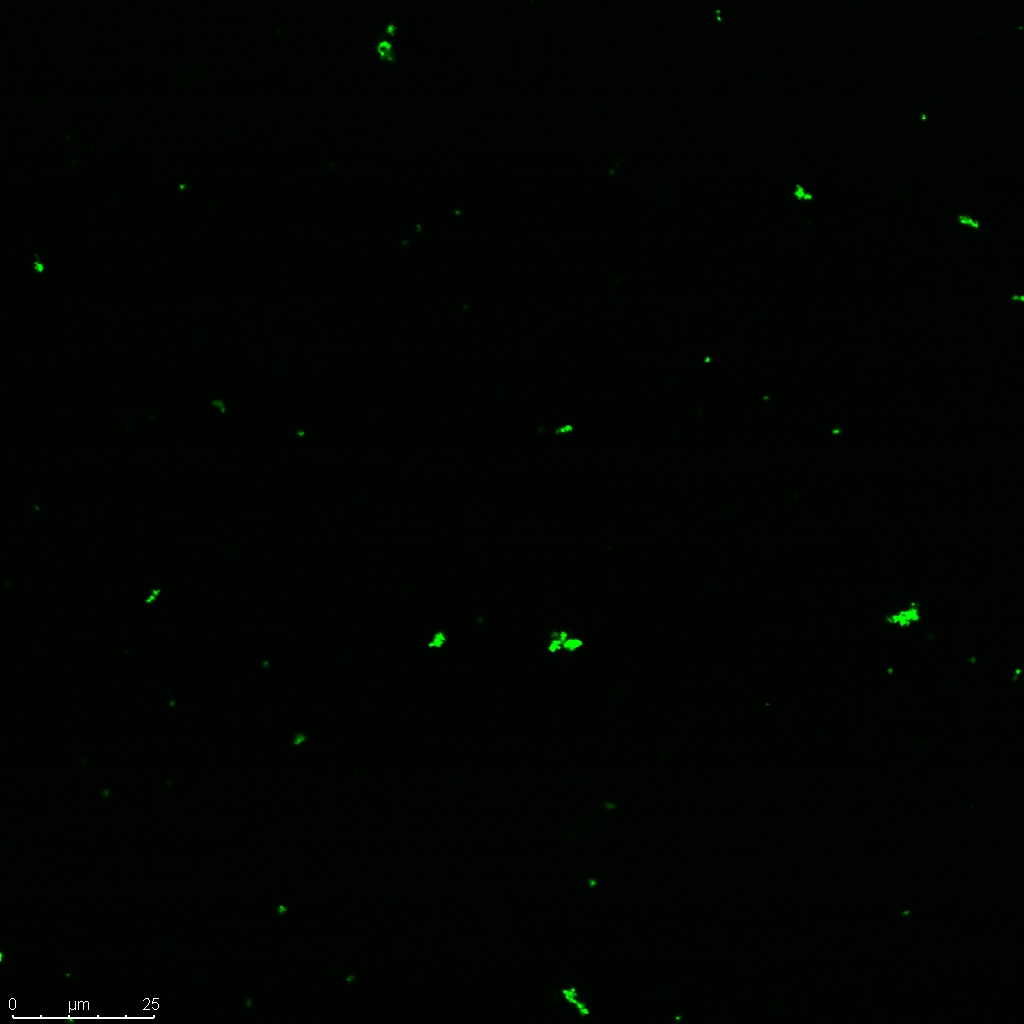

Supplement: Figure 2—source data 3. [file elife-94795-fig2-data3.zip › Figure 2B/replicate II/Figure 2B panel 6 Luc agg SSS replicate 2 photo 1.jpg]

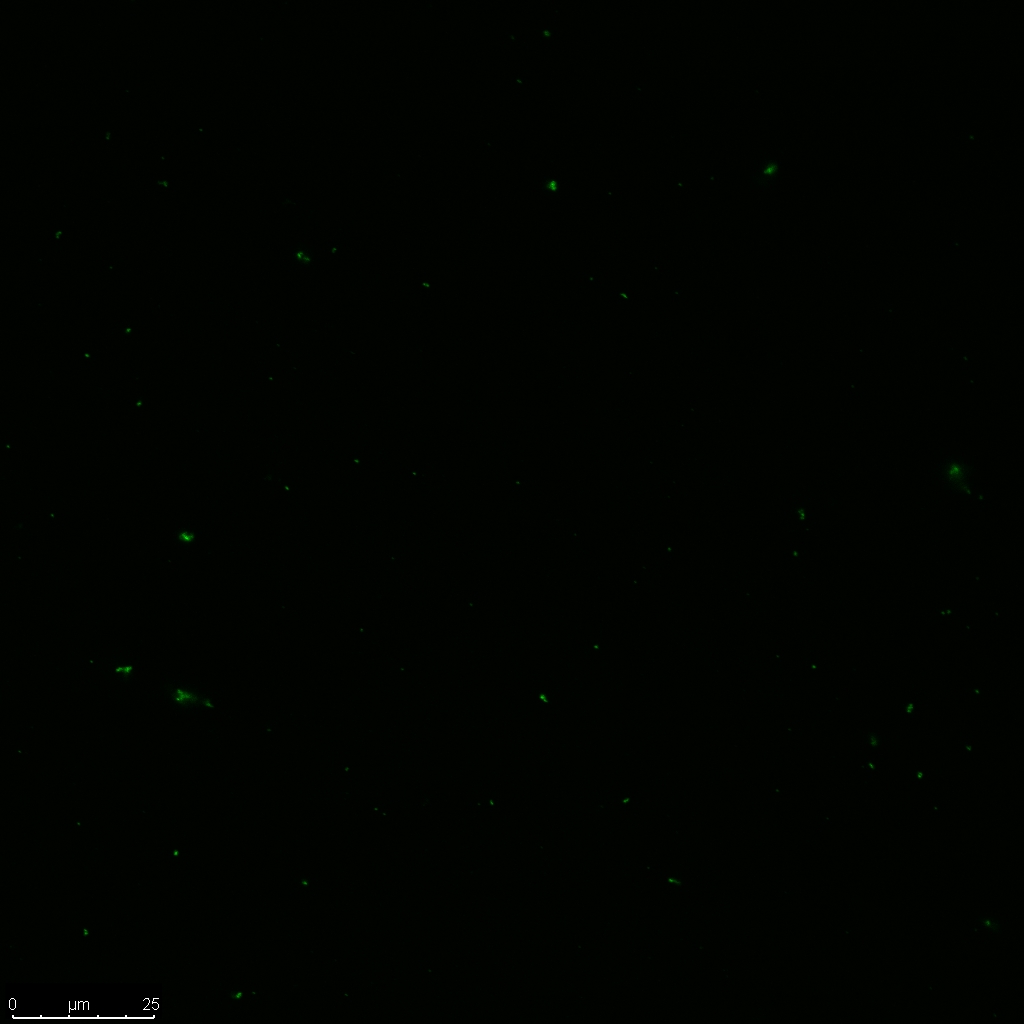

Supplement: Figure 2—source data 3. [file elife-94795-fig2-data3.zip › Figure 2B/replicate II/Figure 2B panel 6 Luc agg SSS replicate 2 photo 2.jpg]

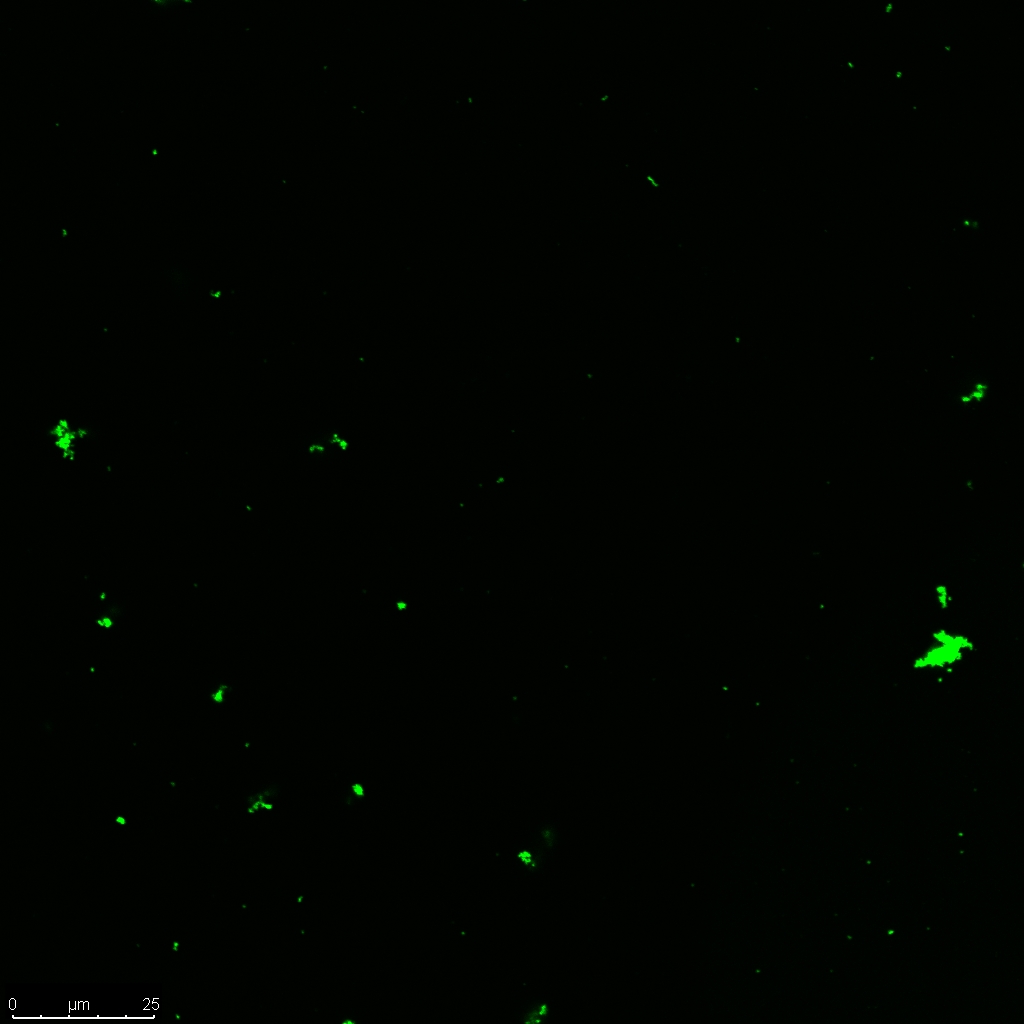

Supplement: Figure 2—source data 3. [file elife-94795-fig2-data3.zip › Figure 2B/replicate II/Figure 2B panel 6 Luc agg SSS replicate 2 photo 3.jpg]

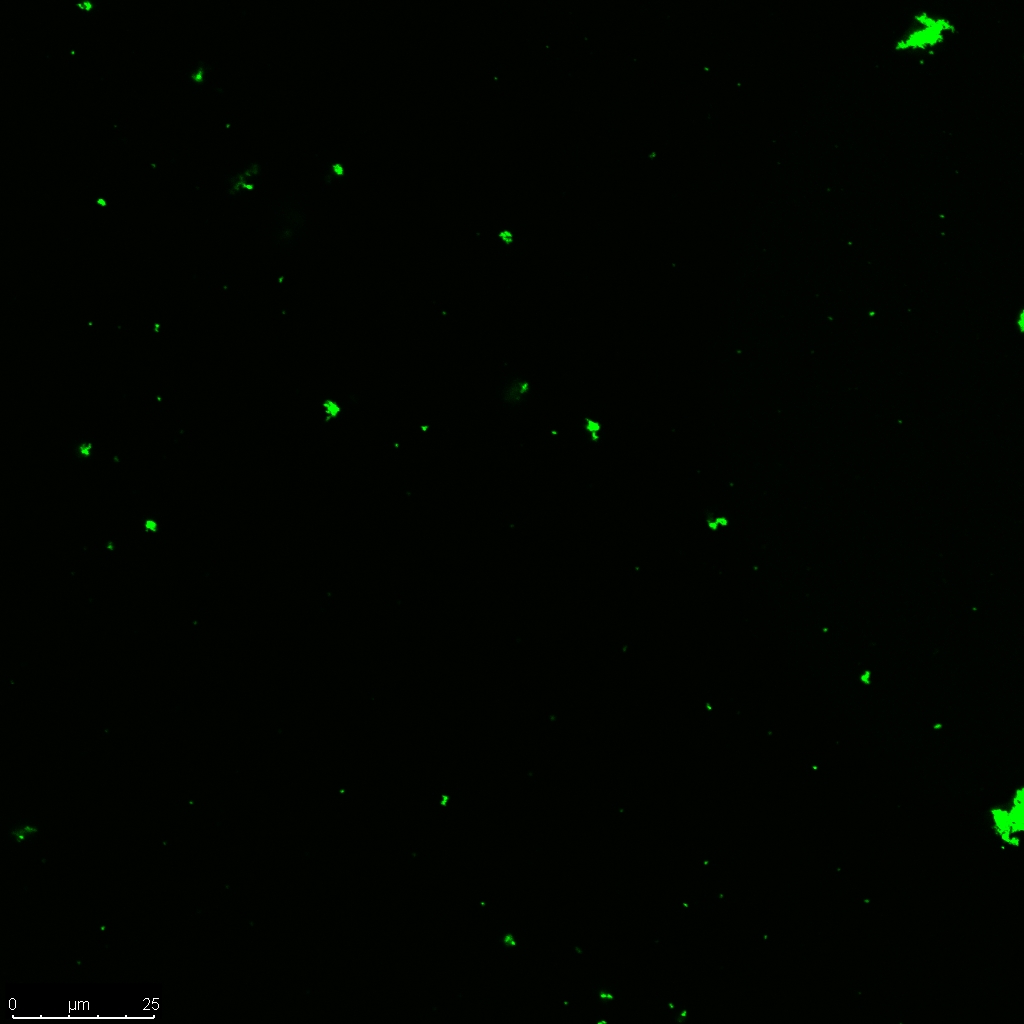

Supplement: Figure 2—source data 3. [file elife-94795-fig2-data3.zip › Figure 2B/replicate II/Figure 2B panel 6 Luc agg SSS replicate 2 photo 4.jpg]

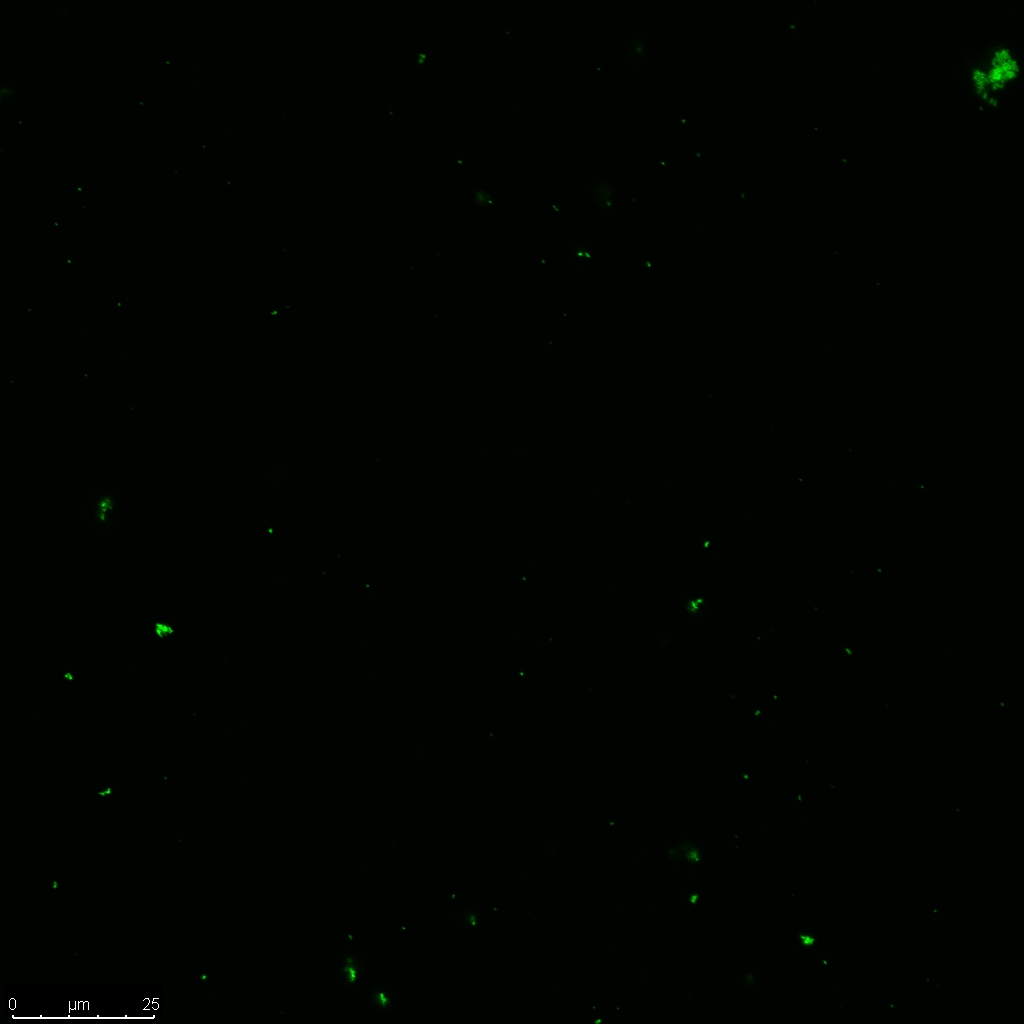

Supplement: Figure 2—source data 3. [file elife-94795-fig2-data3.zip › Figure 2B/replicate II/Figure 2B panel 6 Luc agg SSS replicate 2 photo 5.jpg]

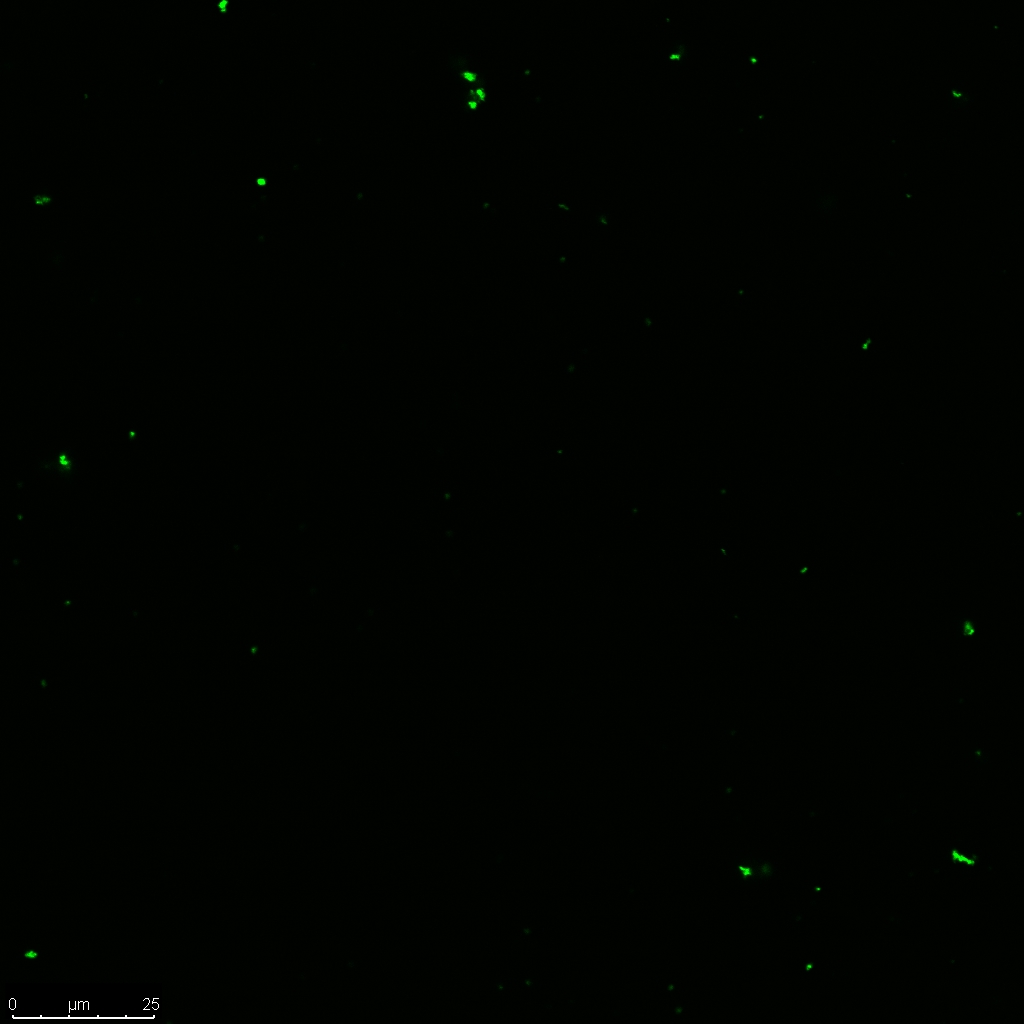

Supplement: Figure 2—source data 3. [file elife-94795-fig2-data3.zip › Figure 2B/replicate II/Figure 2B panel 6 Luc agg SSS replicate 2 photo 6.jpg]

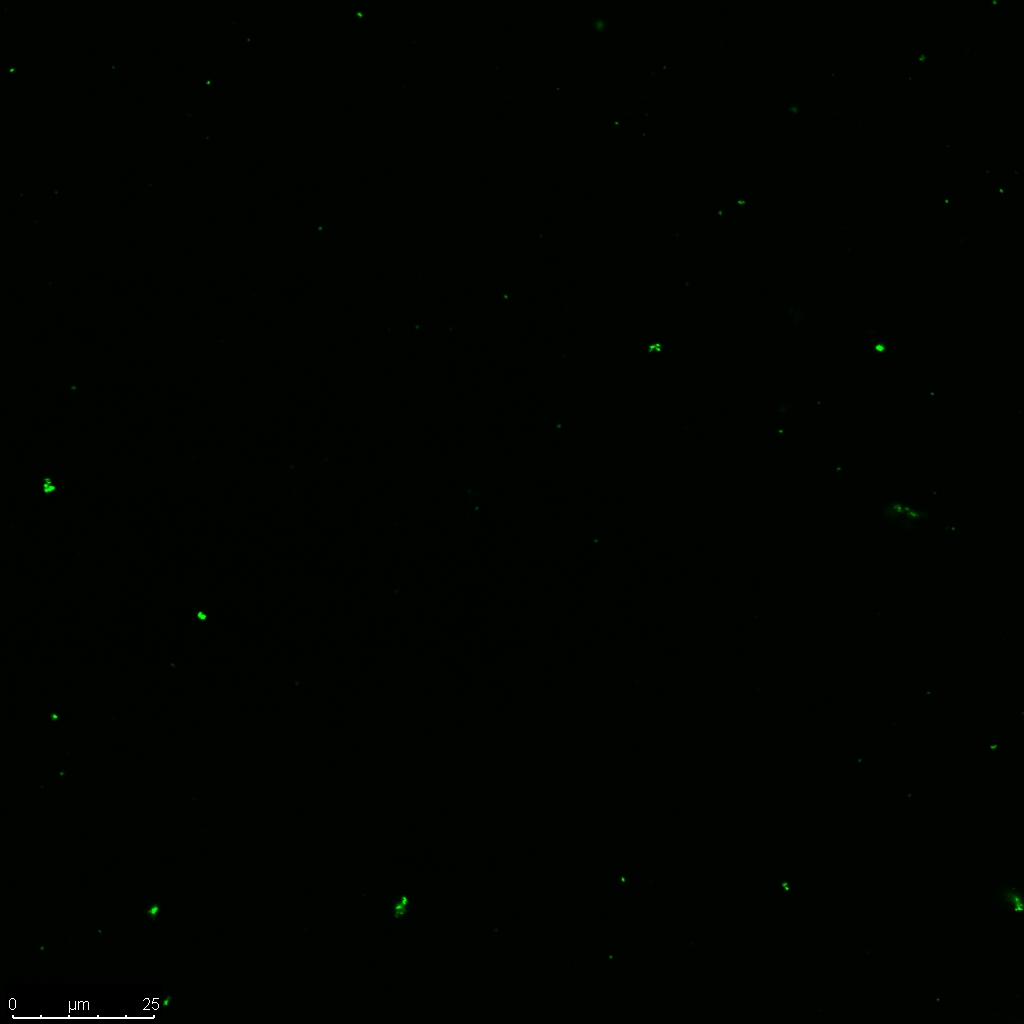

Supplement: Figure 2—source data 3. [file elife-94795-fig2-data3.zip › Figure 2B/replicate II/Figure 2B panel 6 Luc agg SSS replicate 2 photo 7.jpg]

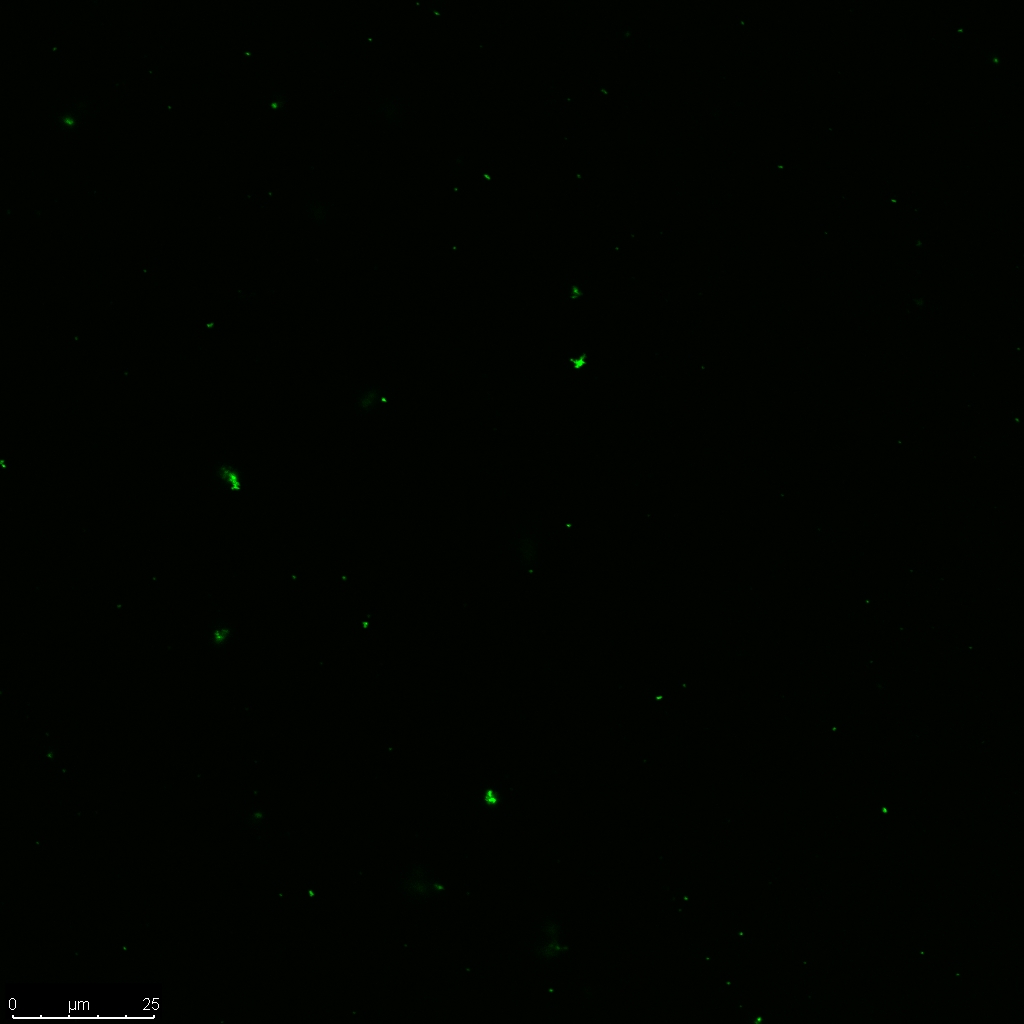

Supplement: Figure 2—source data 3. [file elife-94795-fig2-data3.zip › Figure 2B/replicate II/Figure 2B panel 6 Luc agg SSS replicate 2 photo 8.jpg]

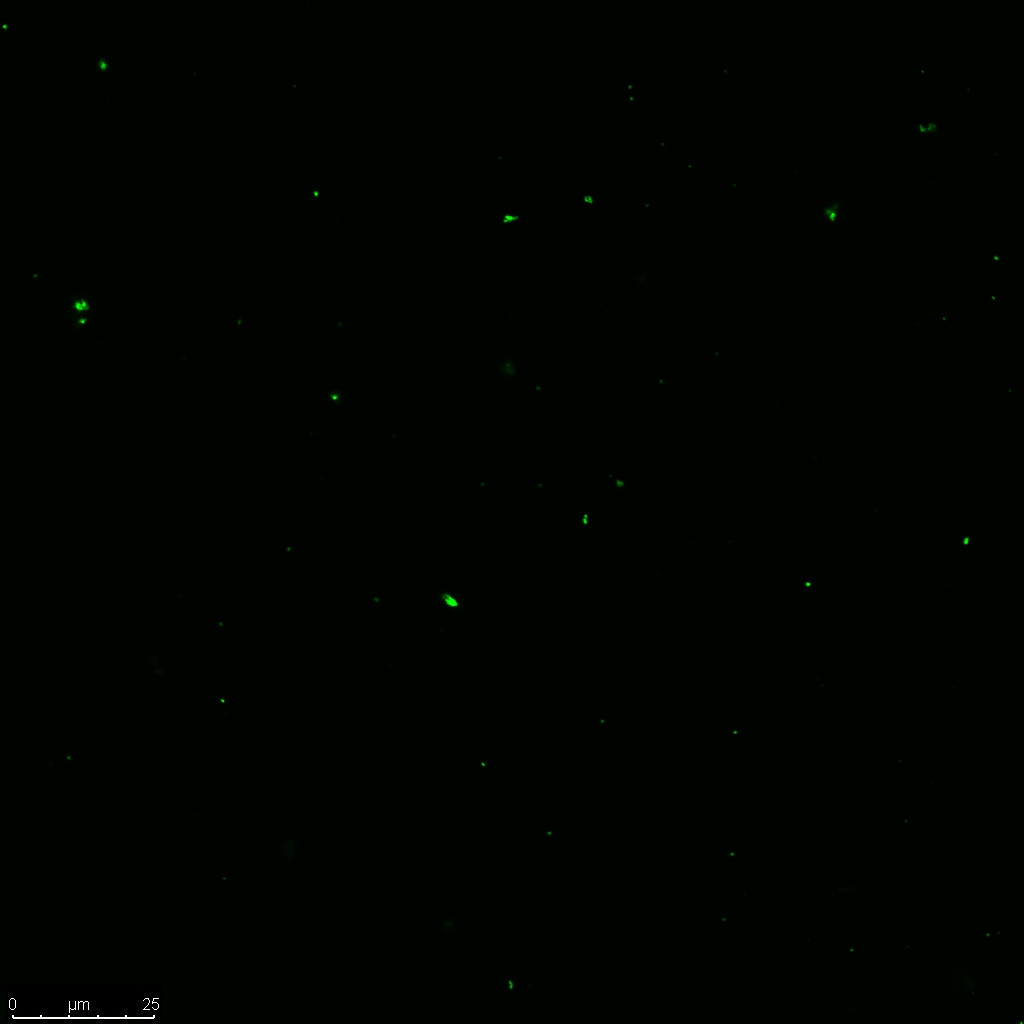

Supplement: Figure 2—source data 3. [file elife-94795-fig2-data3.zip › Figure 2B/replicate II/Figure 2B panel 6 Luc agg SSS replicate 2 photo 9.jpg]

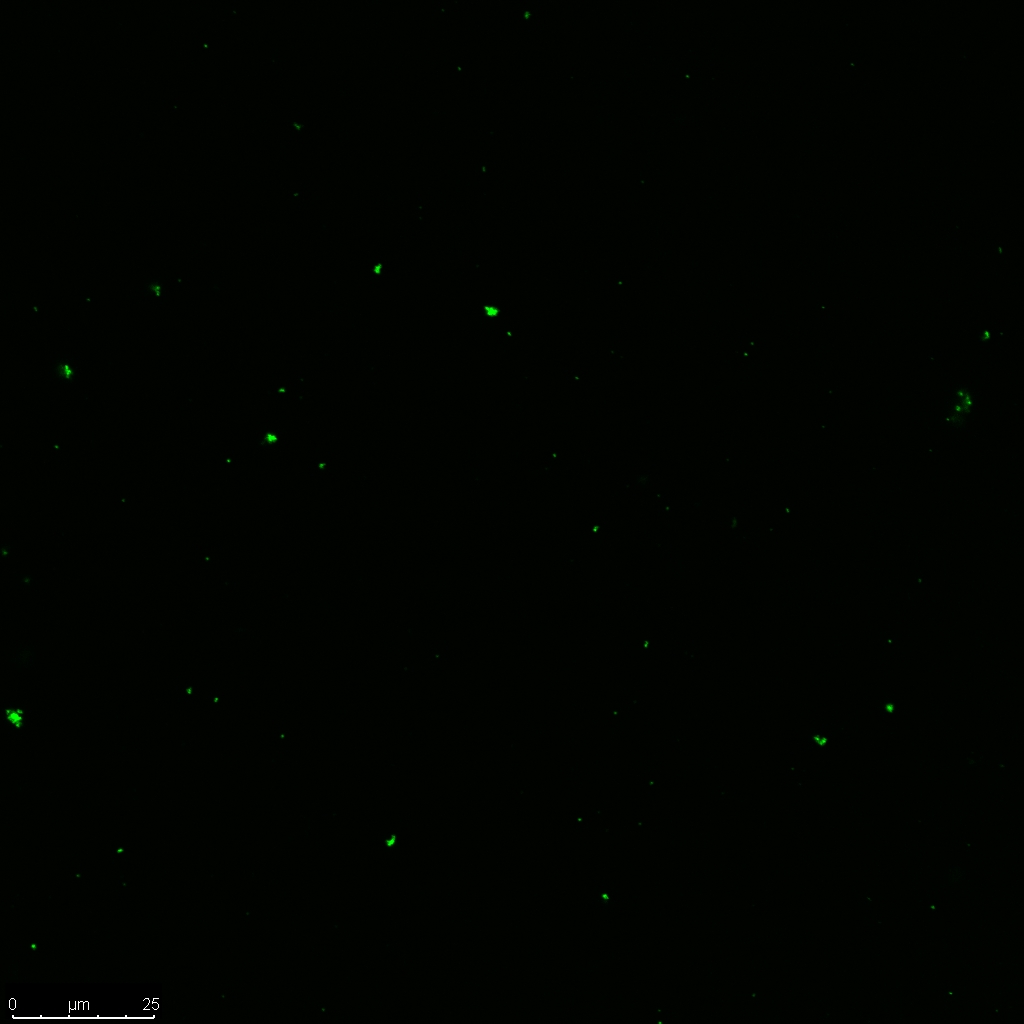

Supplement: Figure 2—source data 3. [file elife-94795-fig2-data3.zip › Figure 2B/replicate II/Figure 2B panel 6 Luc agg SSS replicate 2 photo 10.jpg]

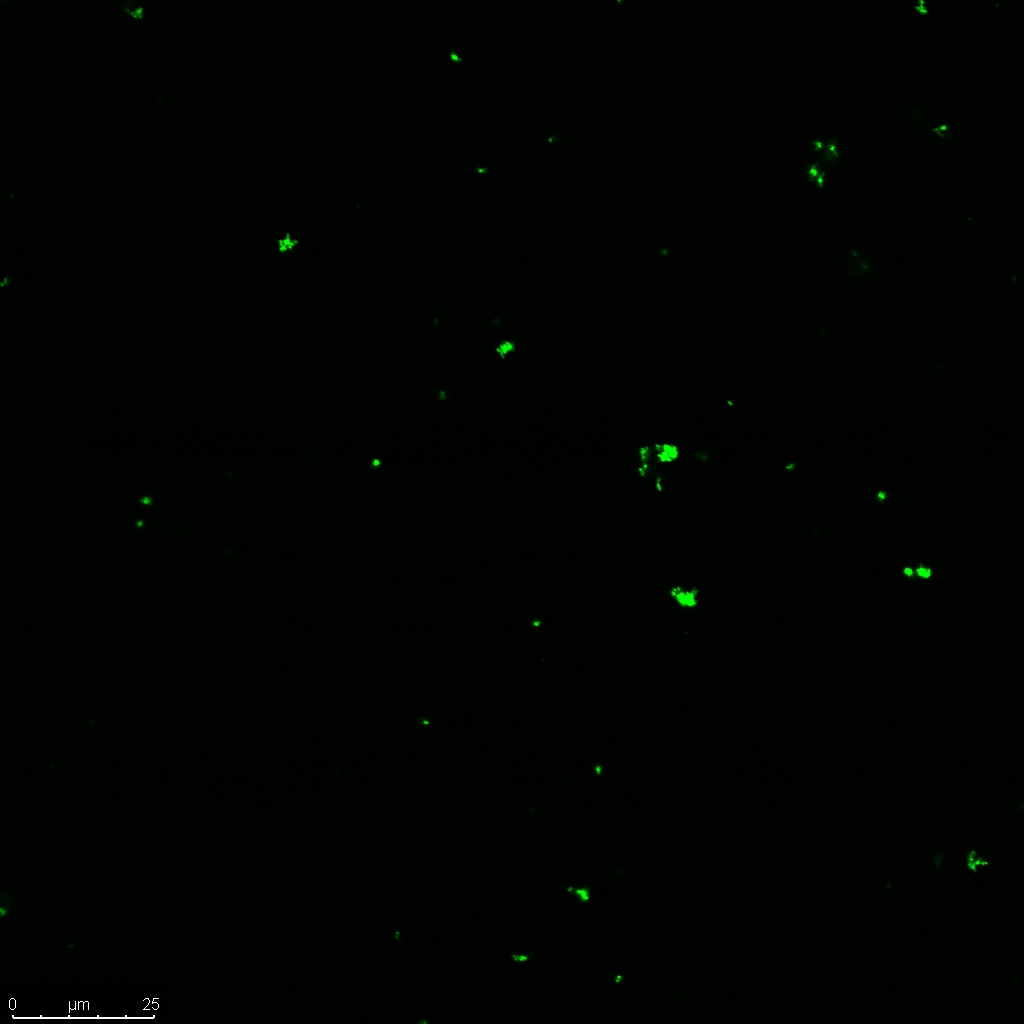

Supplement: Figure 2—source data 3. [file elife-94795-fig2-data3.zip › Figure 2B/replicate II/Figure 2B panel 5 Luc agg SSS replicate 2 photo 1.jpg]

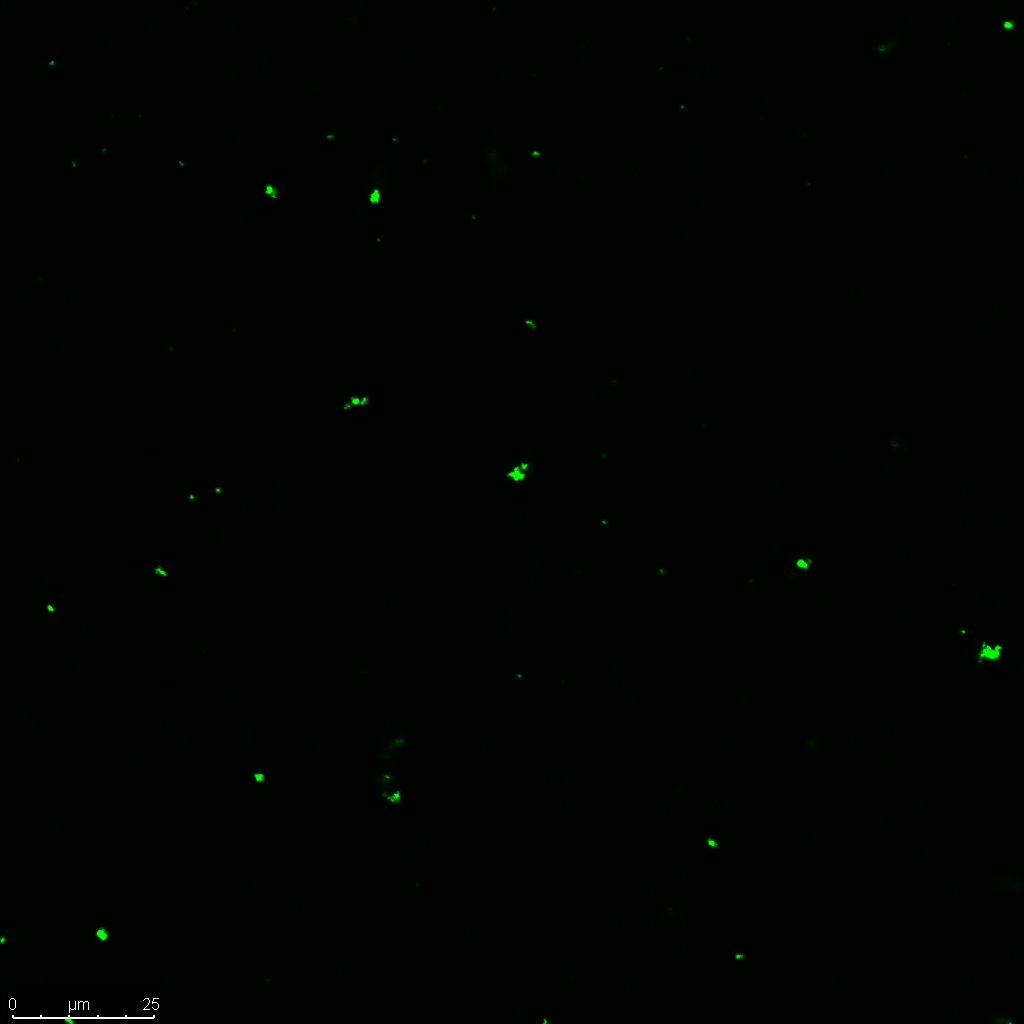

Supplement: Figure 2—source data 3. [file elife-94795-fig2-data3.zip › Figure 2B/replicate II/Figure 2B panel 5 Luc agg SSS replicate 2 photo 2.jpg]

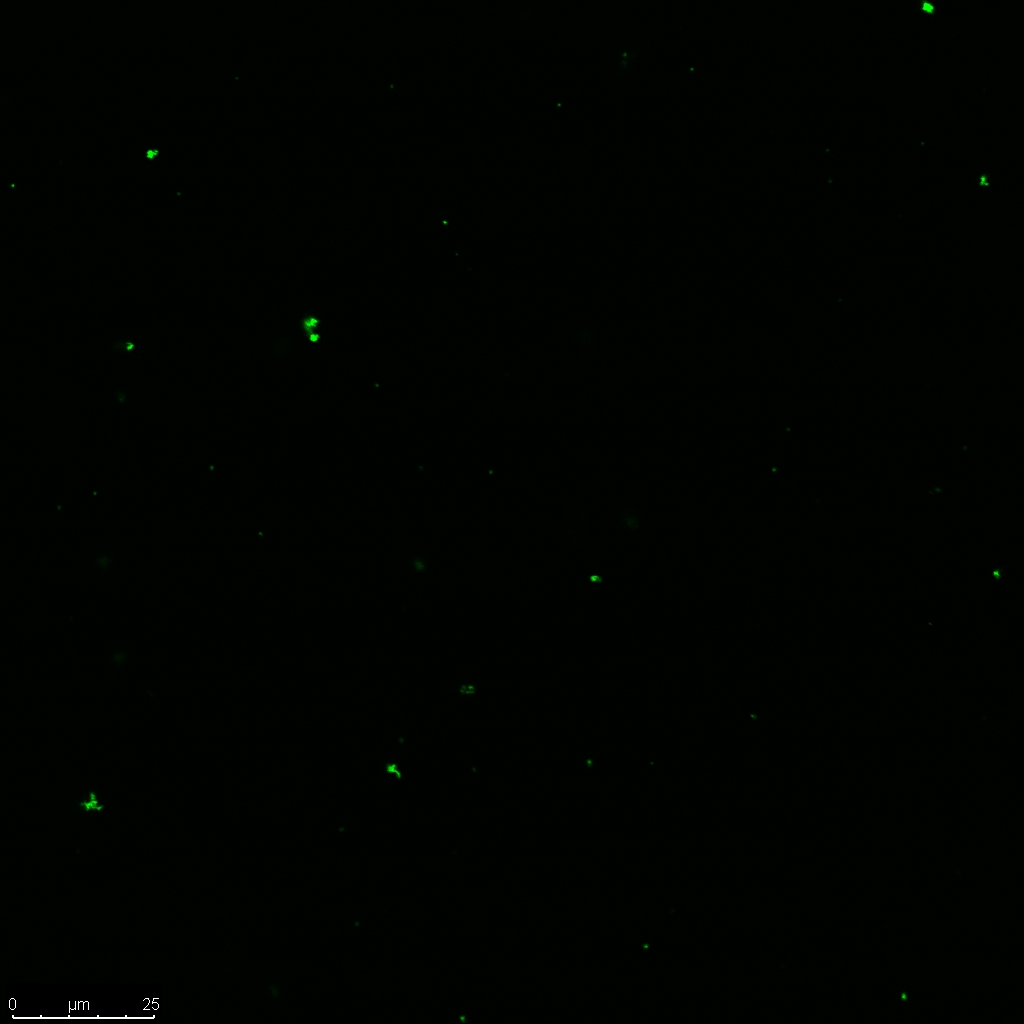

Supplement: Figure 2—source data 3. [file elife-94795-fig2-data3.zip › Figure 2B/replicate II/Figure 2B panel 5 Luc agg SSS replicate 2 photo 3.jpg]

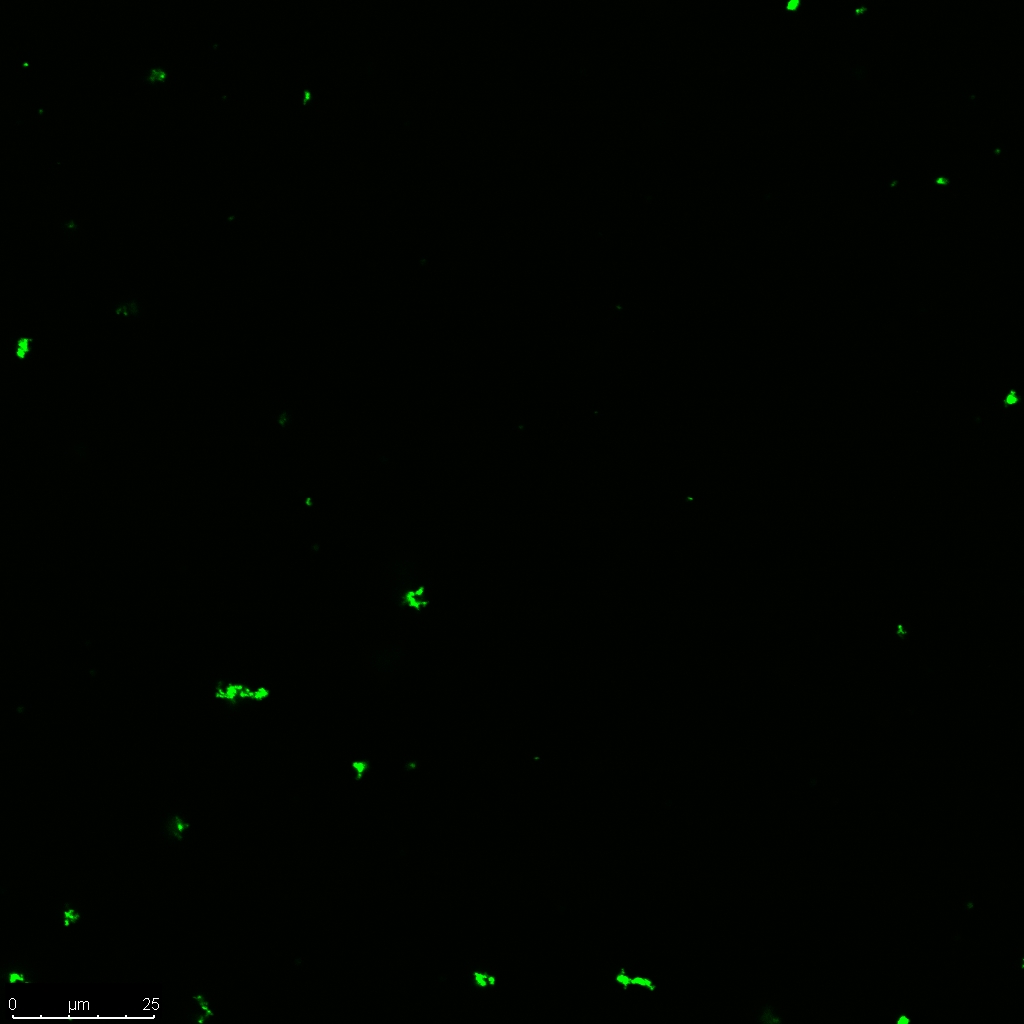

Supplement: Figure 2—source data 3. [file elife-94795-fig2-data3.zip › Figure 2B/replicate II/Figure 2B panel 5 Luc agg SSS replicate 2 photo 4.jpg]

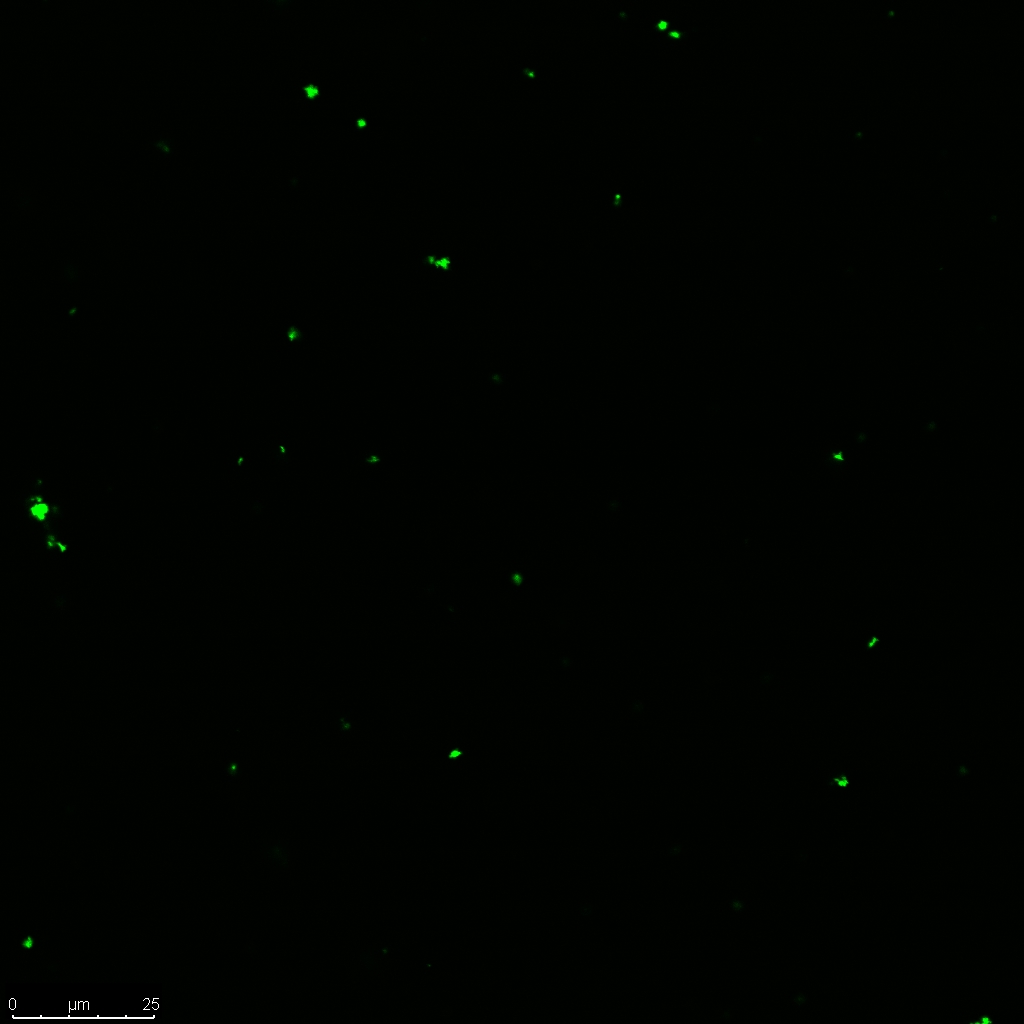

Supplement: Figure 2—source data 3. [file elife-94795-fig2-data3.zip › Figure 2B/replicate II/Figure 2B panel 5 Luc agg SSS replicate 2 photo 5.jpg]

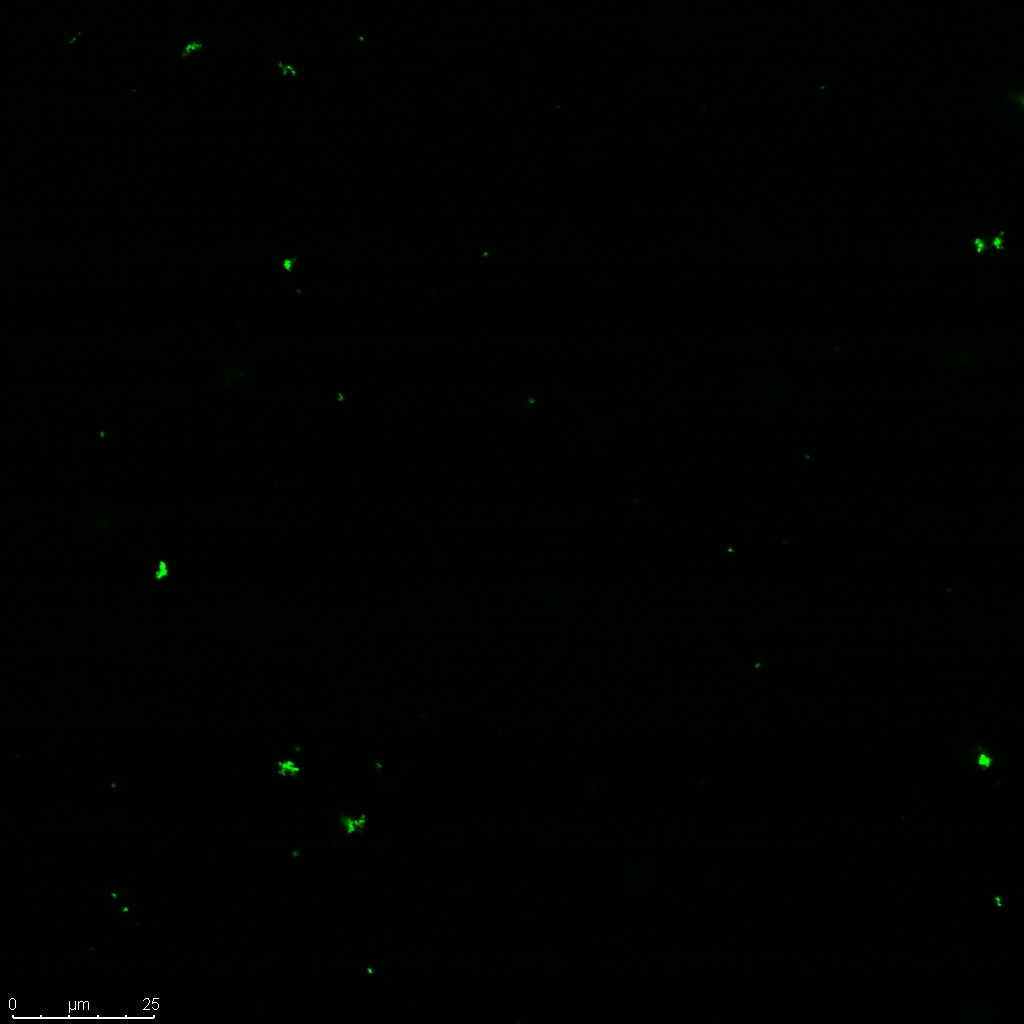

Supplement: Figure 2—source data 3. [file elife-94795-fig2-data3.zip › Figure 2B/replicate II/Figure 2B panel 5 Luc agg SSS replicate 2 photo 6.jpg]

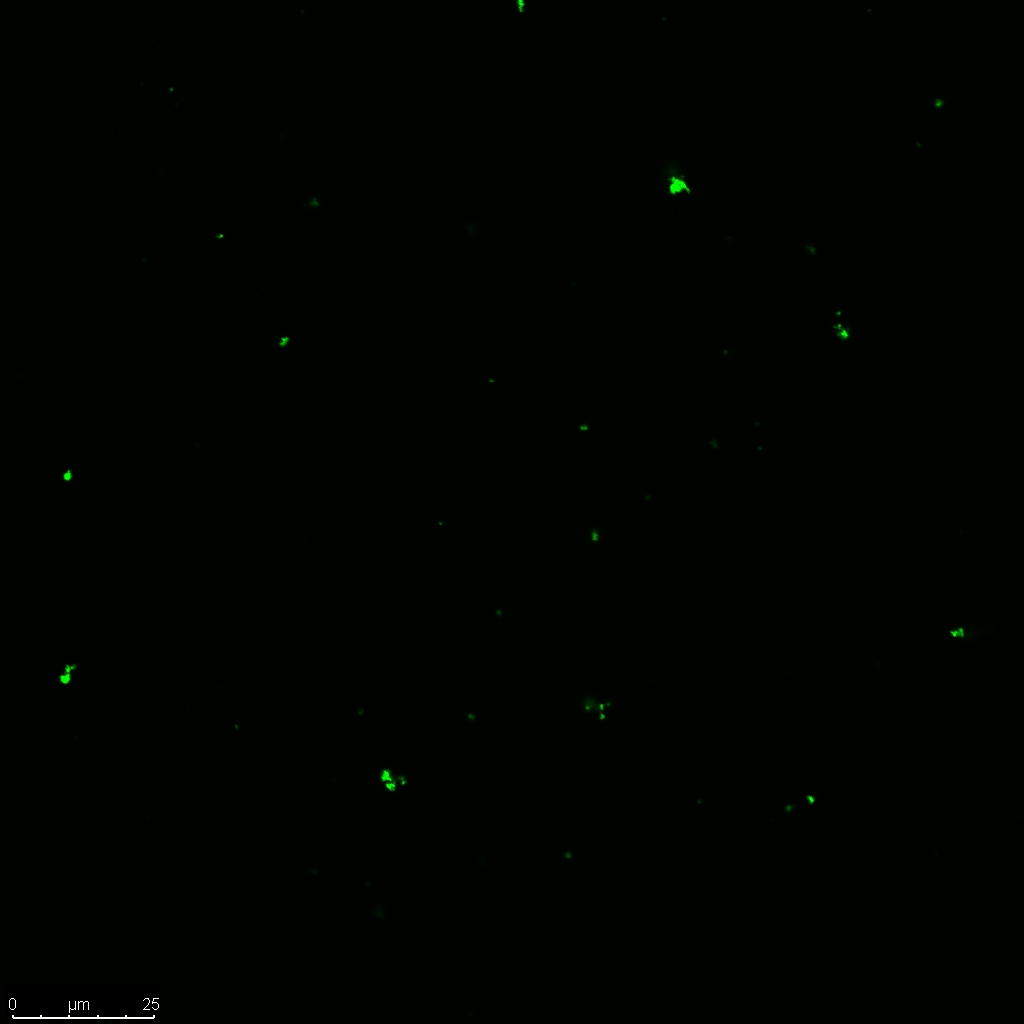

Supplement: Figure 2—source data 3. [file elife-94795-fig2-data3.zip › Figure 2B/replicate II/Figure 2B panel 5 Luc agg SSS replicate 2 photo 7.jpg]

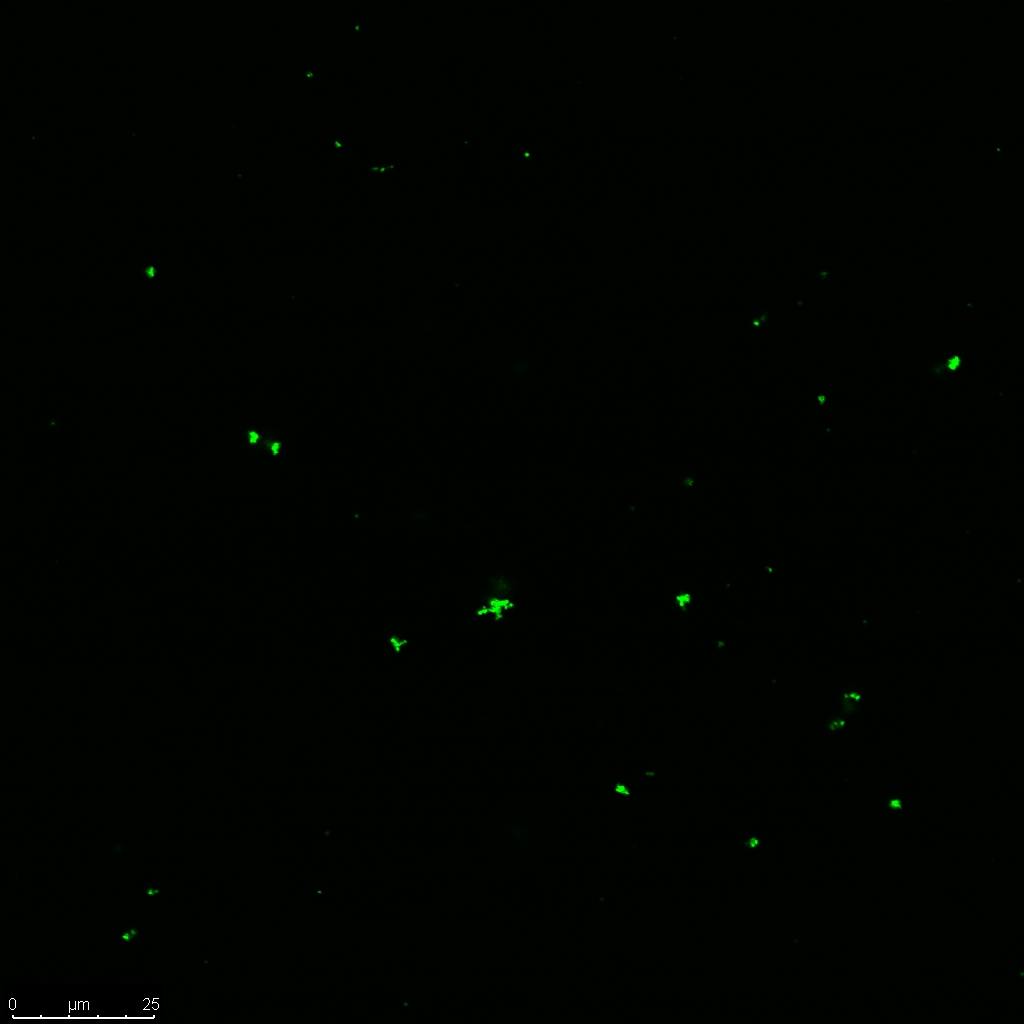

Supplement: Figure 2—source data 3. [file elife-94795-fig2-data3.zip › Figure 2B/replicate II/Figure 2B panel 5 Luc agg SSS replicate 2 photo 8.jpg]

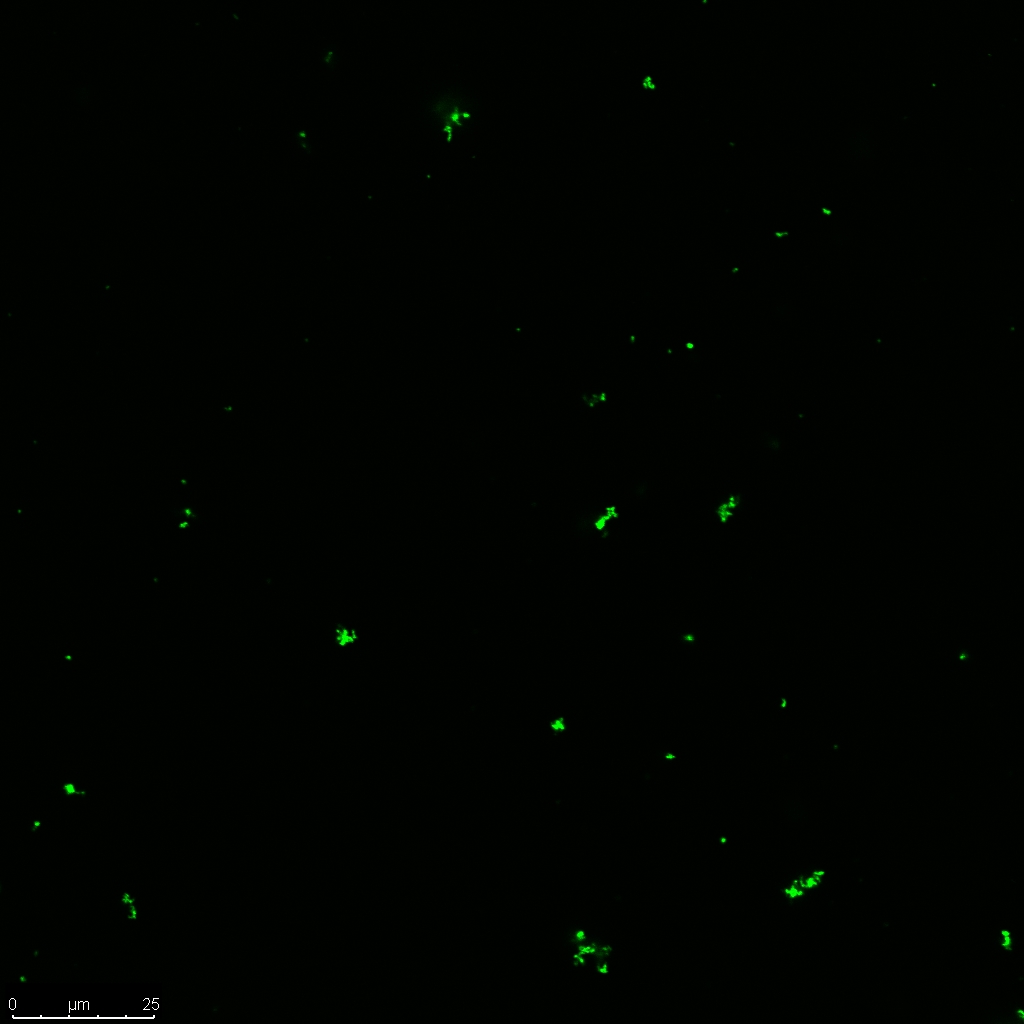

Supplement: Figure 2—source data 3. [file elife-94795-fig2-data3.zip › Figure 2B/replicate II/Figure 2B panel 5 Luc agg SSS replicate 2 photo 9.jpg]

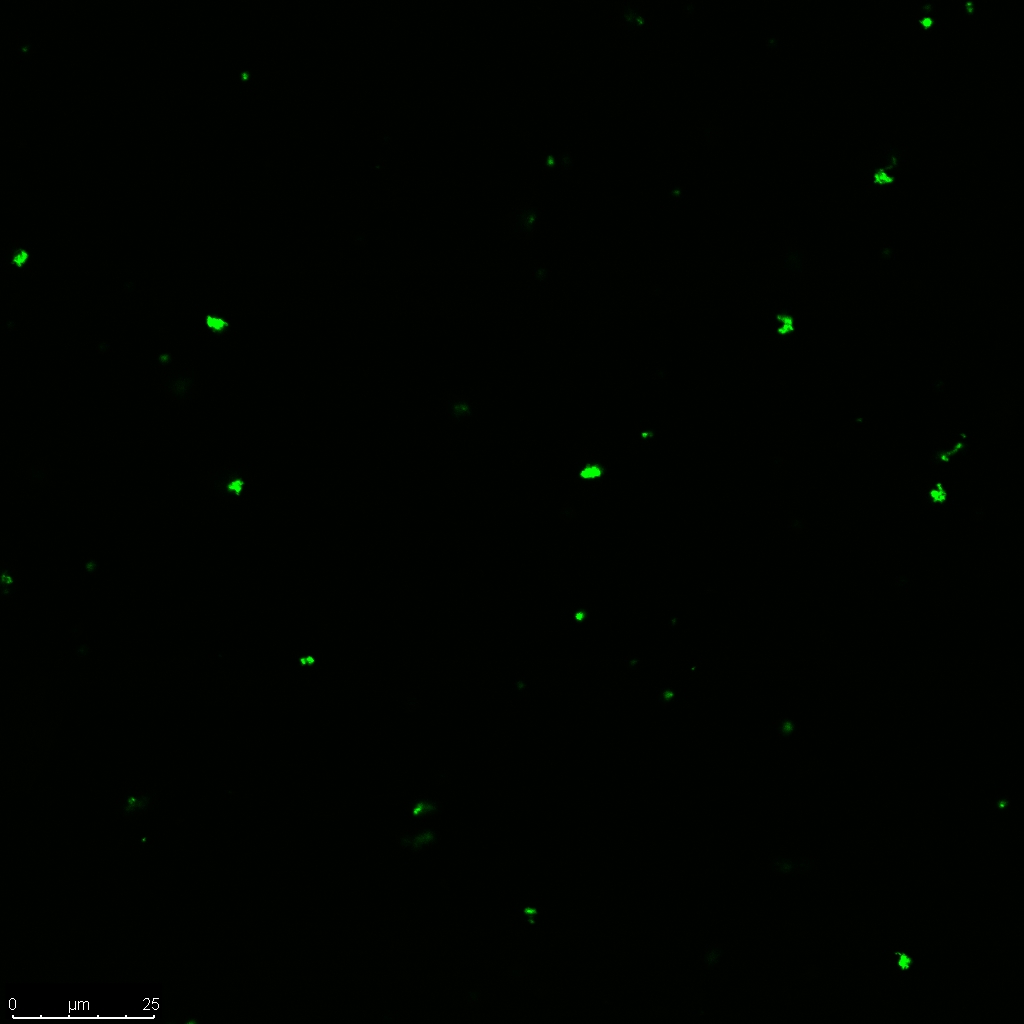

Supplement: Figure 2—source data 3. [file elife-94795-fig2-data3.zip › Figure 2B/replicate II/Figure 2B panel 5 Luc agg SSS replicate 2 photo 10.jpg]

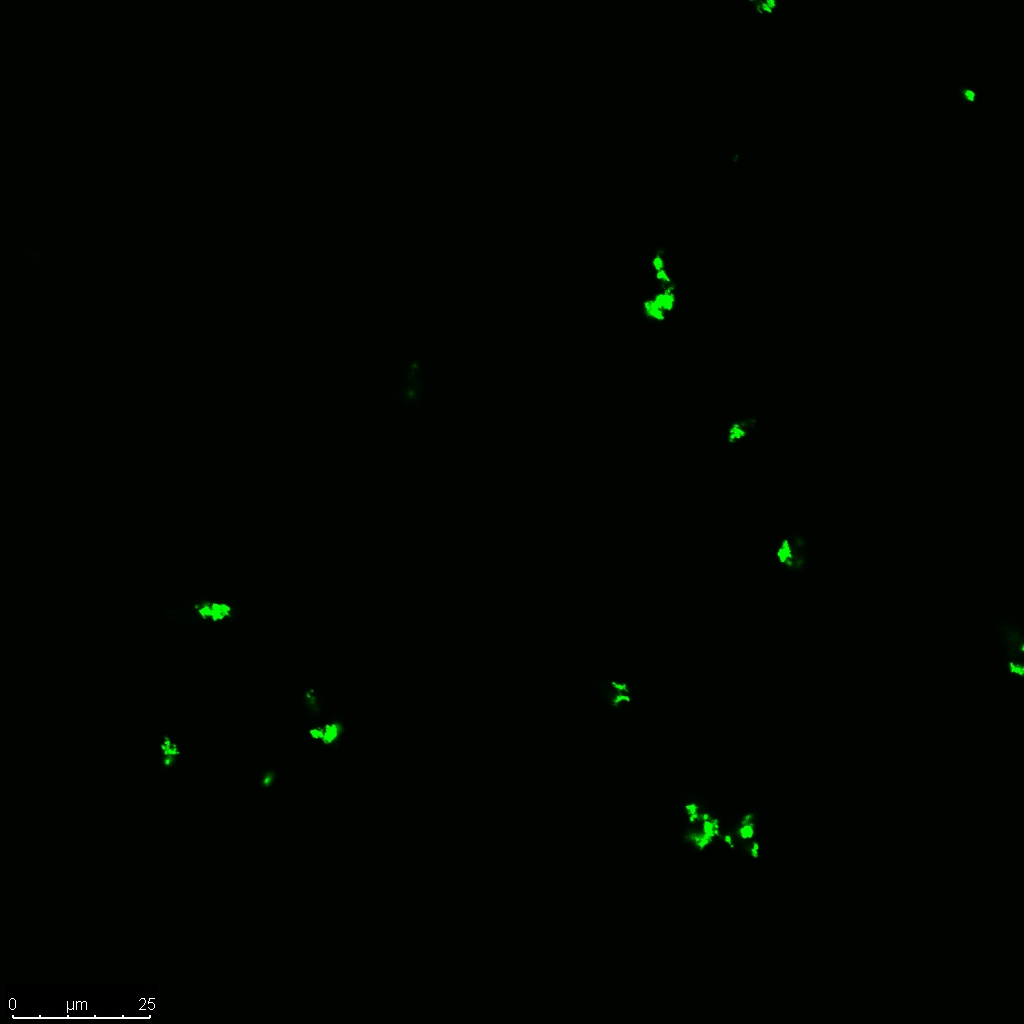

Supplement: Figure 2—source data 3. [file elife-94795-fig2-data3.zip › Figure 2B/replicate III/Figure 2B panel 2 Luc agg YS replicate 3 photo 10.jpg]

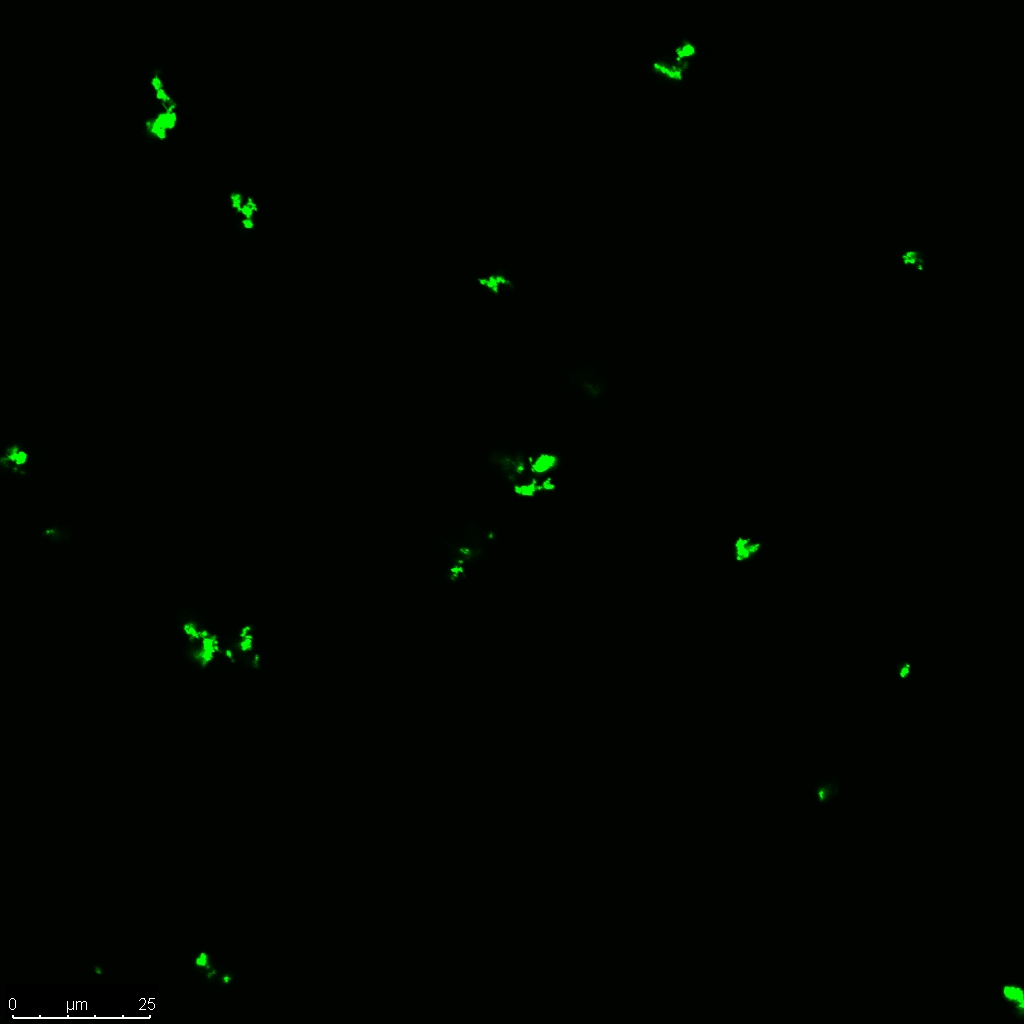

Supplement: Figure 2—source data 3. [file elife-94795-fig2-data3.zip › Figure 2B/replicate III/Figure 2B panel 2 Luc agg YS replicate 3 photo 9.jpg]

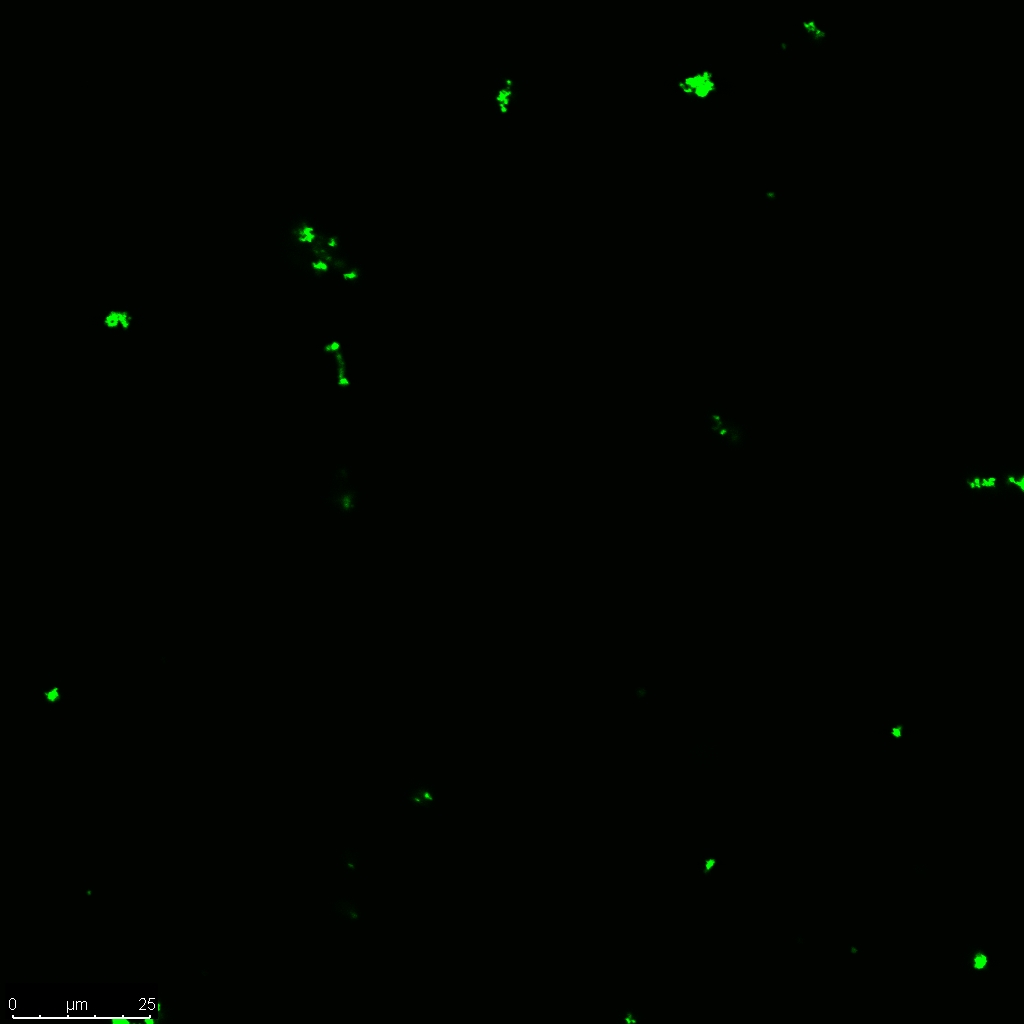

Supplement: Figure 2—source data 3. [file elife-94795-fig2-data3.zip › Figure 2B/replicate III/Figure 2B panel 2 Luc agg YS replicate 3 photo 8.jpg]

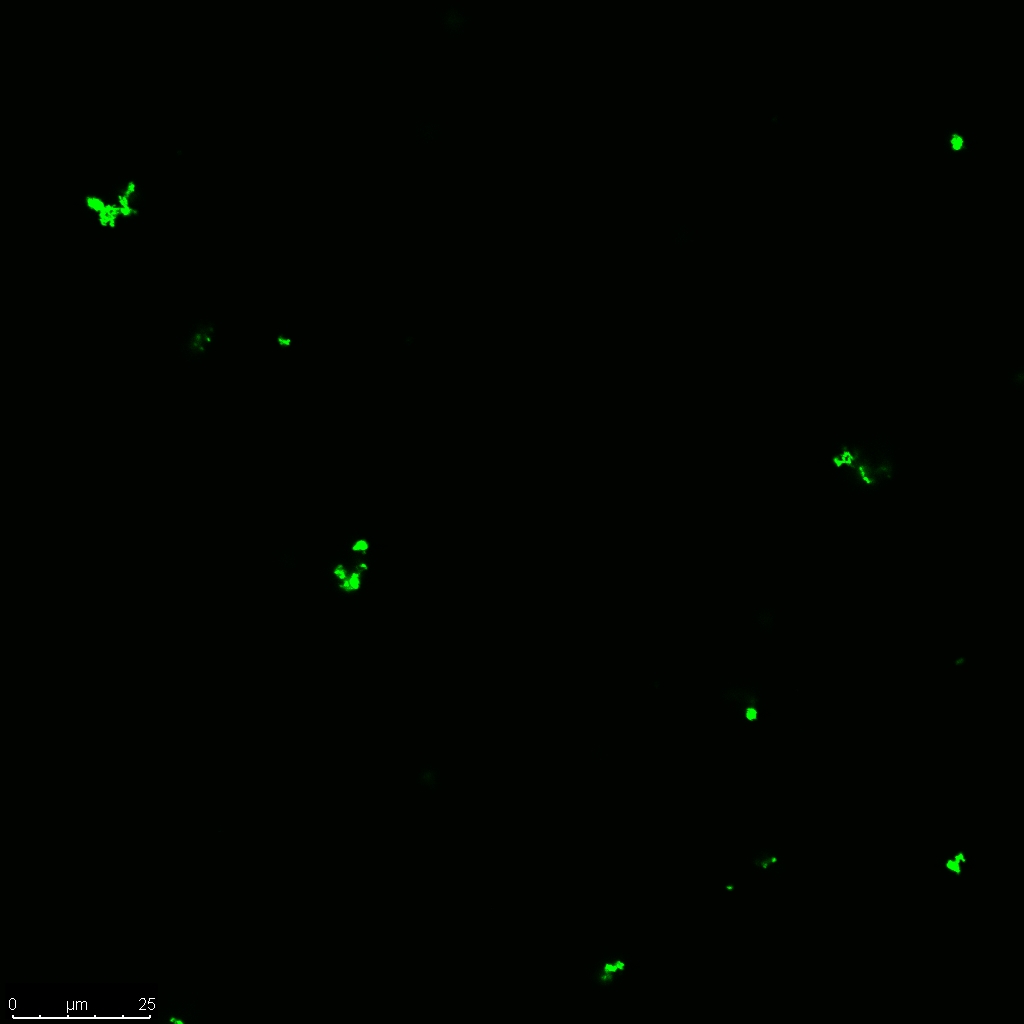

Supplement: Figure 2—source data 3. [file elife-94795-fig2-data3.zip › Figure 2B/replicate III/Figure 2B panel 2 Luc agg YS replicate 3 photo 7.jpg]

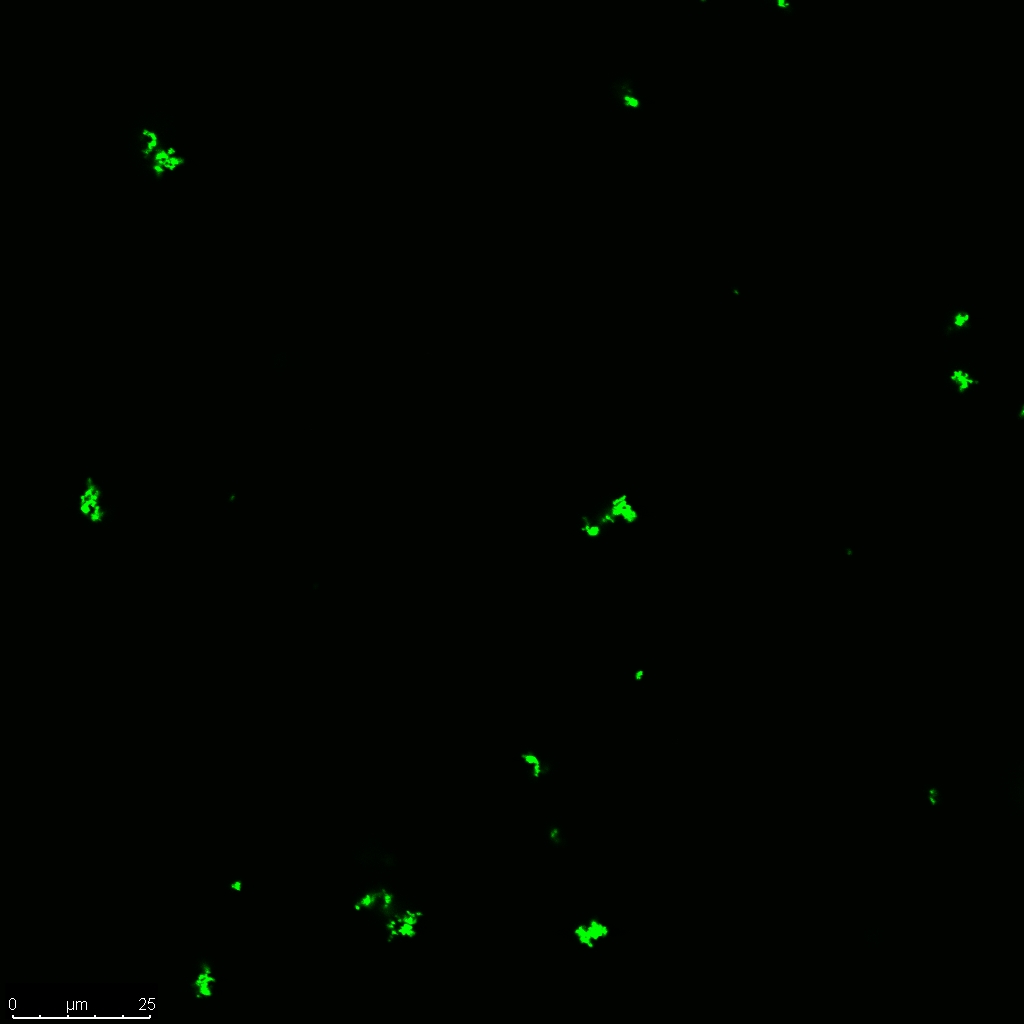

Supplement: Figure 2—source data 3. [file elife-94795-fig2-data3.zip › Figure 2B/replicate III/Figure 2B panel 2 Luc agg YS replicate 3 photo 6.jpg]

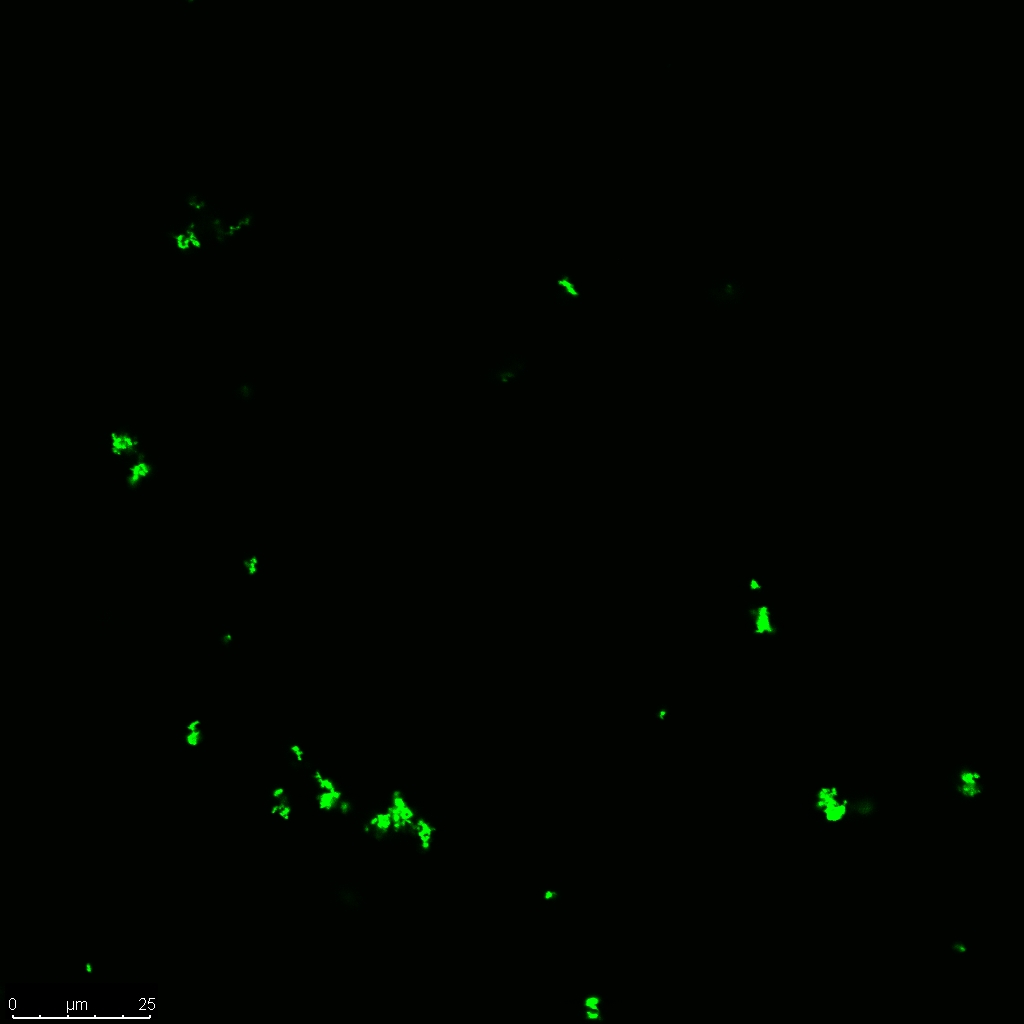

Supplement: Figure 2—source data 3. [file elife-94795-fig2-data3.zip › Figure 2B/replicate III/Figure 2B panel 2 Luc agg YS replicate 3 photo 5.jpg]

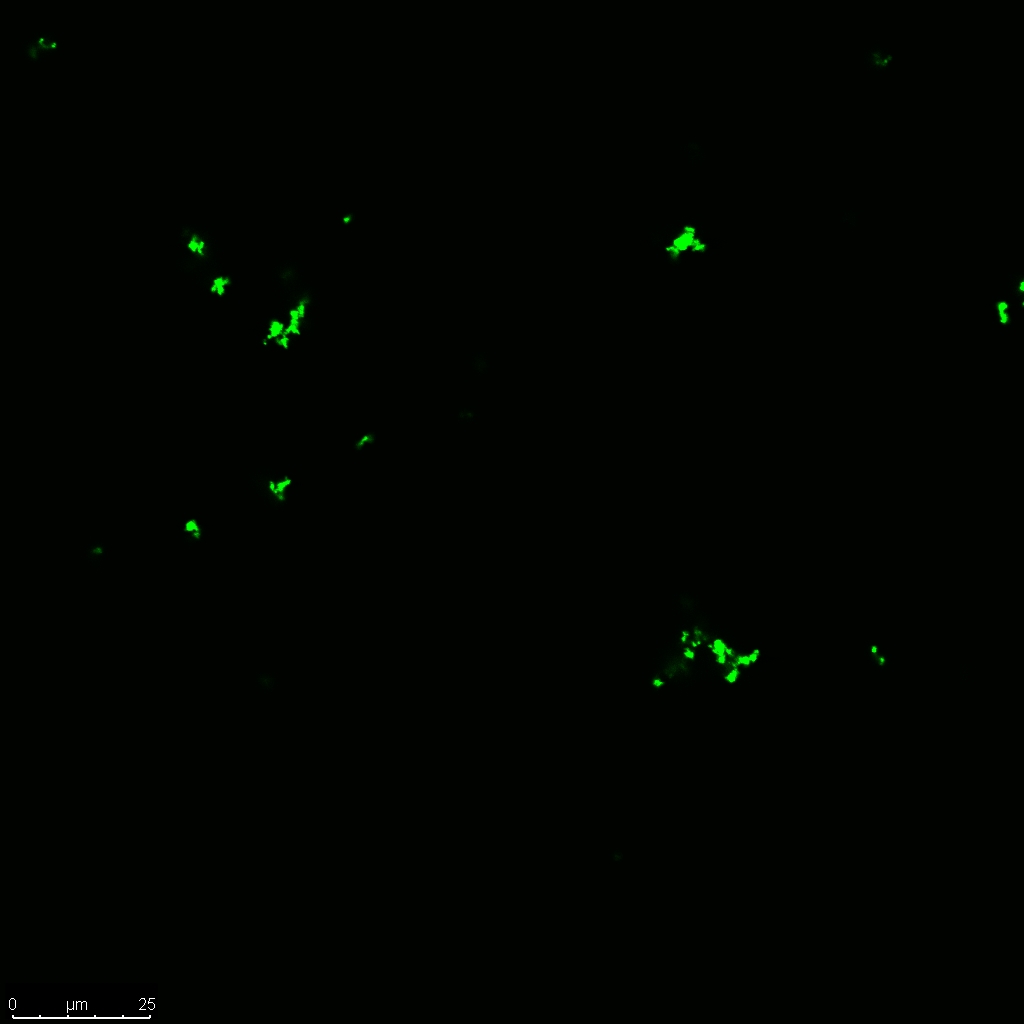

Supplement: Figure 2—source data 3. [file elife-94795-fig2-data3.zip › Figure 2B/replicate III/Figure 2B panel 2 Luc agg YS replicate 3 photo 4.jpg]

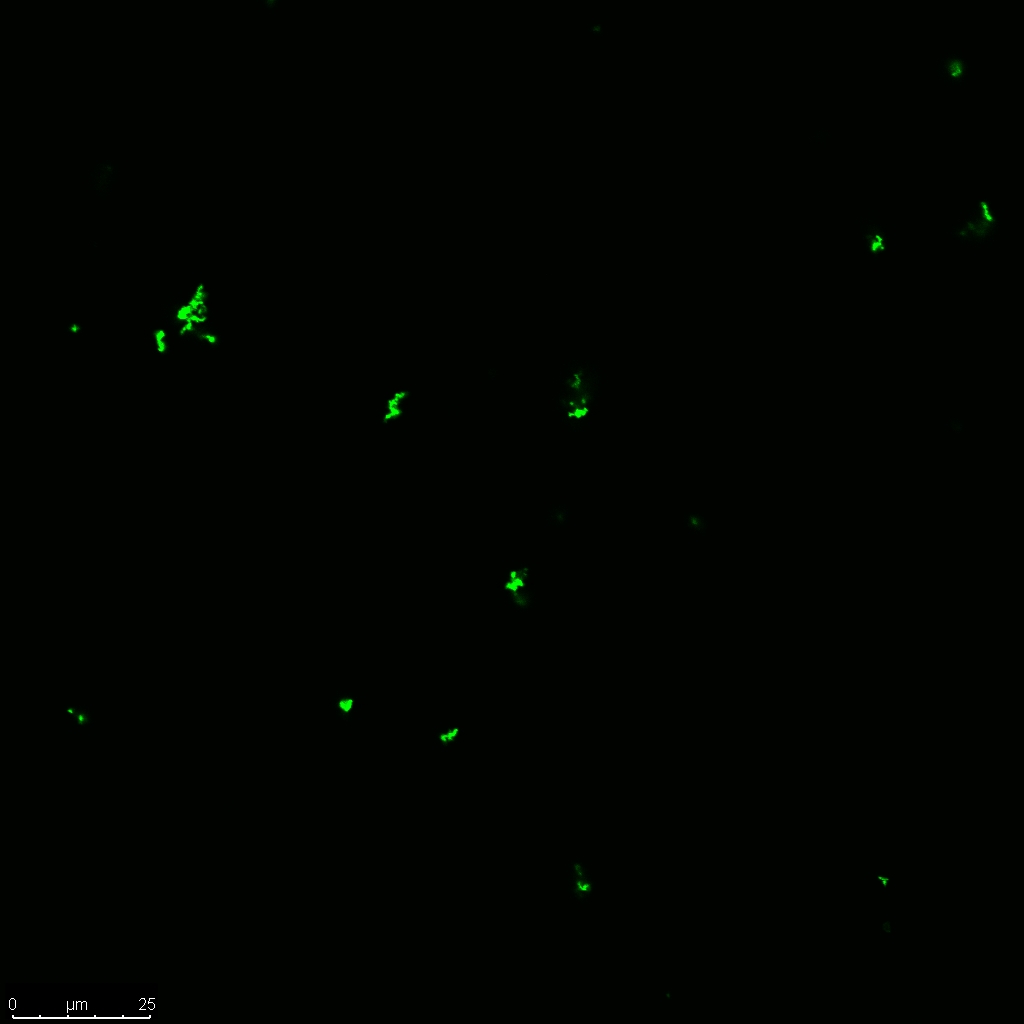

Supplement: Figure 2—source data 3. [file elife-94795-fig2-data3.zip › Figure 2B/replicate III/Figure 2B panel 2 Luc agg YS replicate 3 photo 3.jpg]

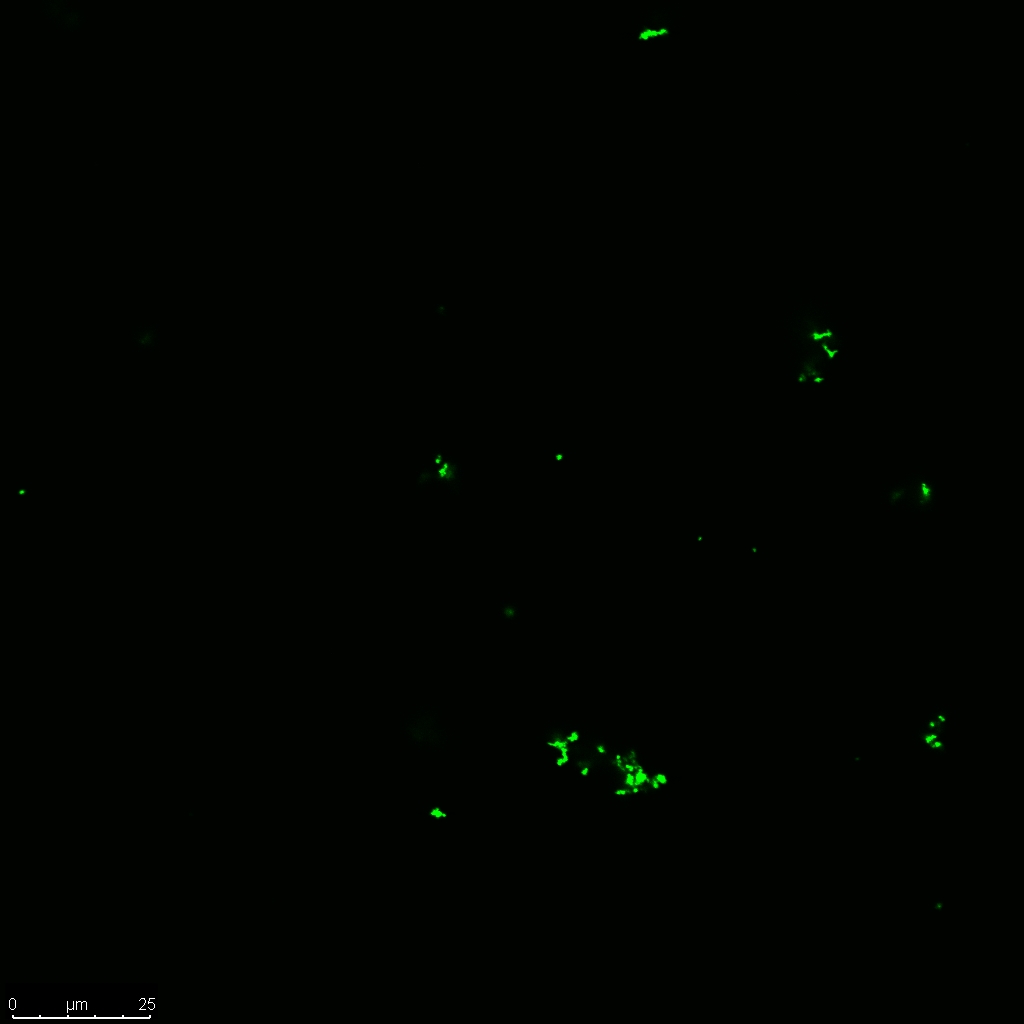

Supplement: Figure 2—source data 3. [file elife-94795-fig2-data3.zip › Figure 2B/replicate III/Figure 2B panel 2 Luc agg YS replicate 3 photo 2.jpg]

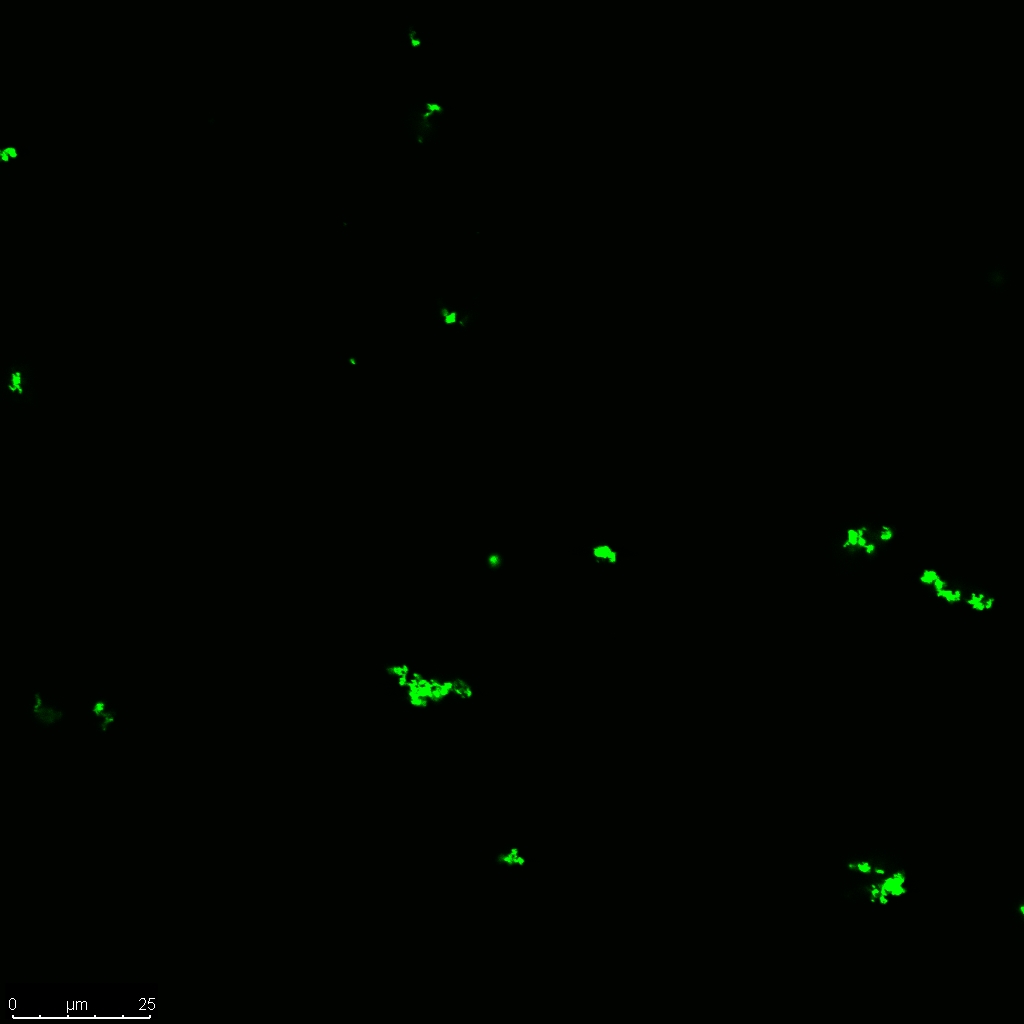

Supplement: Figure 2—source data 3. [file elife-94795-fig2-data3.zip › Figure 2B/replicate III/Figure 2B panel 2 Luc agg YS replicate 3 photo 1.jpg]

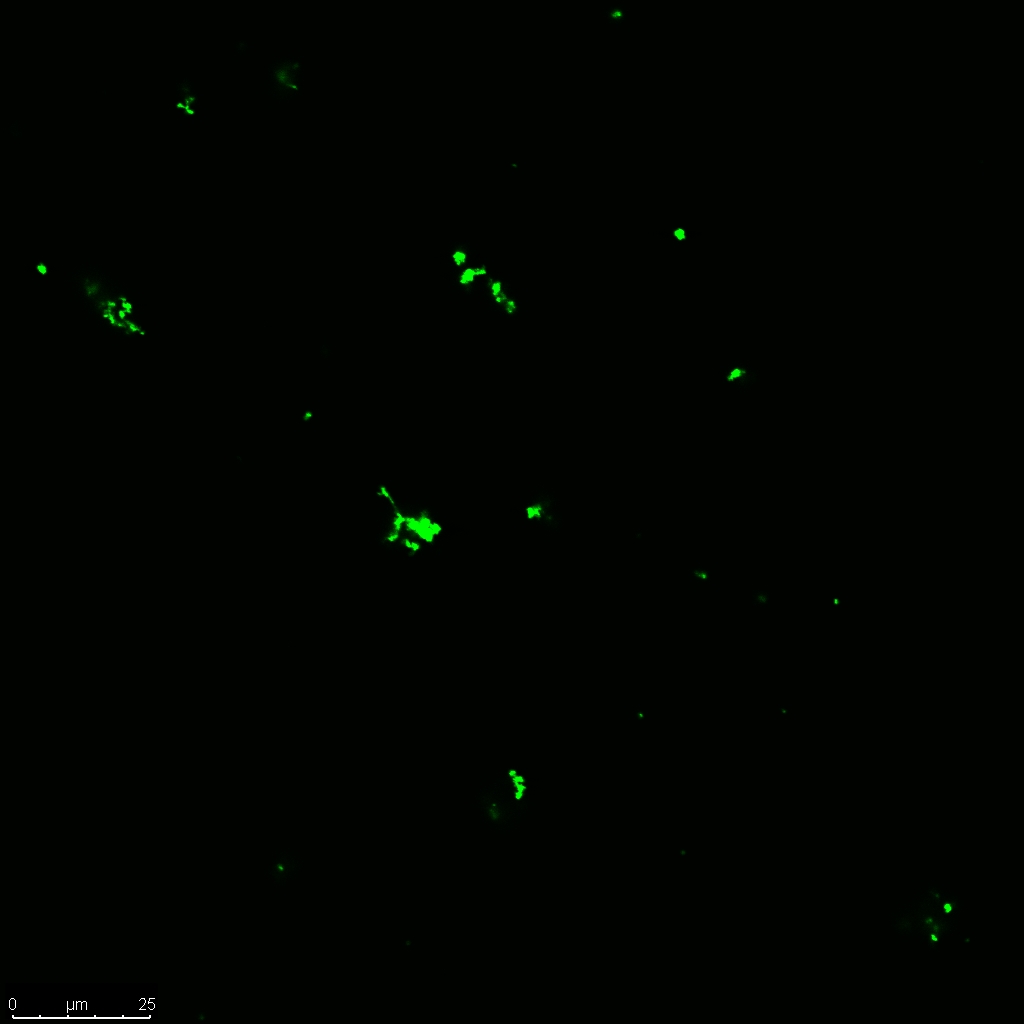

Supplement: Figure 2—source data 3. [file elife-94795-fig2-data3.zip › Figure 2B/replicate III/Figure 2B panel 3 Luc agg YSS replicate 3 photo 10.jpg]

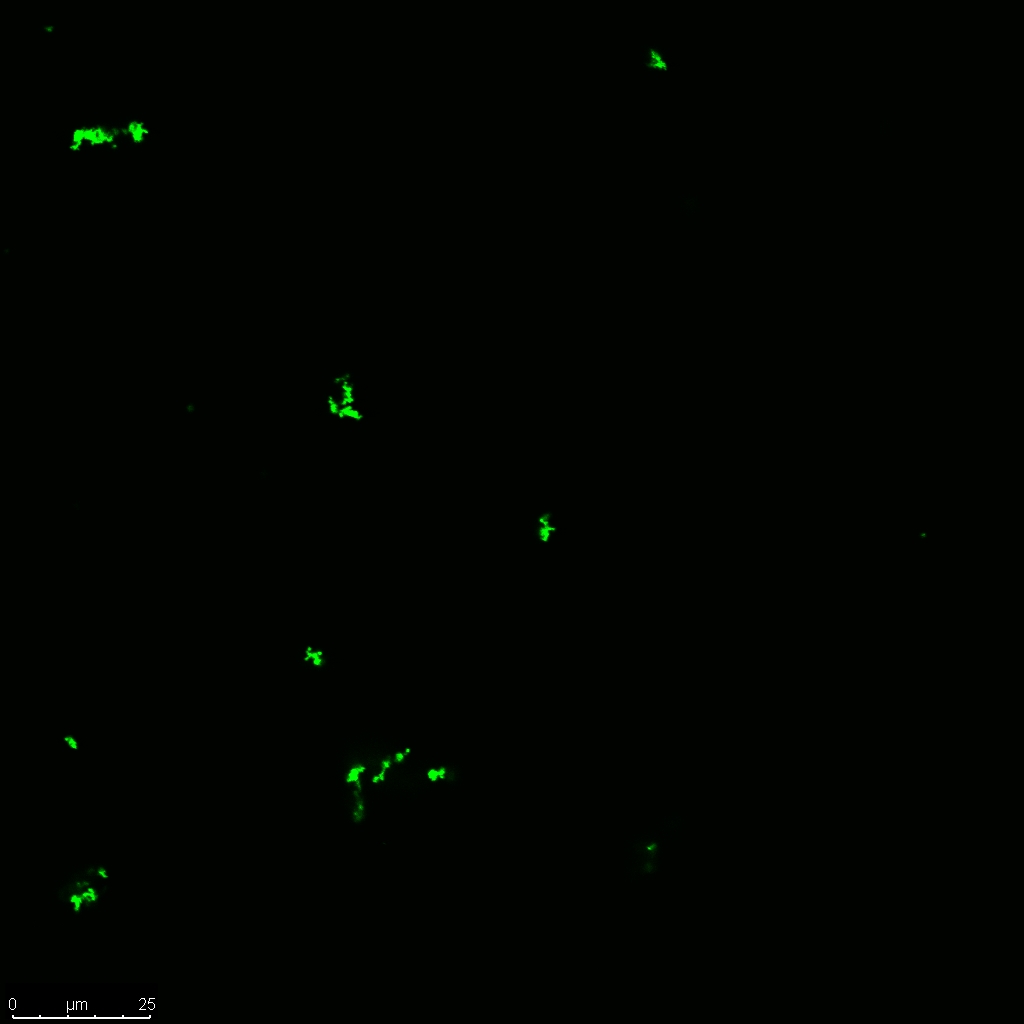

Supplement: Figure 2—source data 3. [file elife-94795-fig2-data3.zip › Figure 2B/replicate III/Figure 2B panel 3 Luc agg YSS replicate 3 photo 9.jpg]

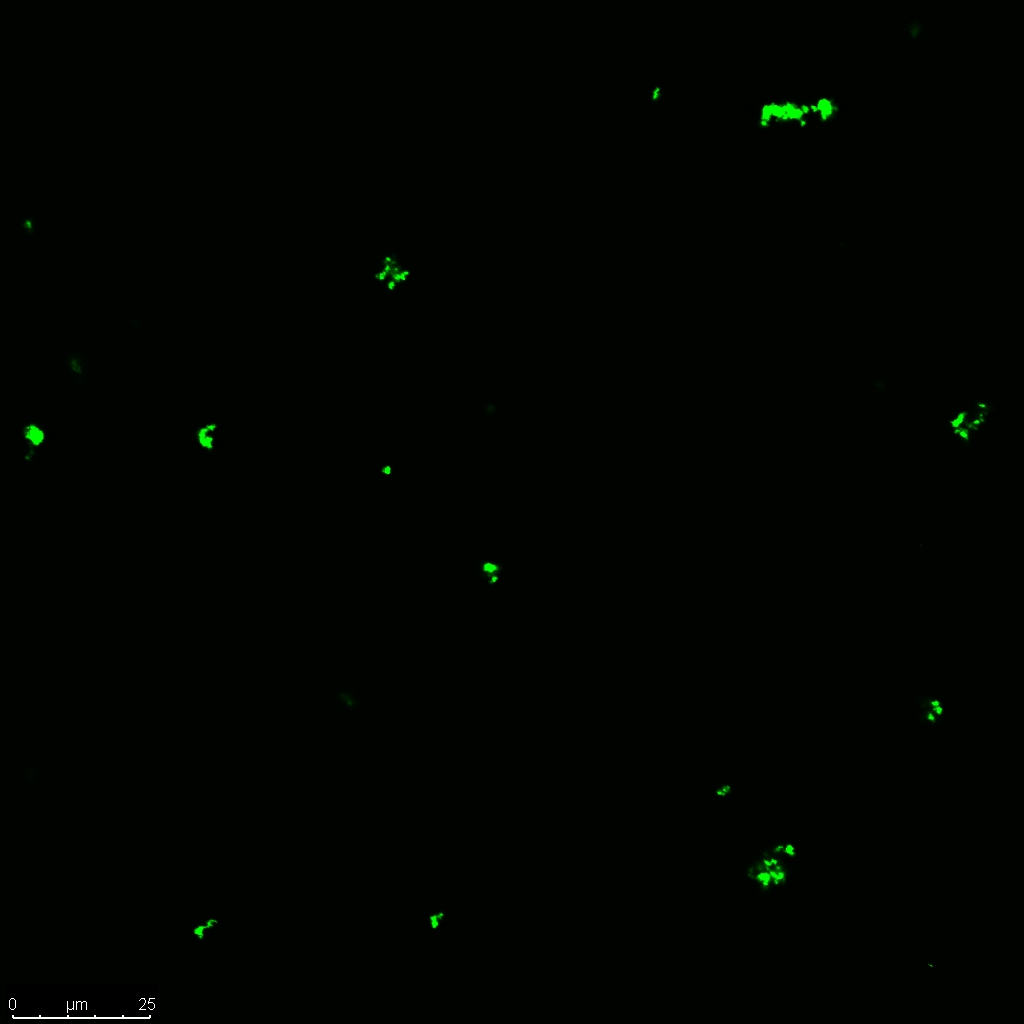

Supplement: Figure 2—source data 3. [file elife-94795-fig2-data3.zip › Figure 2B/replicate III/Figure 2B panel 3 Luc agg YSS replicate 3 photo 8.jpg]

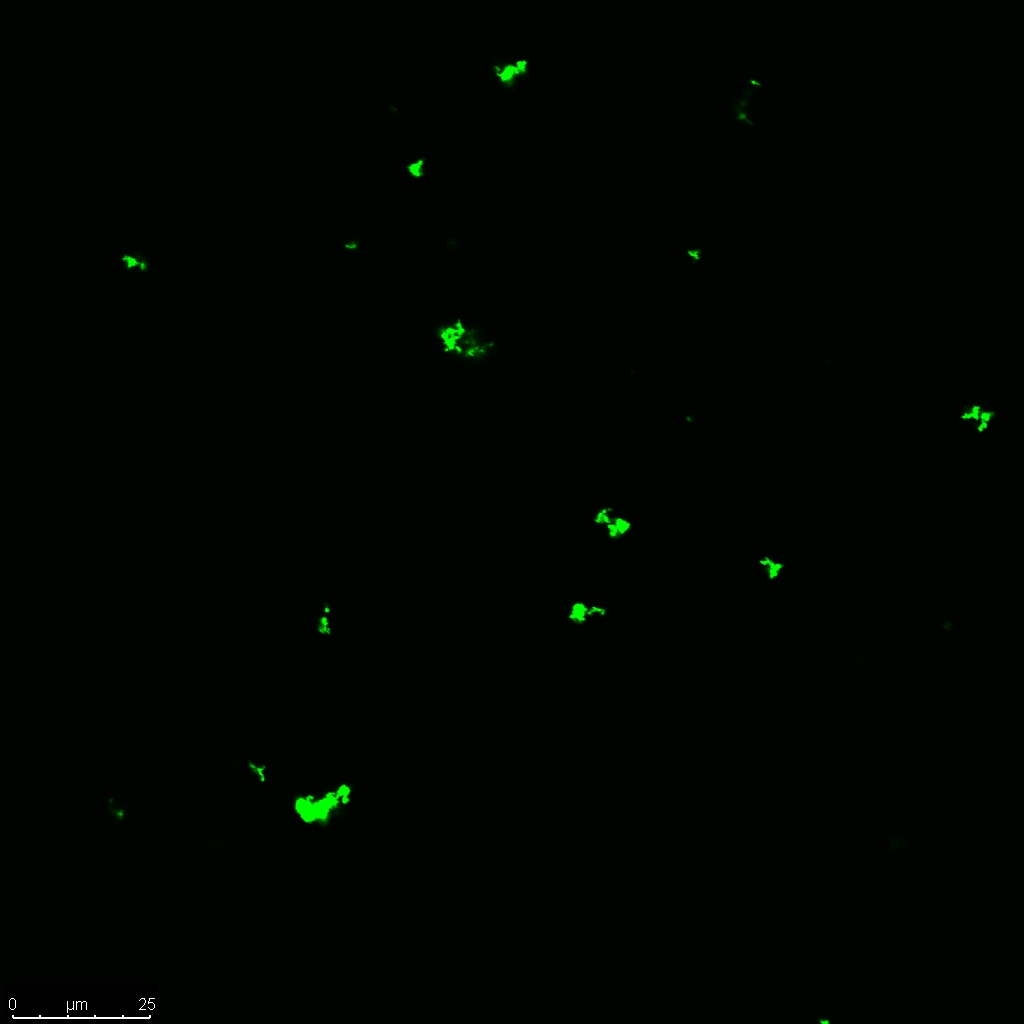

Supplement: Figure 2—source data 3. [file elife-94795-fig2-data3.zip › Figure 2B/replicate III/Figure 2B panel 3 Luc agg YSS replicate 3 photo 7.jpg]

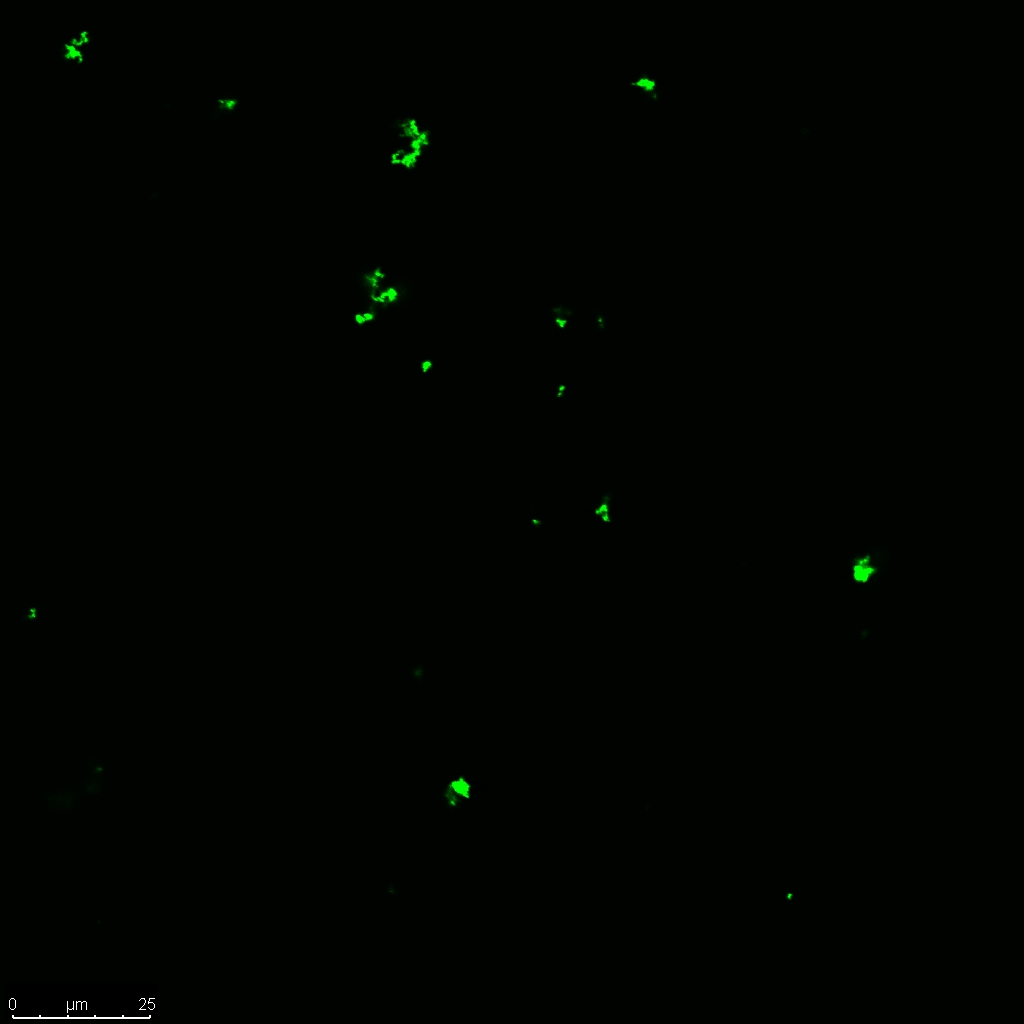

Supplement: Figure 2—source data 3. [file elife-94795-fig2-data3.zip › Figure 2B/replicate III/Figure 2B panel 3 Luc agg YSS replicate 3 photo 6.jpg]

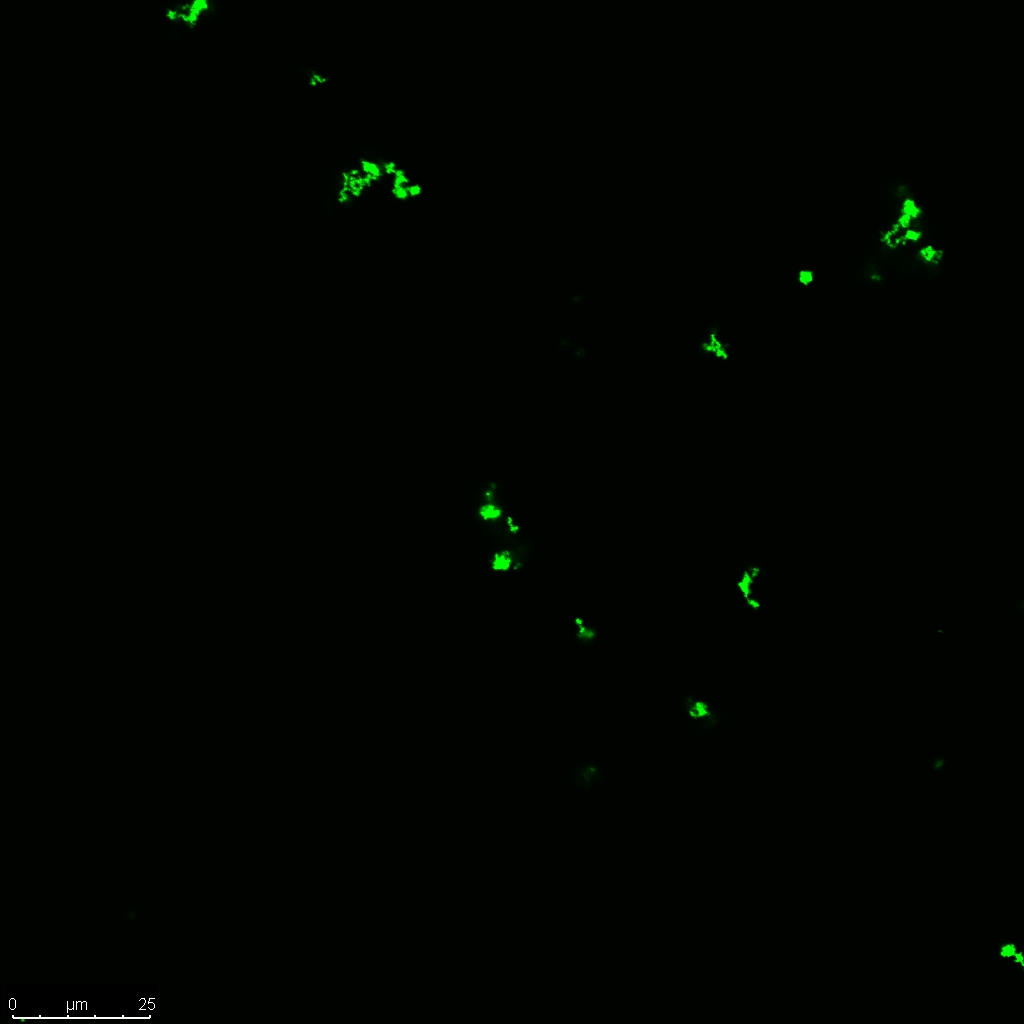

Supplement: Figure 2—source data 3. [file elife-94795-fig2-data3.zip › Figure 2B/replicate III/Figure 2B panel 3 Luc agg YSS replicate 3 photo 5.jpg]

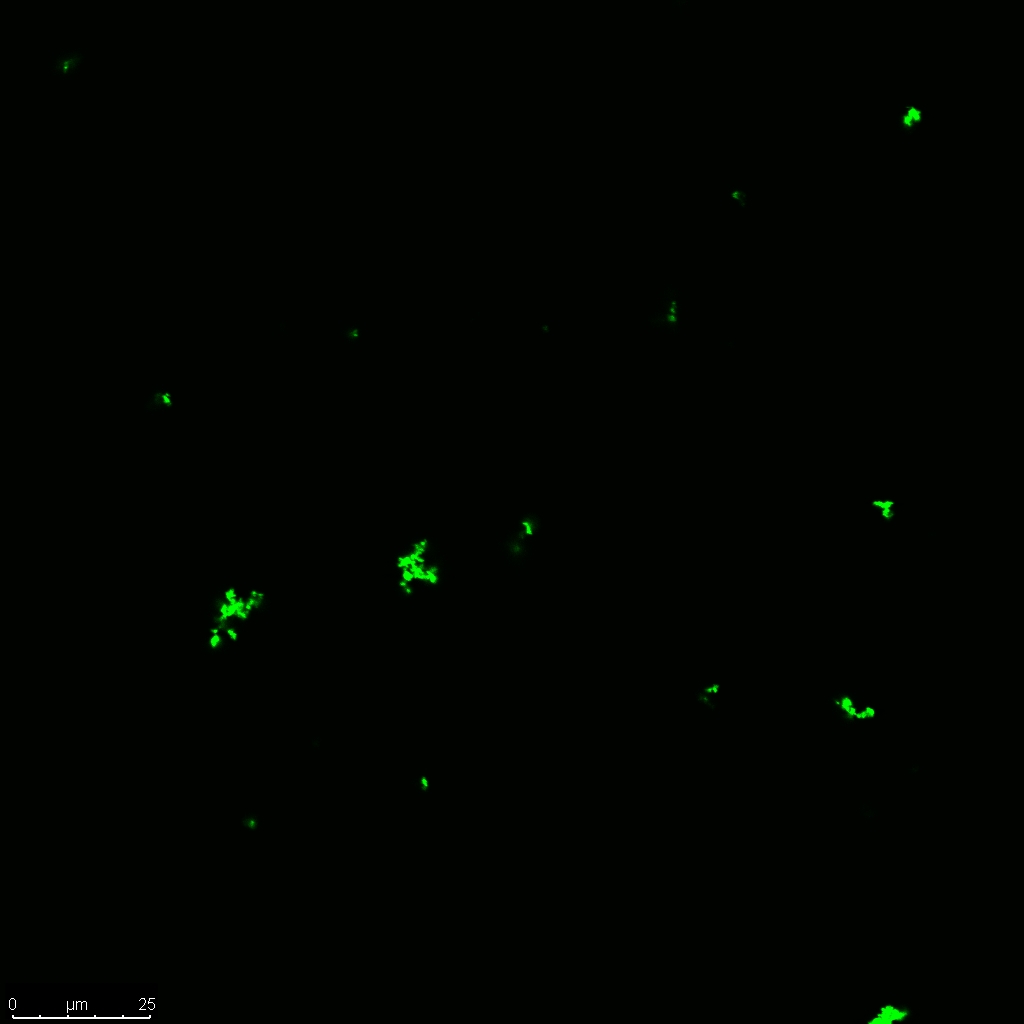

Supplement: Figure 2—source data 3. [file elife-94795-fig2-data3.zip › Figure 2B/replicate III/Figure 2B panel 3 Luc agg YSS replicate 3 photo 4.jpg]

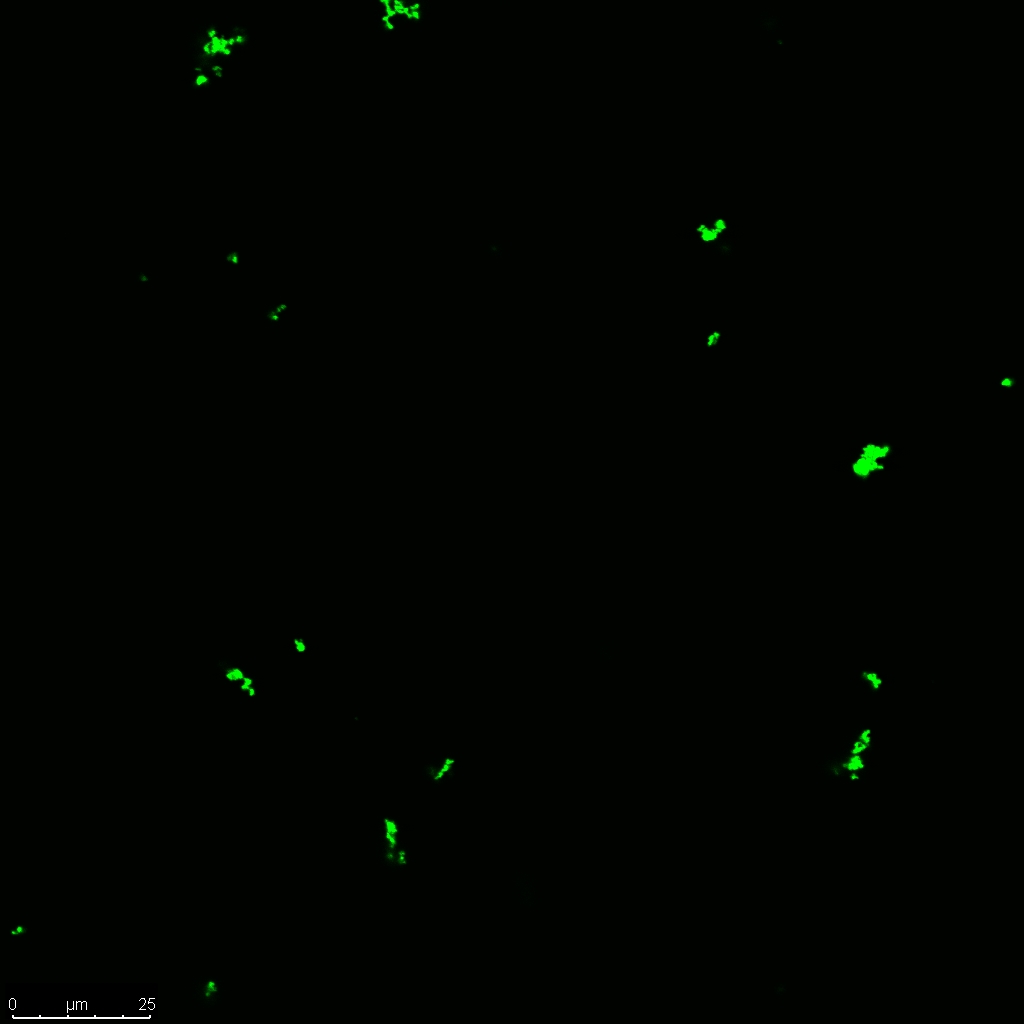

Supplement: Figure 2—source data 3. [file elife-94795-fig2-data3.zip › Figure 2B/replicate III/Figure 2B panel 3 Luc agg YSS replicate 3 photo 3.jpg]

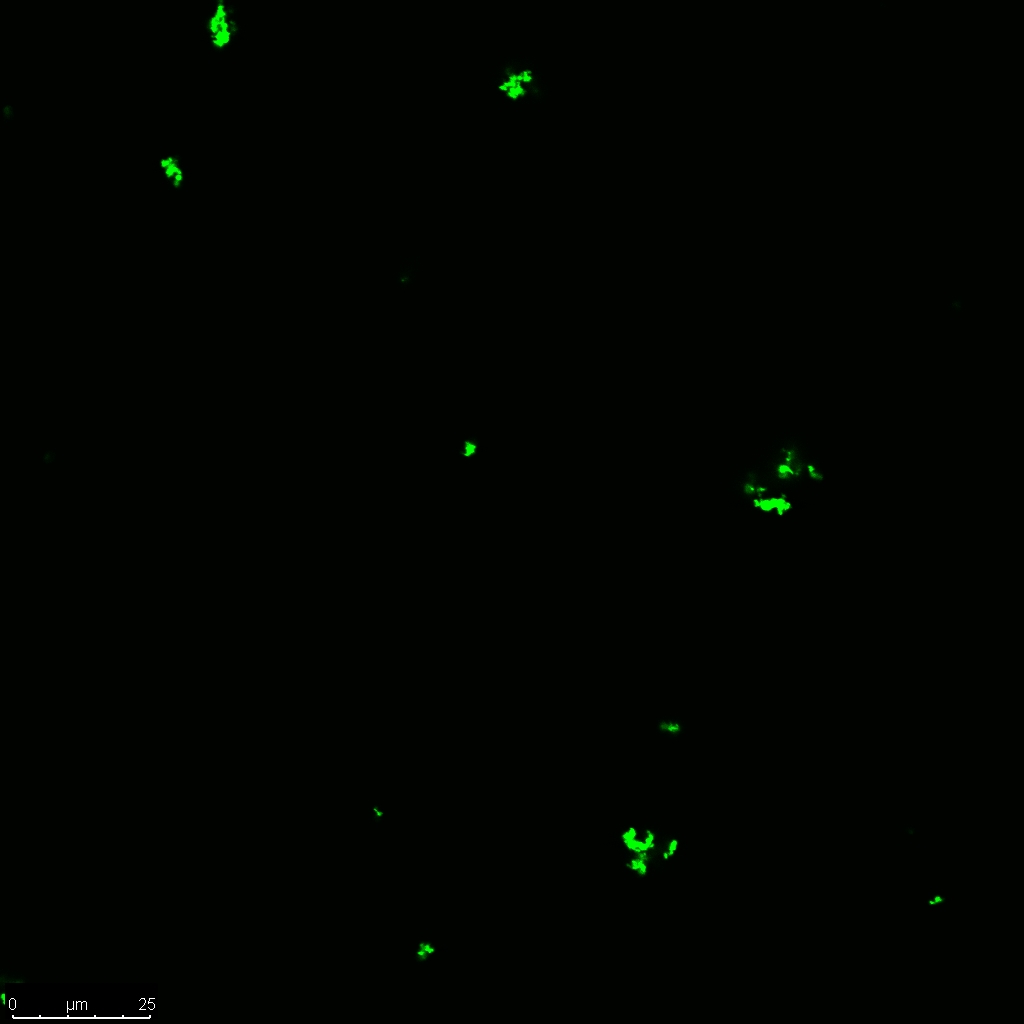

Supplement: Figure 2—source data 3. [file elife-94795-fig2-data3.zip › Figure 2B/replicate III/Figure 2B panel 3 Luc agg YSS replicate 3 photo 2.jpg]

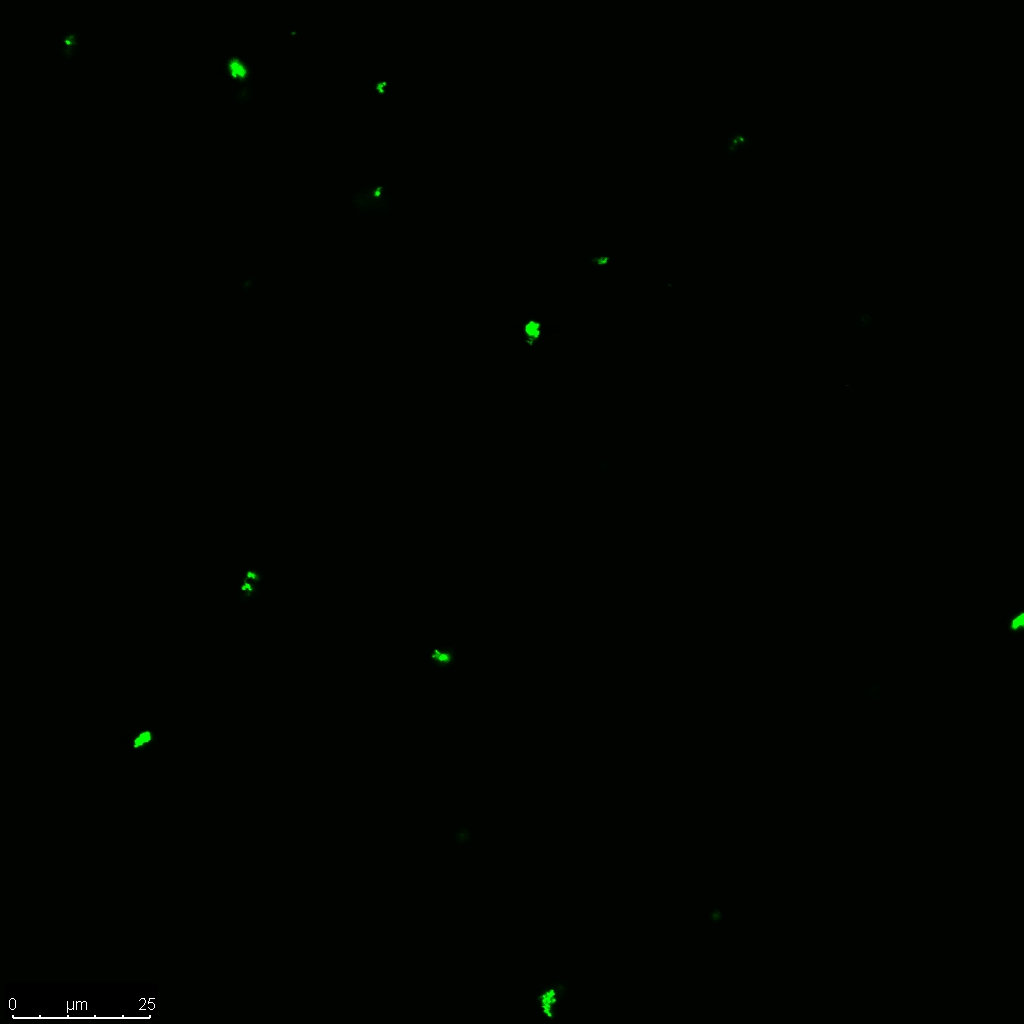

Supplement: Figure 2—source data 3. [file elife-94795-fig2-data3.zip › Figure 2B/replicate III/Figure 2B panel 3 Luc agg YSS replicate 3 photo 1.jpg]

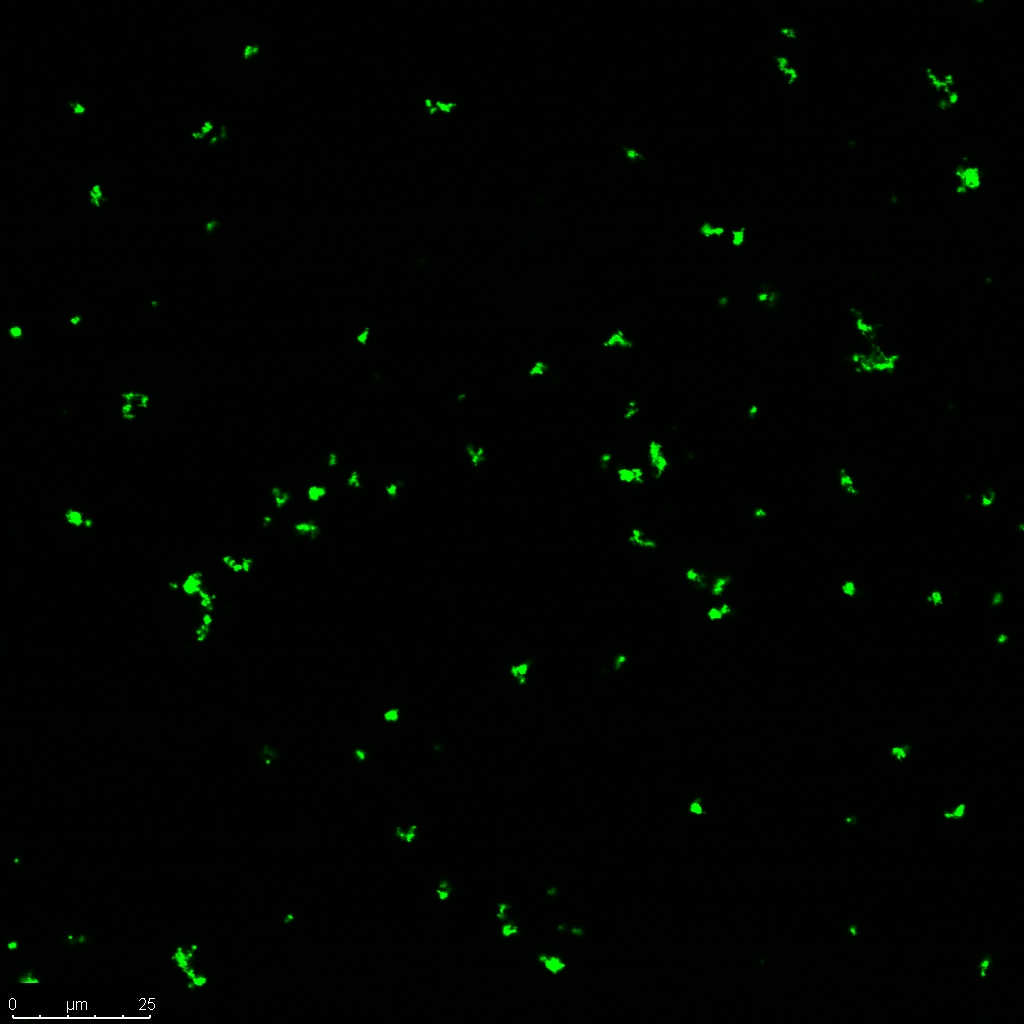

Supplement: Figure 2—source data 3. [file elife-94795-fig2-data3.zip › Figure 2B/replicate III/Figure 2B panel 1 Luc agg replicate 3 photo 1.jpg]

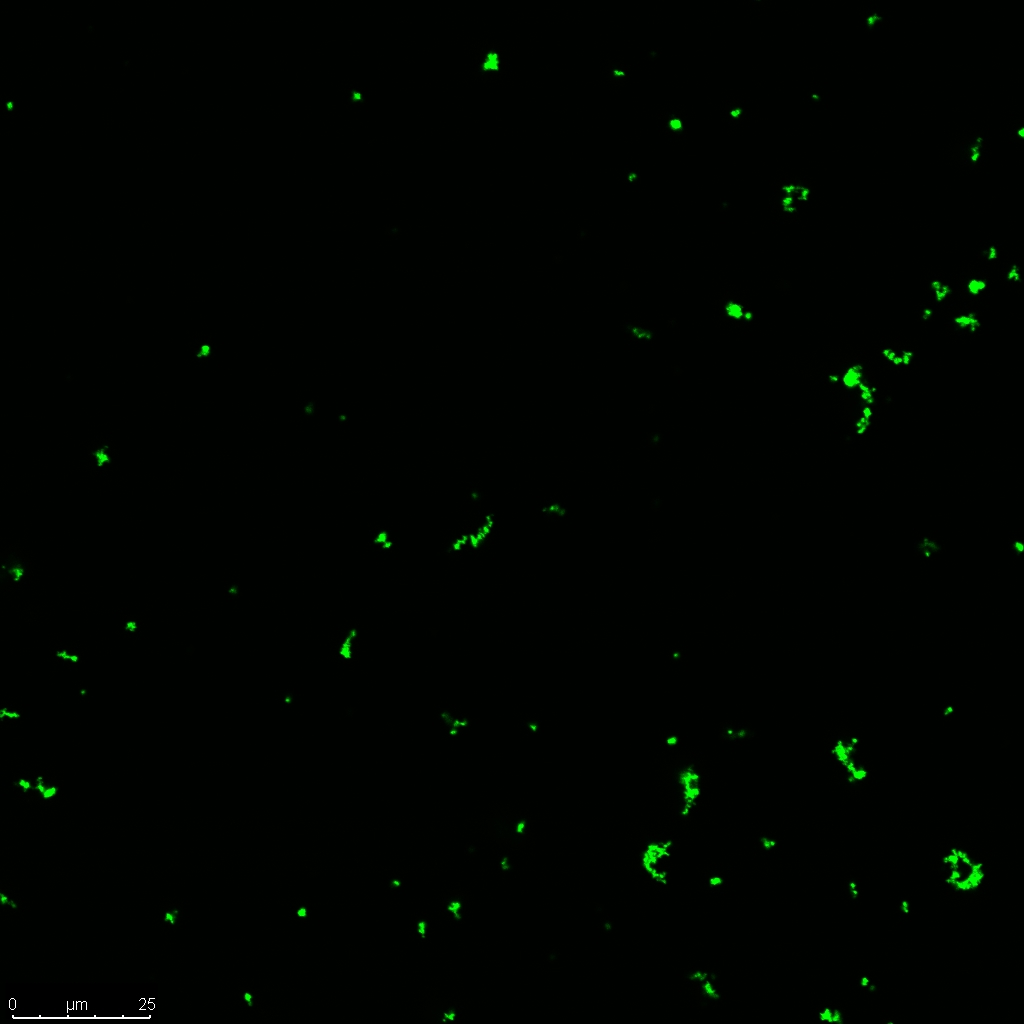

Supplement: Figure 2—source data 3. [file elife-94795-fig2-data3.zip › Figure 2B/replicate III/Figure 2B panel 1 Luc agg replicate 3 photo 2.jpg]

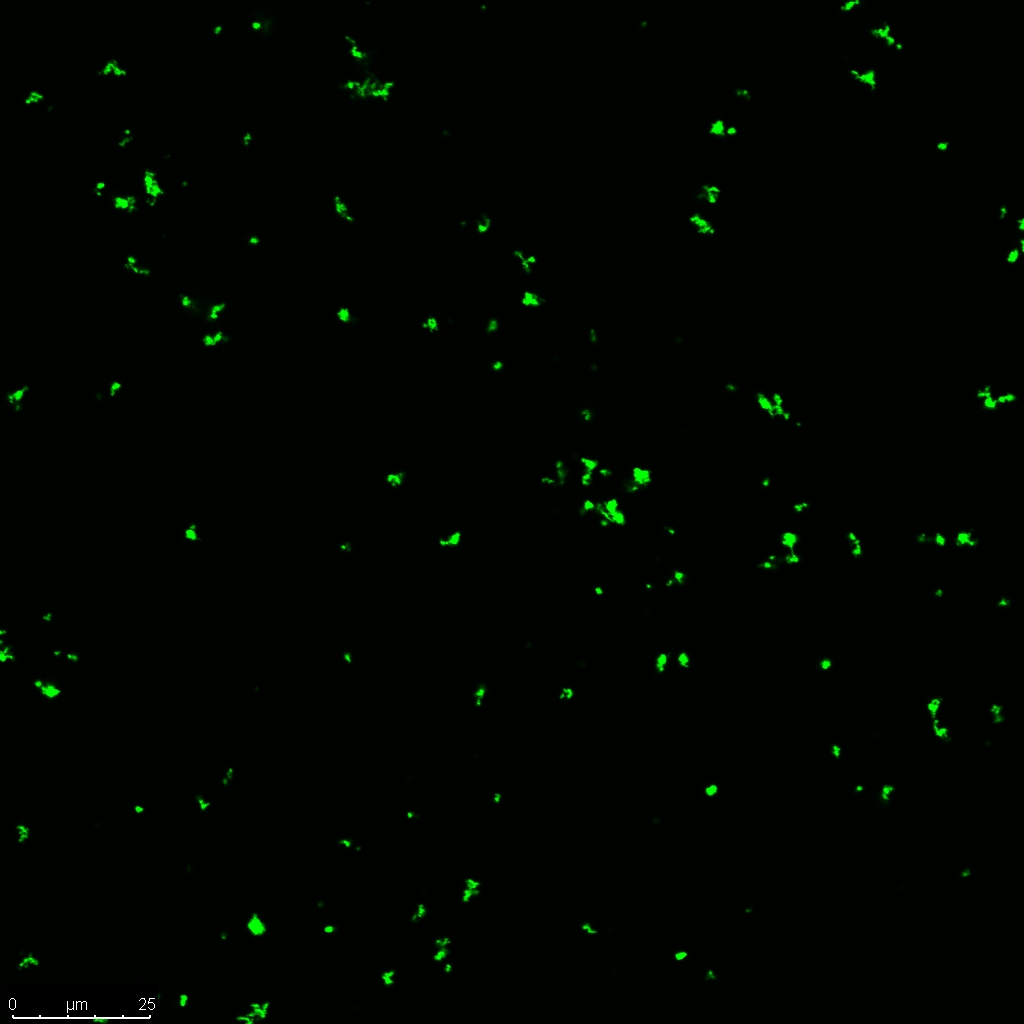

Supplement: Figure 2—source data 3. [file elife-94795-fig2-data3.zip › Figure 2B/replicate III/Figure 2B panel 1 Luc agg replicate 3 photo 3.jpg]

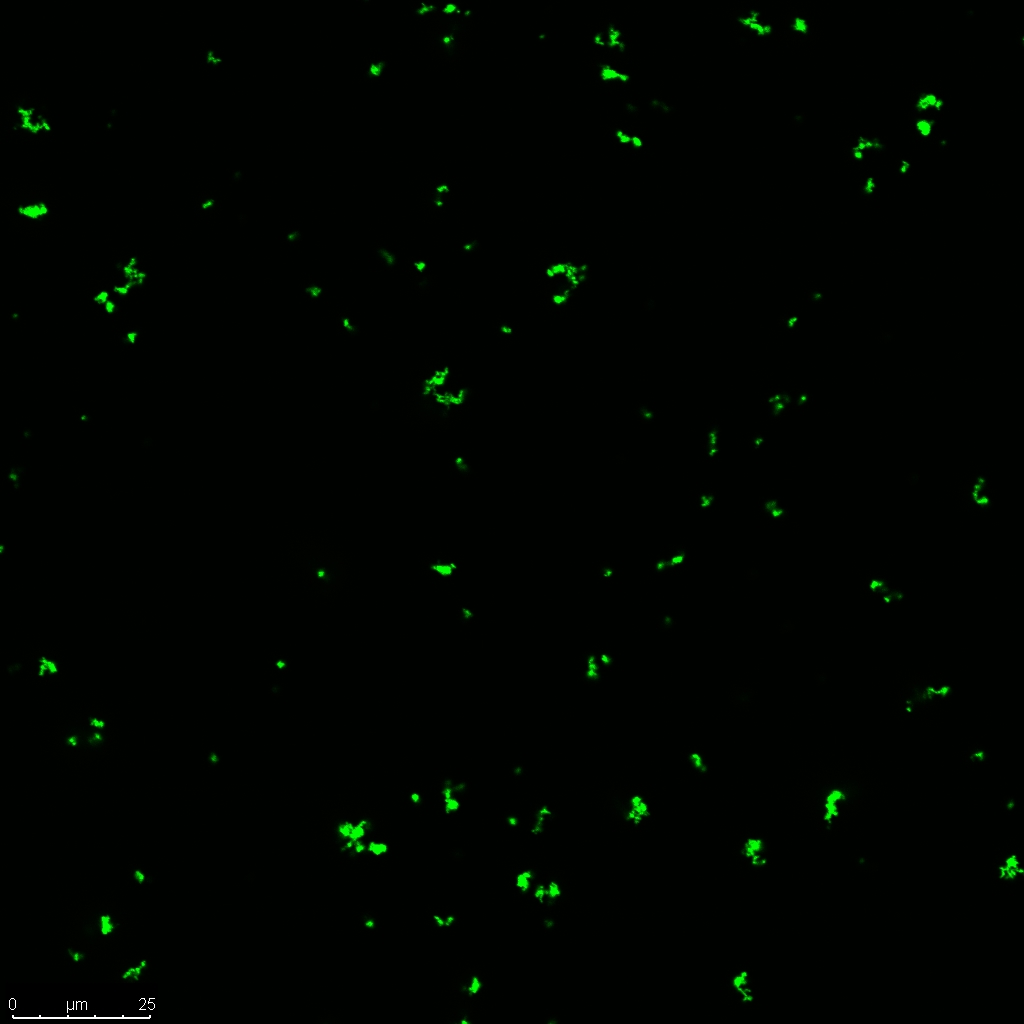

Supplement: Figure 2—source data 3. [file elife-94795-fig2-data3.zip › Figure 2B/replicate III/Figure 2B panel 1 Luc agg replicate 3 photo 4.jpg]

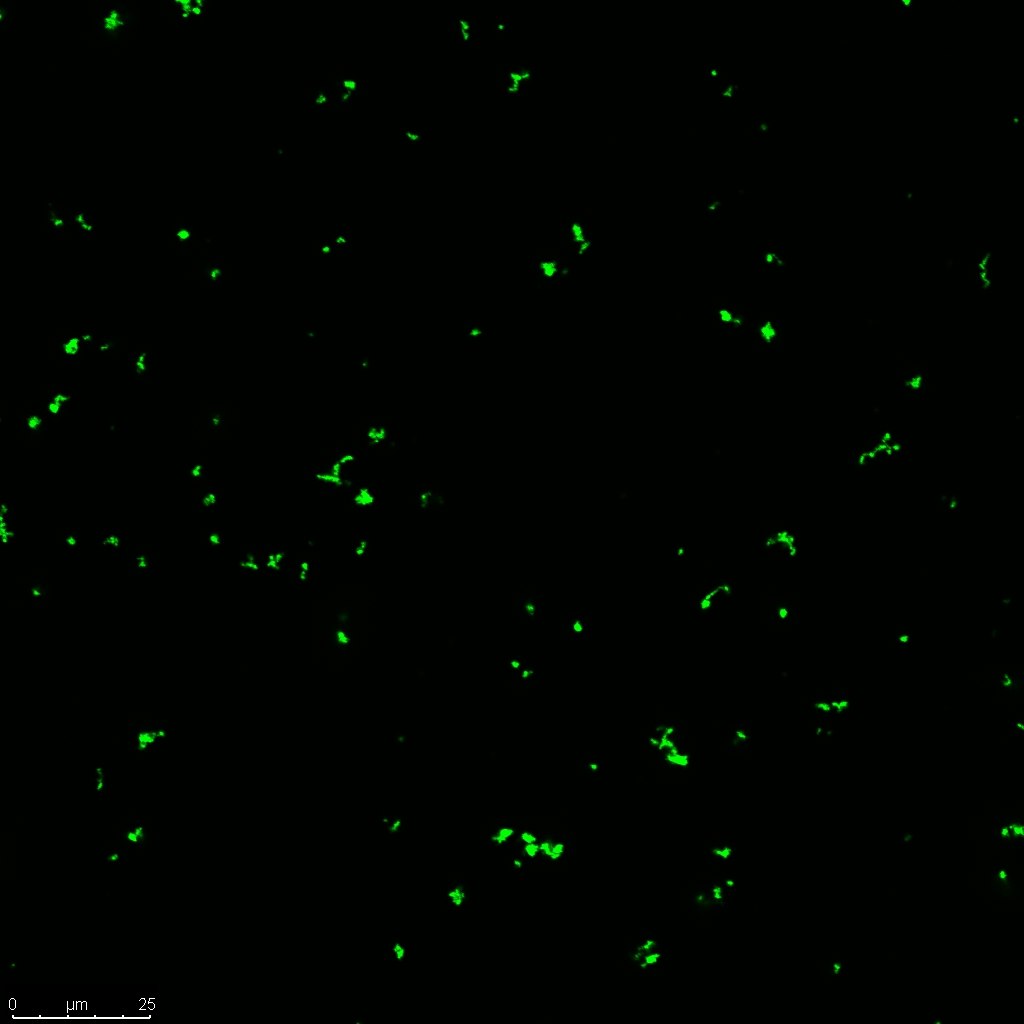

Supplement: Figure 2—source data 3. [file elife-94795-fig2-data3.zip › Figure 2B/replicate III/Figure 2B panel 1 Luc agg replicate 3 photo 5.jpg]

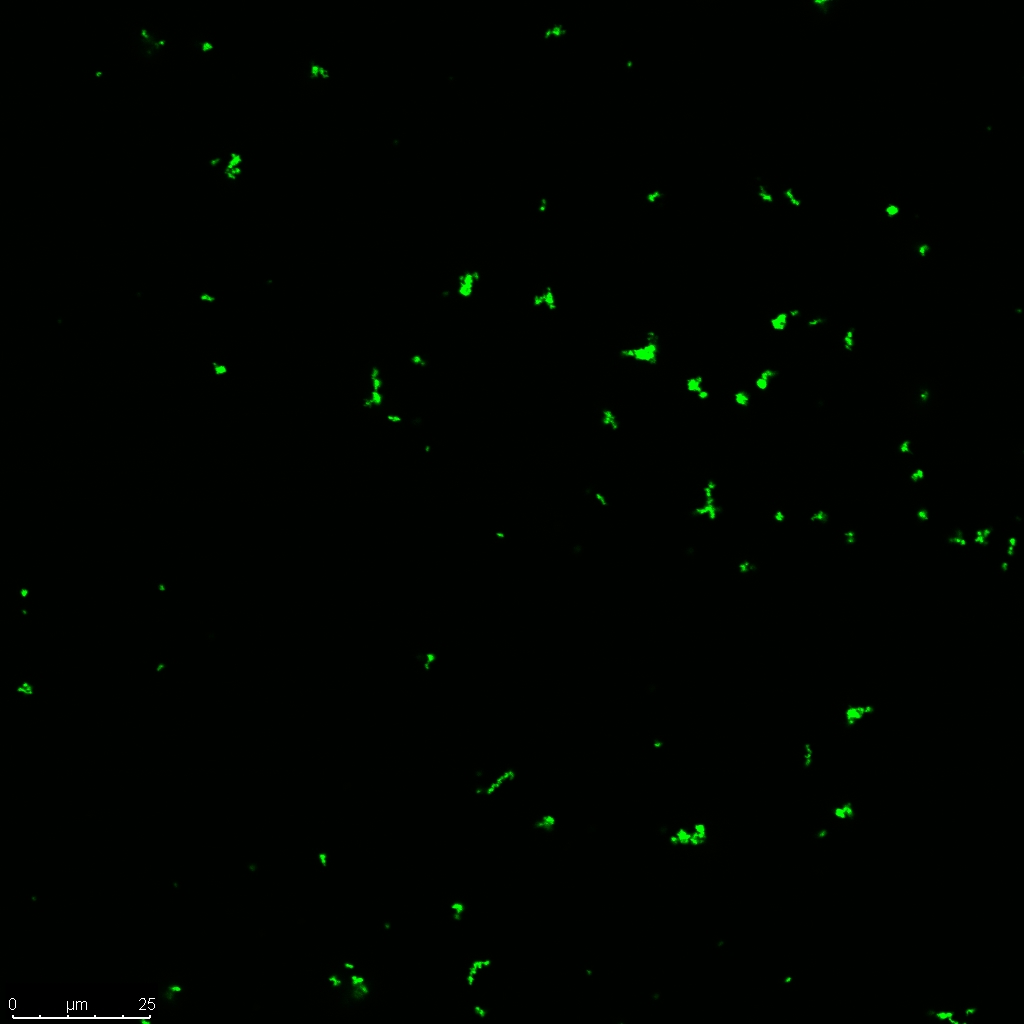

Supplement: Figure 2—source data 3. [file elife-94795-fig2-data3.zip › Figure 2B/replicate III/Figure 2B panel 1 Luc agg replicate 3 photo 6.jpg]

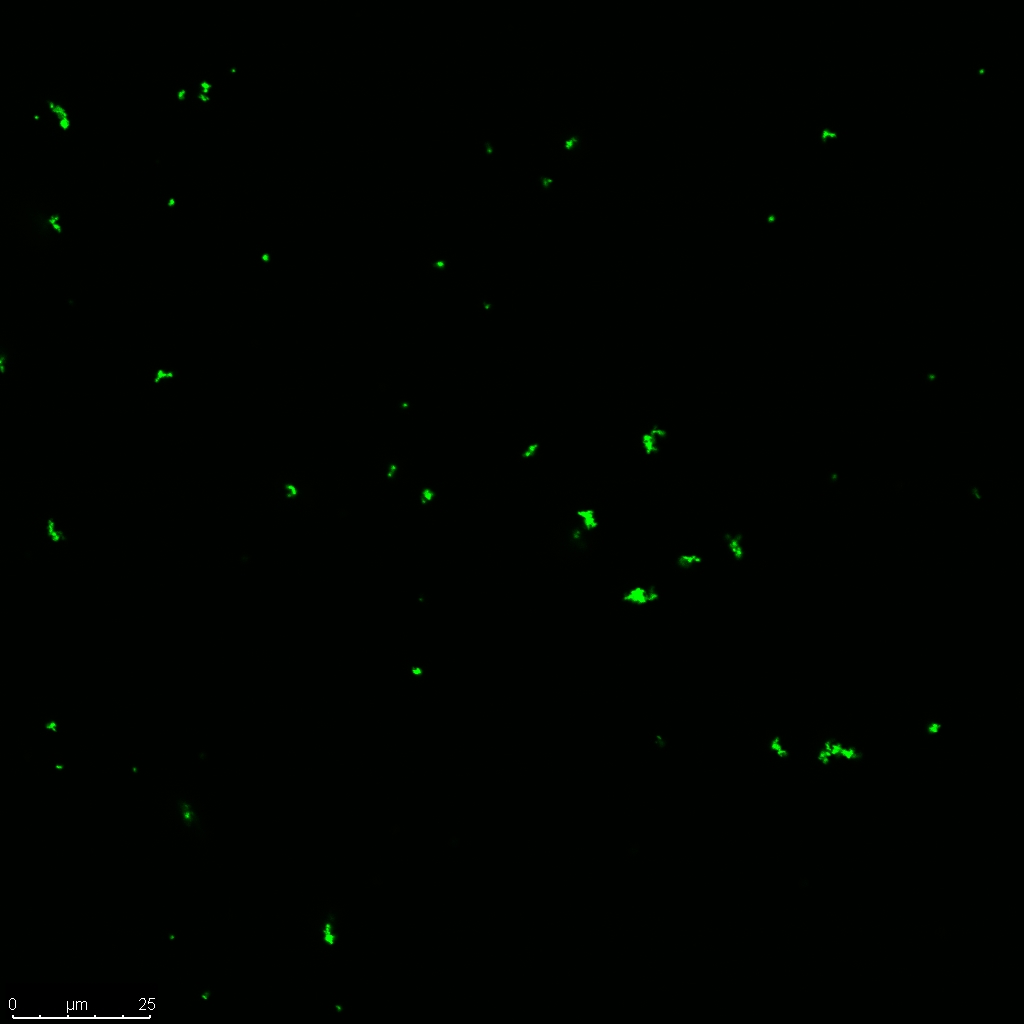

Supplement: Figure 2—source data 3. [file elife-94795-fig2-data3.zip › Figure 2B/replicate III/Figure 2B panel 1 Luc agg replicate 3 photo 7.jpg]

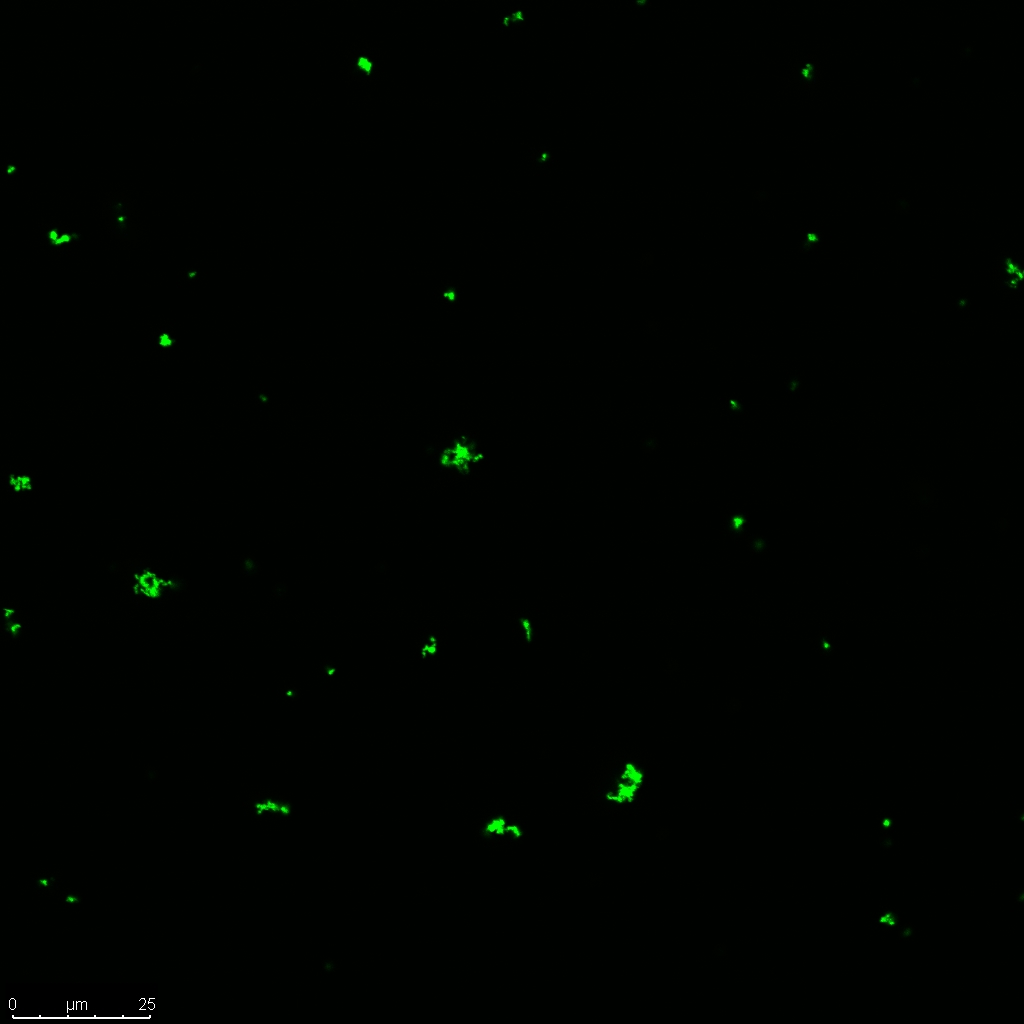

Supplement: Figure 2—source data 3. [file elife-94795-fig2-data3.zip › Figure 2B/replicate III/Figure 2B panel 1 Luc agg replicate 3 photo 8.jpg]

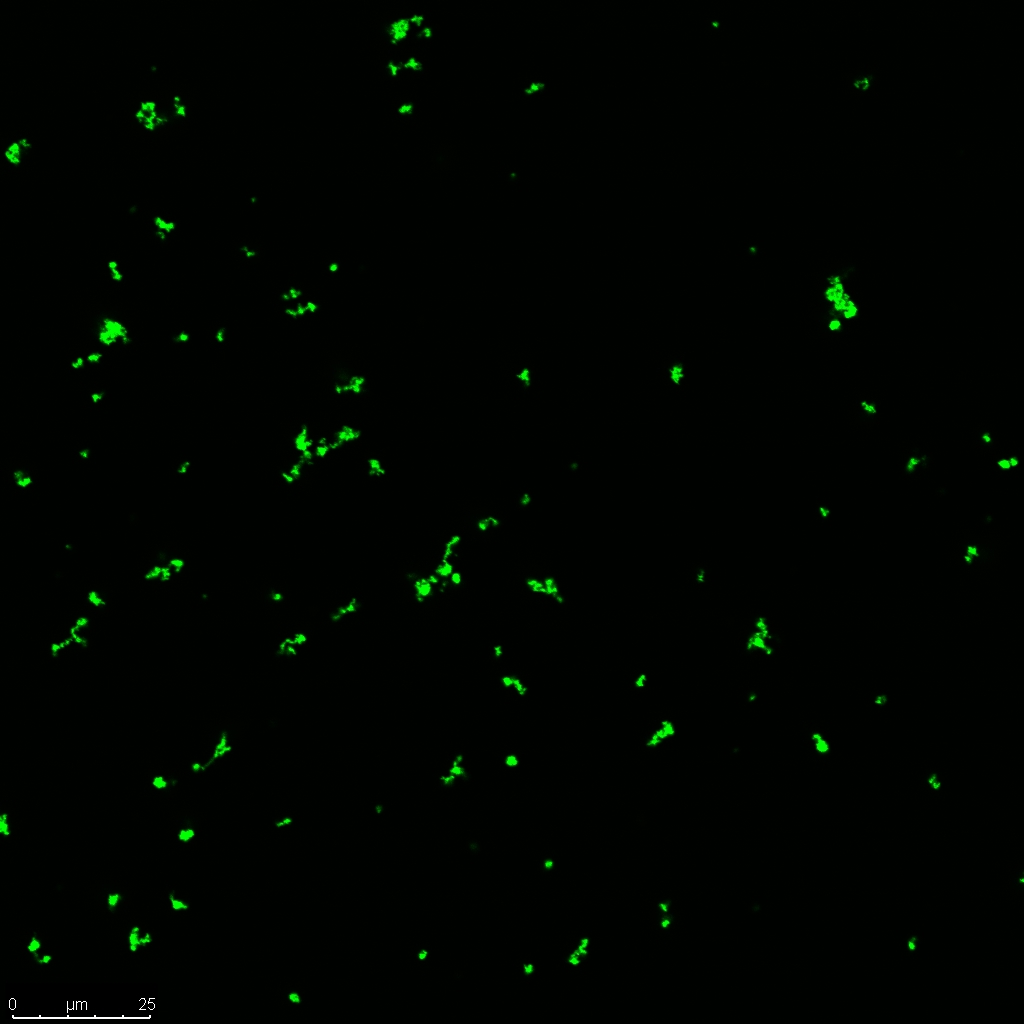

Supplement: Figure 2—source data 3. [file elife-94795-fig2-data3.zip › Figure 2B/replicate III/Figure 2B panel 1 Luc agg replicate 3 photo 9.jpg]

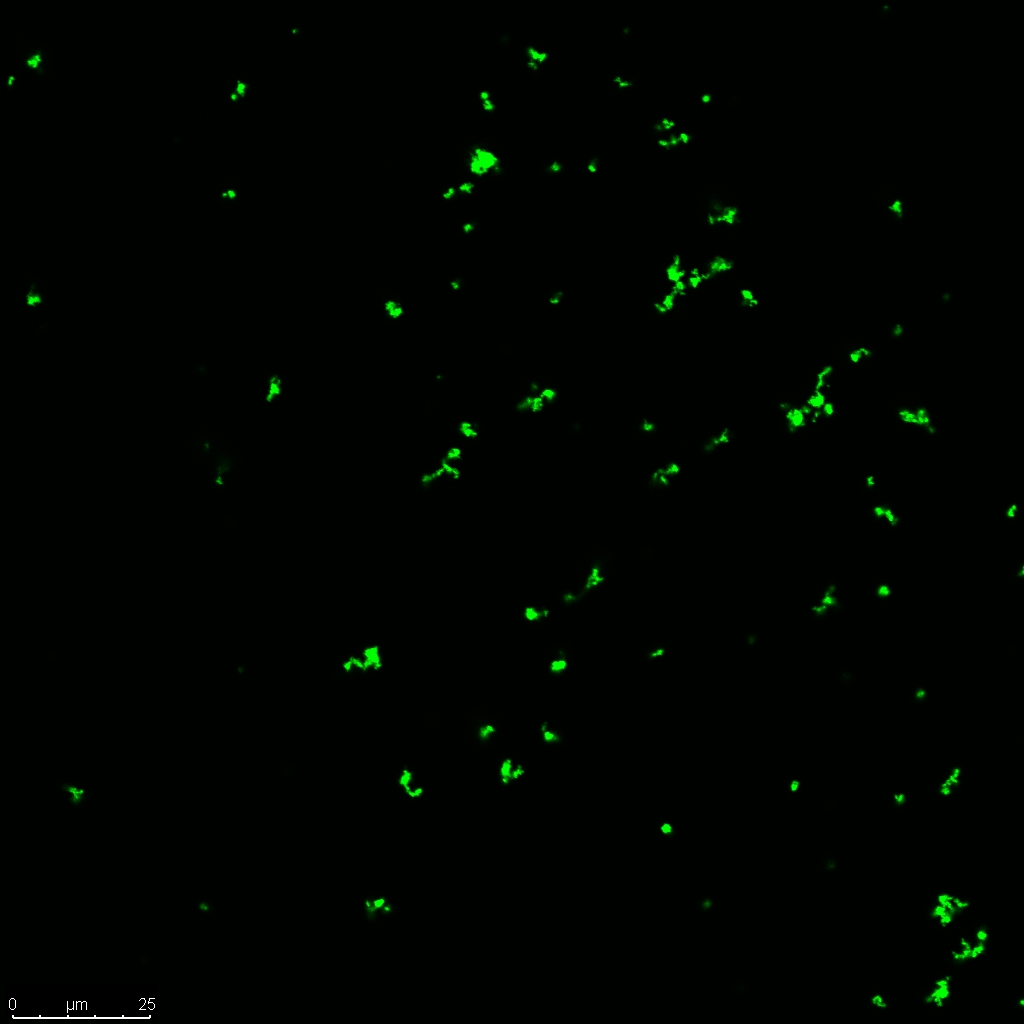

Supplement: Figure 2—source data 3. [file elife-94795-fig2-data3.zip › Figure 2B/replicate III/Figure 2B panel 1 Luc agg replicate 3 photo 10.jpg]

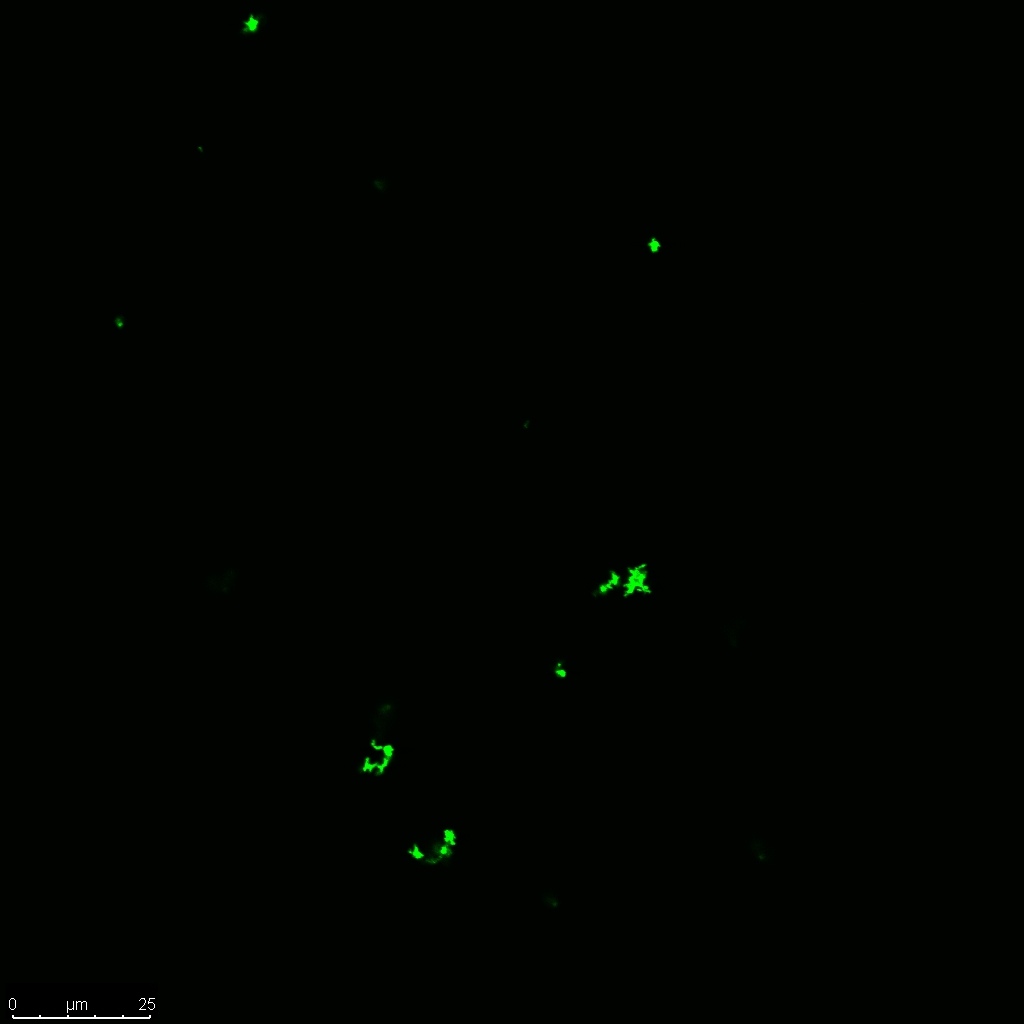

Supplement: Figure 2—source data 3. [file elife-94795-fig2-data3.zip › Figure 2B/replicate III/Figure 2B panel 4 Luc agg replicate 3 photo 1.jpg]

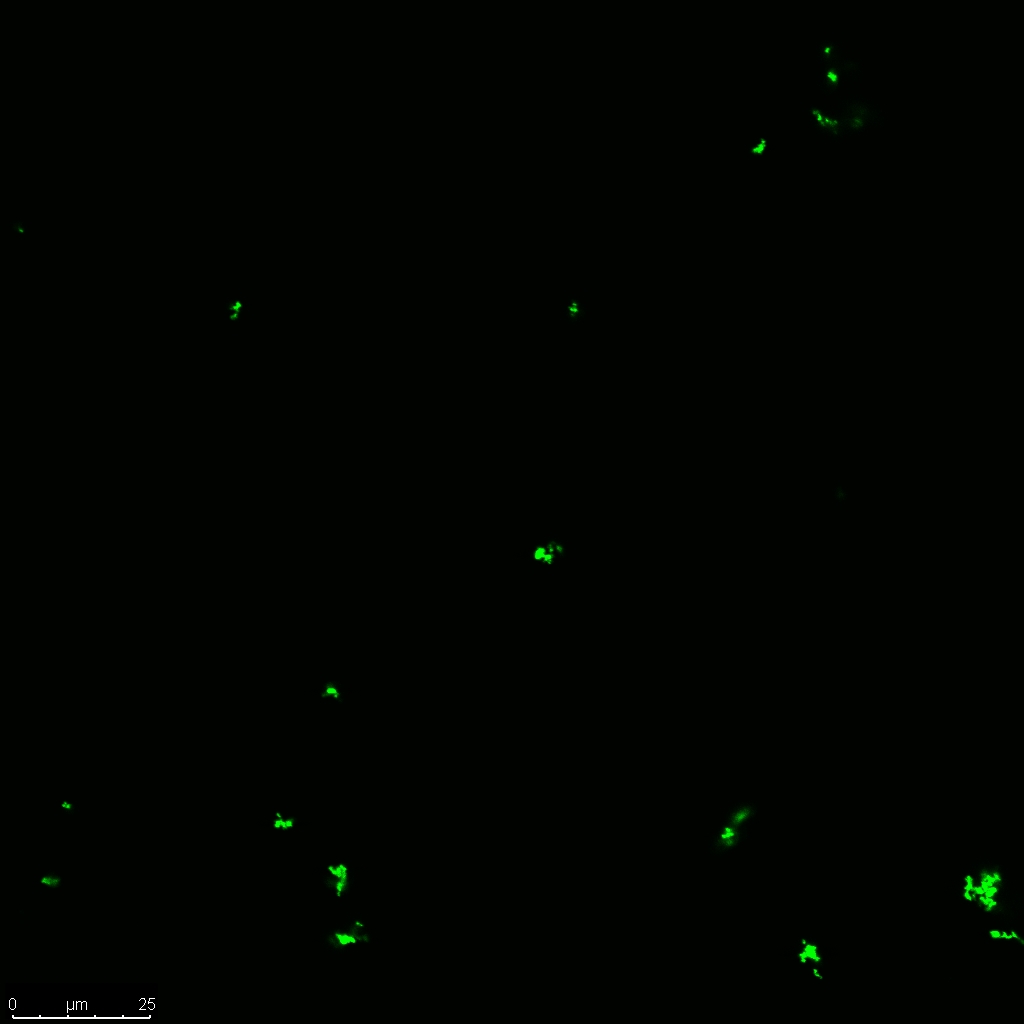

Supplement: Figure 2—source data 3. [file elife-94795-fig2-data3.zip › Figure 2B/replicate III/Figure 2B panel 4 Luc agg replicate 3 photo 2.jpg]

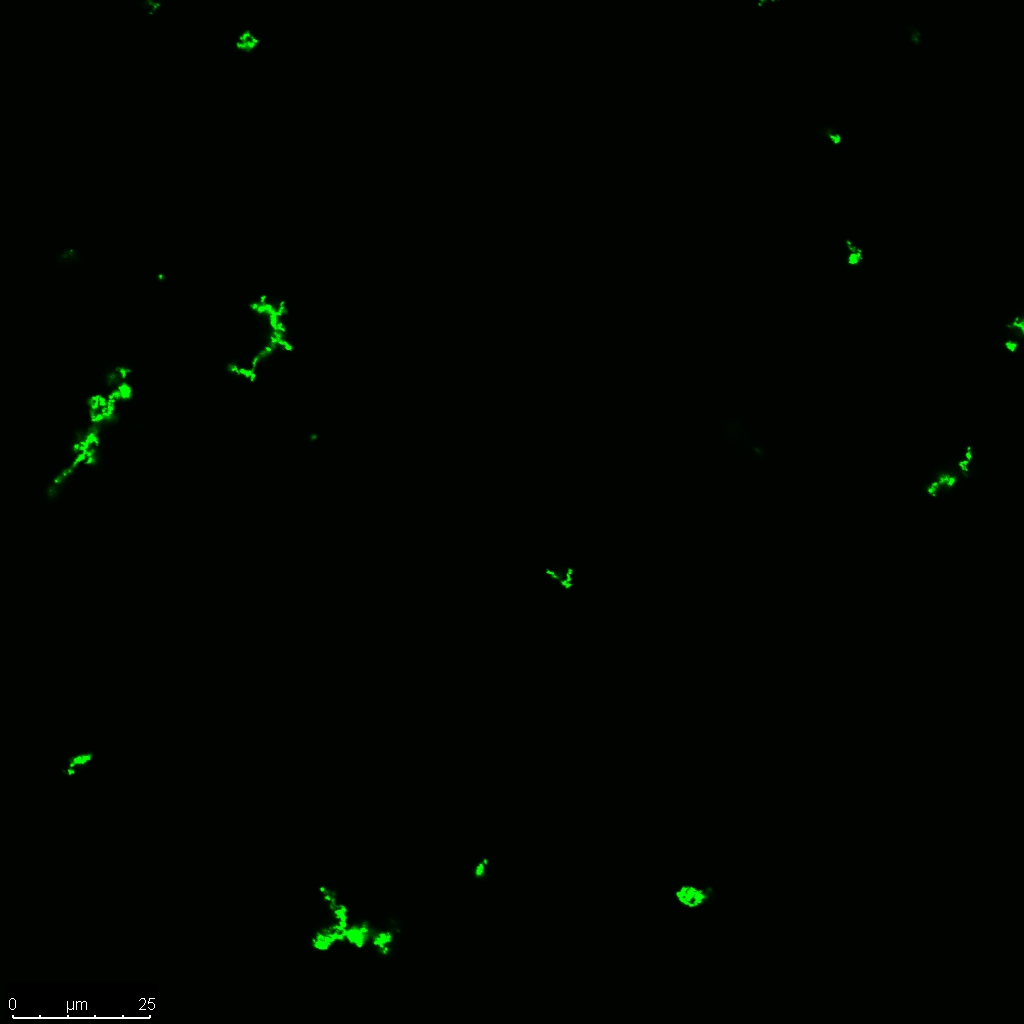

Supplement: Figure 2—source data 3. [file elife-94795-fig2-data3.zip › Figure 2B/replicate III/Figure 2B panel 4 Luc agg replicate 3 photo 3.jpg]

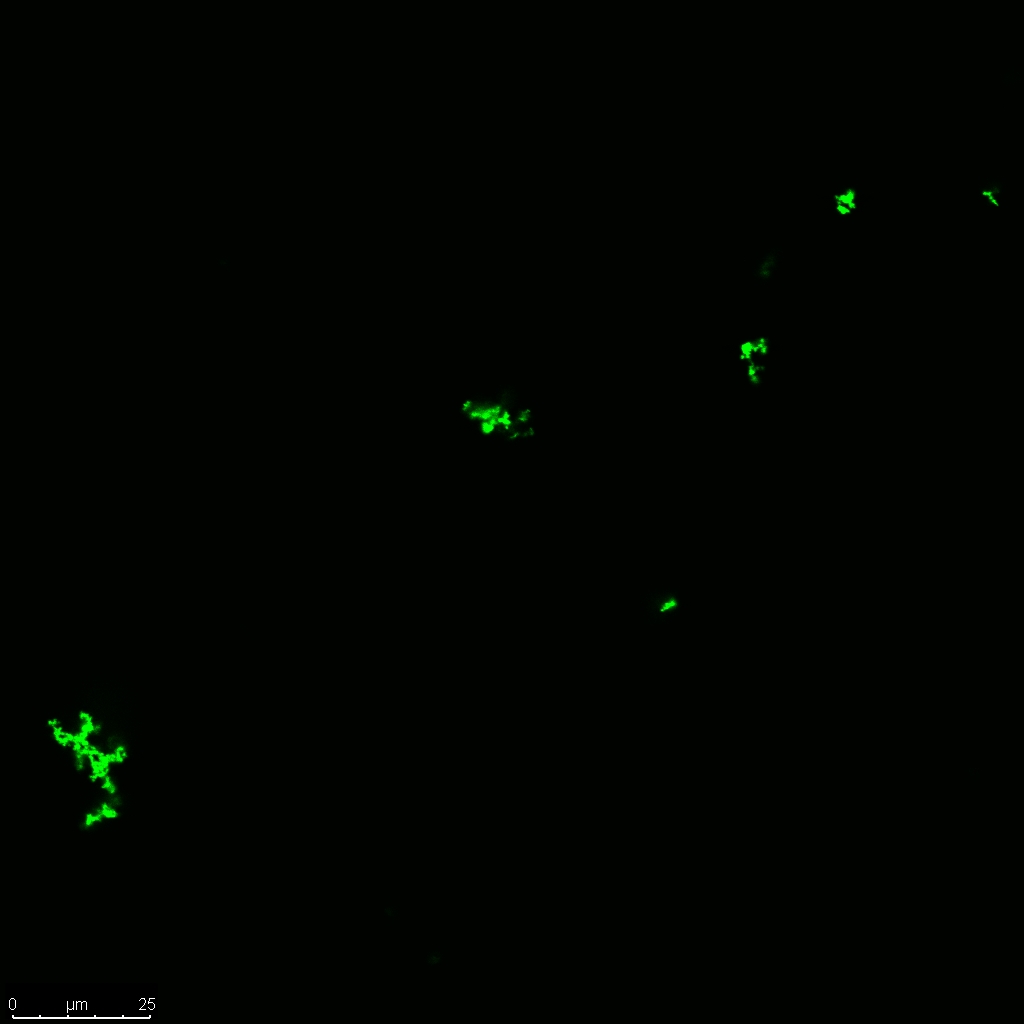

Supplement: Figure 2—source data 3. [file elife-94795-fig2-data3.zip › Figure 2B/replicate III/Figure 2B panel 4 Luc agg replicate 3 photo 4.jpg]

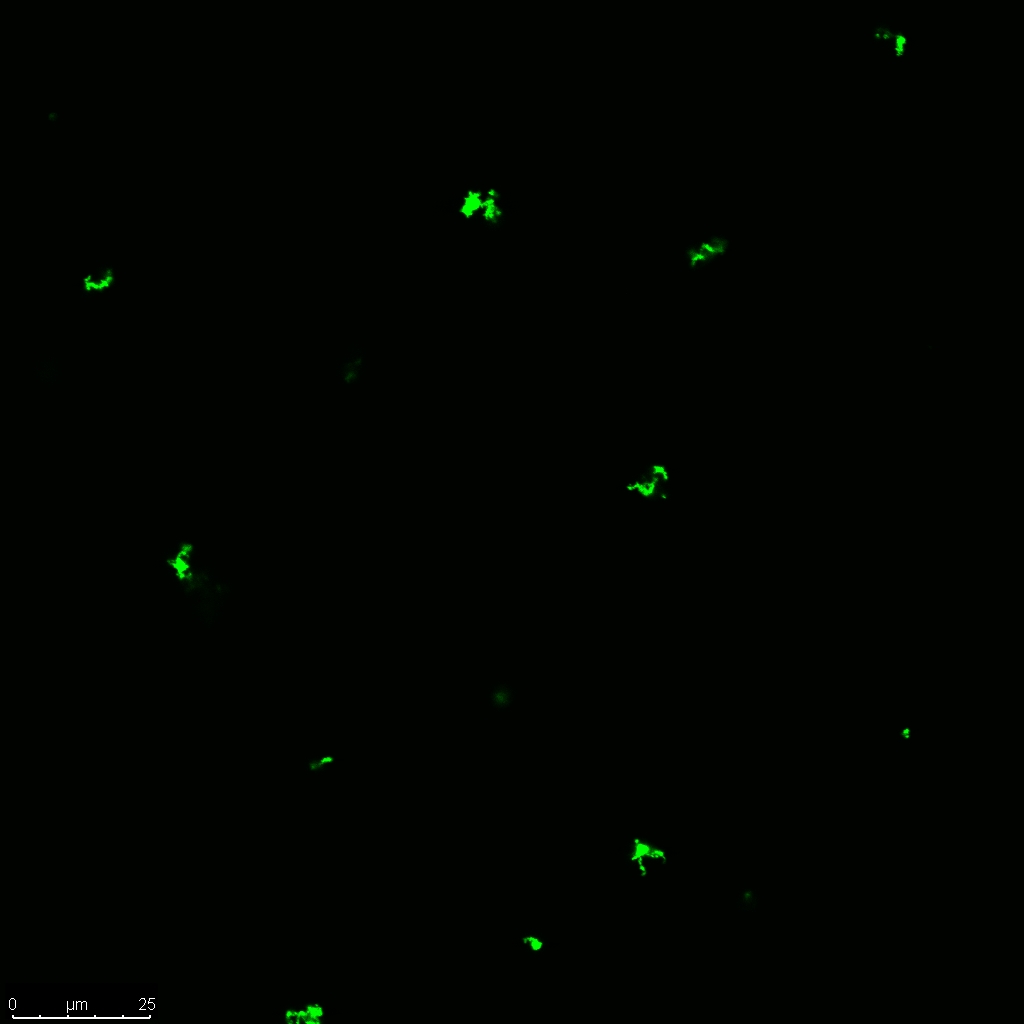

Supplement: Figure 2—source data 3. [file elife-94795-fig2-data3.zip › Figure 2B/replicate III/Figure 2B panel 4 Luc agg replicate 3 photo 5.jpg]

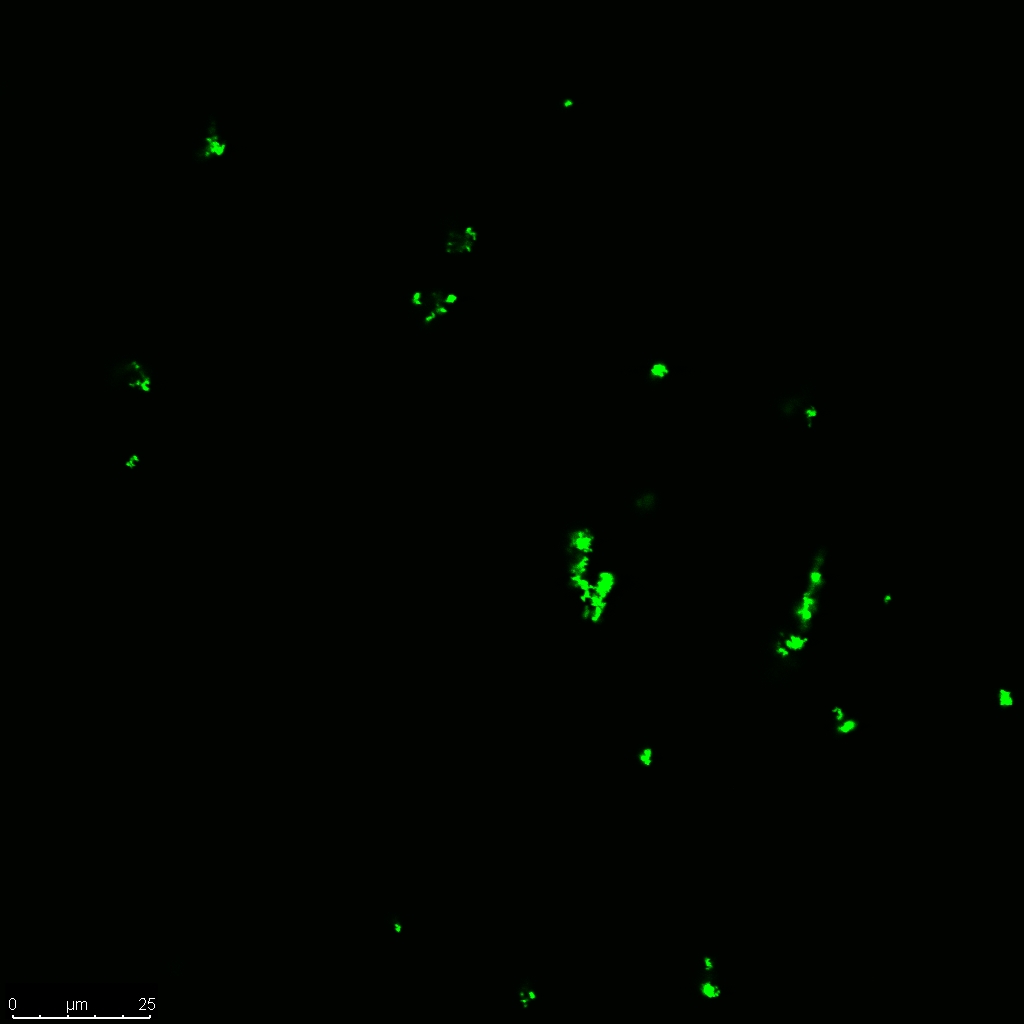

Supplement: Figure 2—source data 3. [file elife-94795-fig2-data3.zip › Figure 2B/replicate III/Figure 2B panel 4 Luc agg replicate 3 photo 6.jpg]

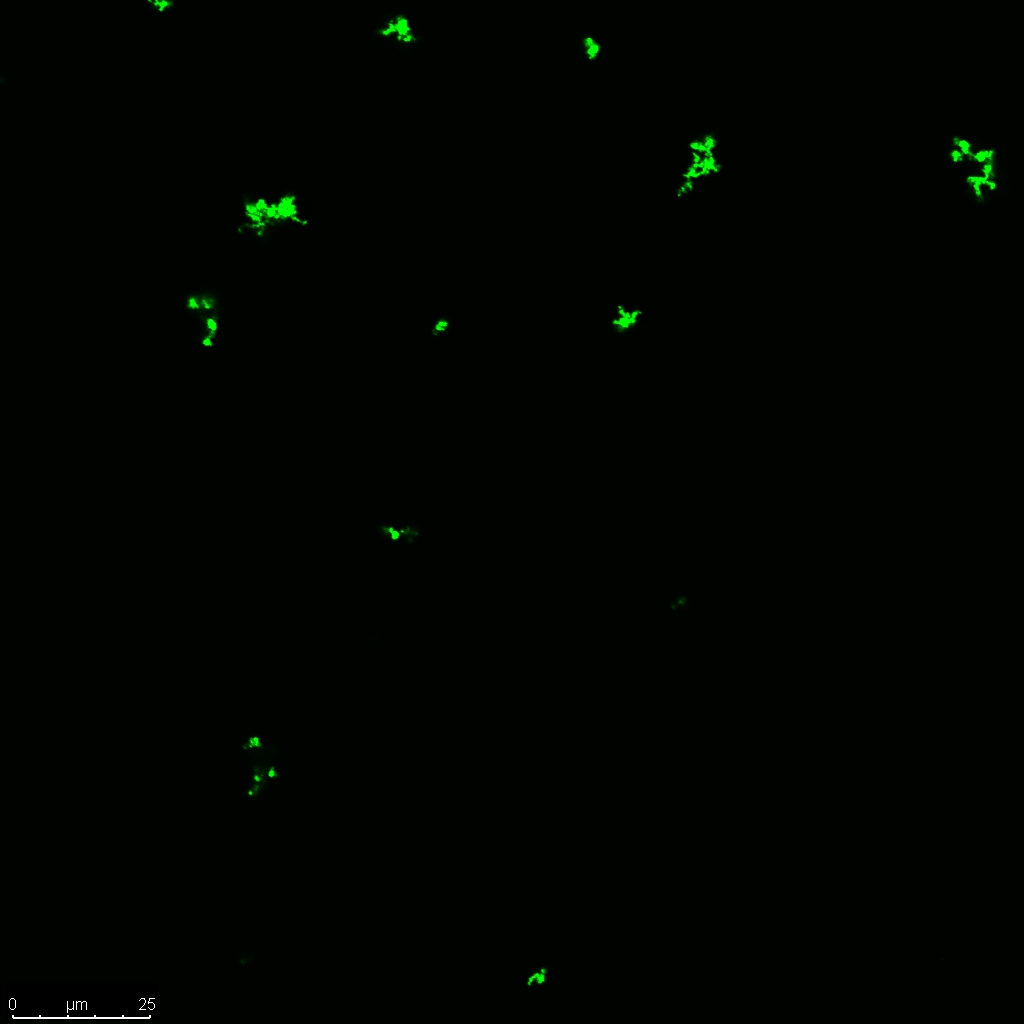

Supplement: Figure 2—source data 3. [file elife-94795-fig2-data3.zip › Figure 2B/replicate III/Figure 2B panel 4 Luc agg replicate 3 photo 7.jpg]

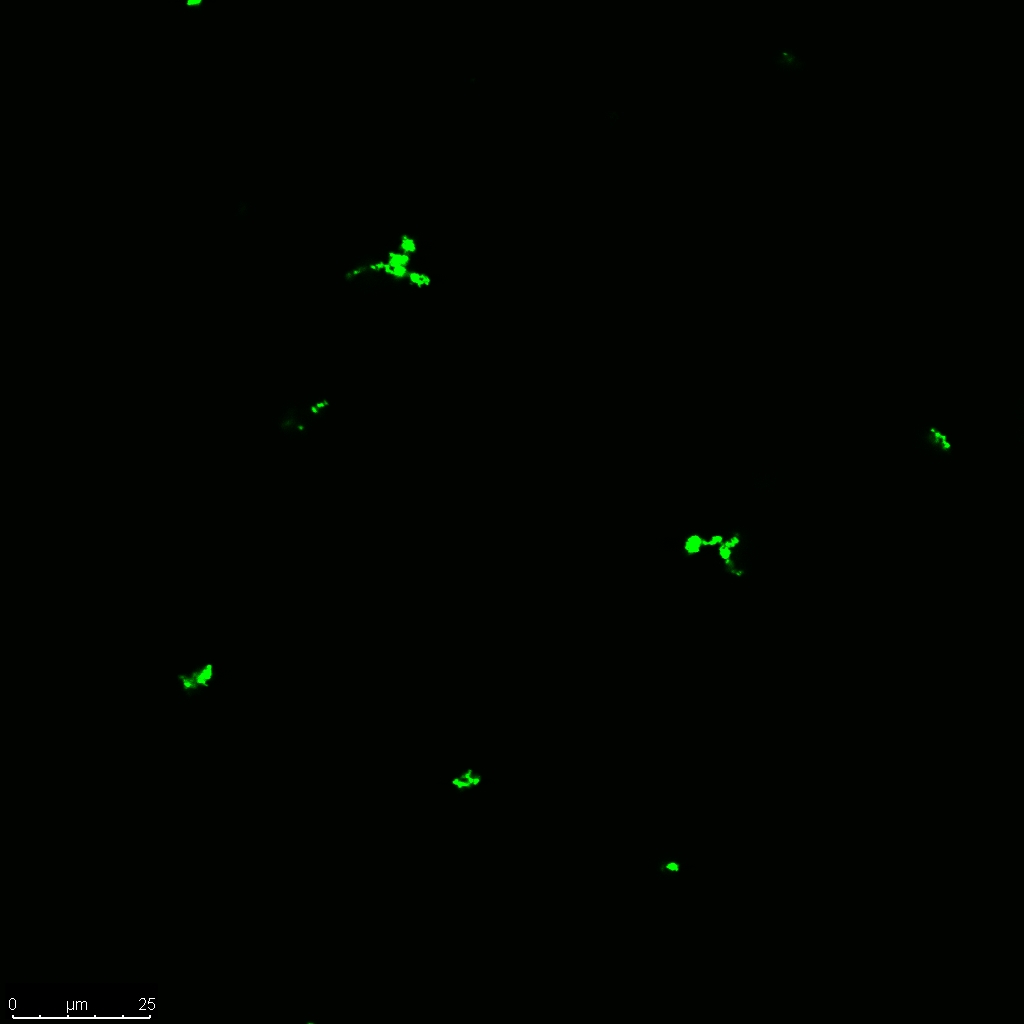

Supplement: Figure 2—source data 3. [file elife-94795-fig2-data3.zip › Figure 2B/replicate III/Figure 2B panel 4 Luc agg replicate 3 photo 8.jpg]

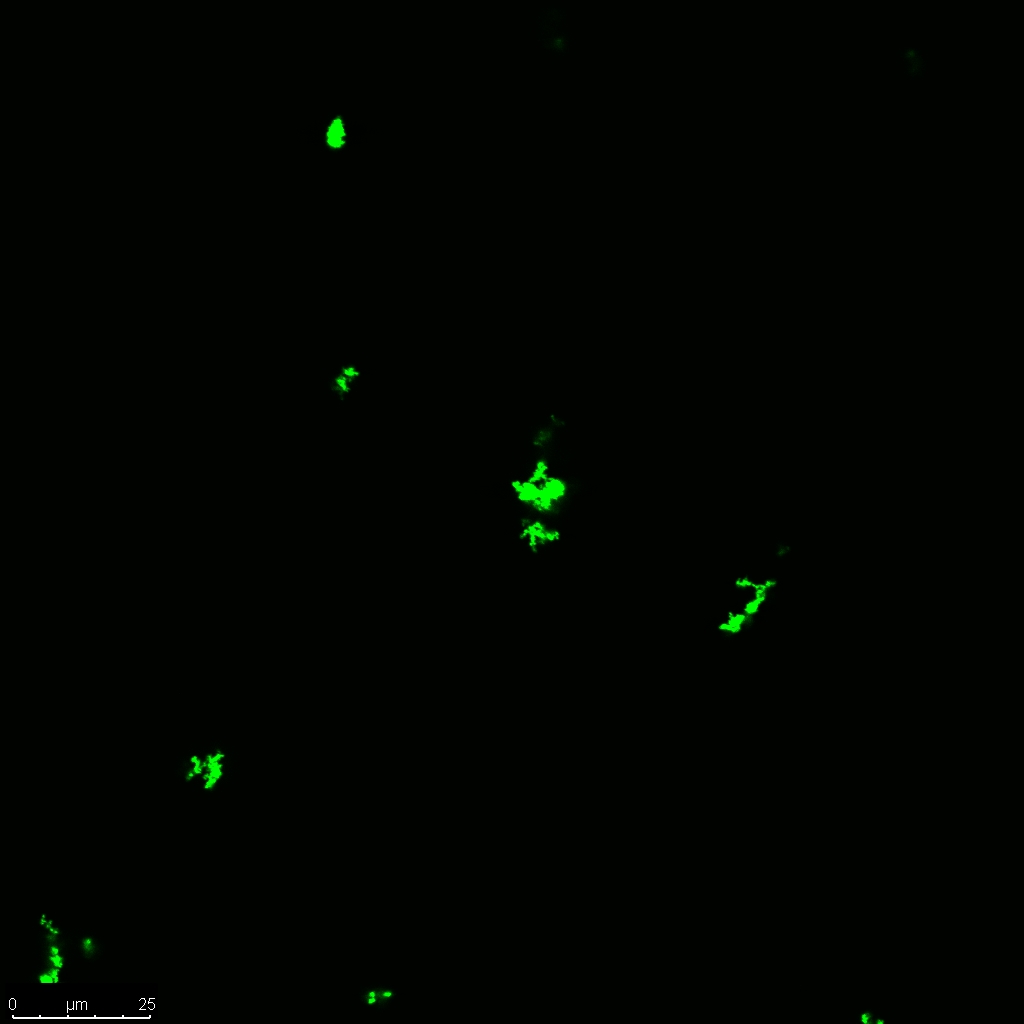

Supplement: Figure 2—source data 3. [file elife-94795-fig2-data3.zip › Figure 2B/replicate III/Figure 2B panel 4 Luc agg replicate 3 photo 9.jpg]

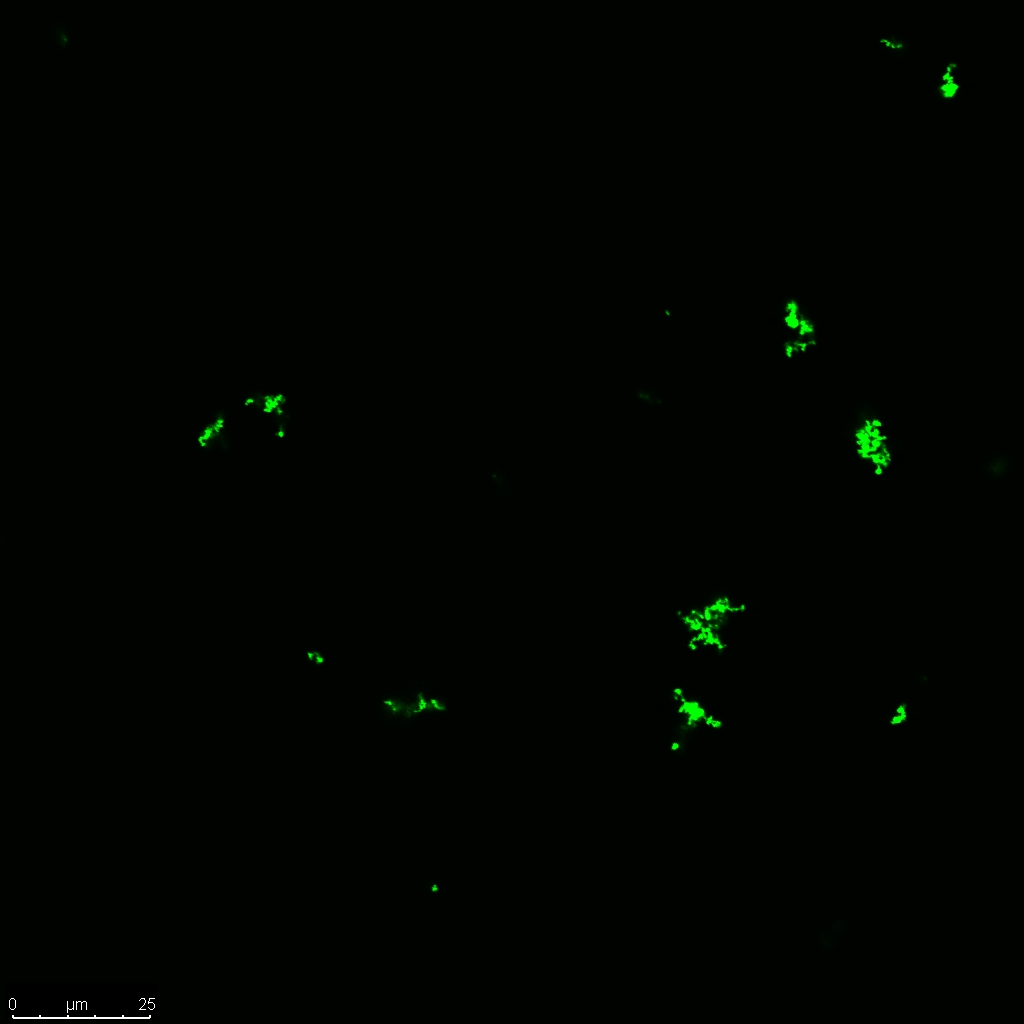

Supplement: Figure 2—source data 3. [file elife-94795-fig2-data3.zip › Figure 2B/replicate III/Figure 2B panel 4 Luc agg replicate 3 photo 10.jpg]

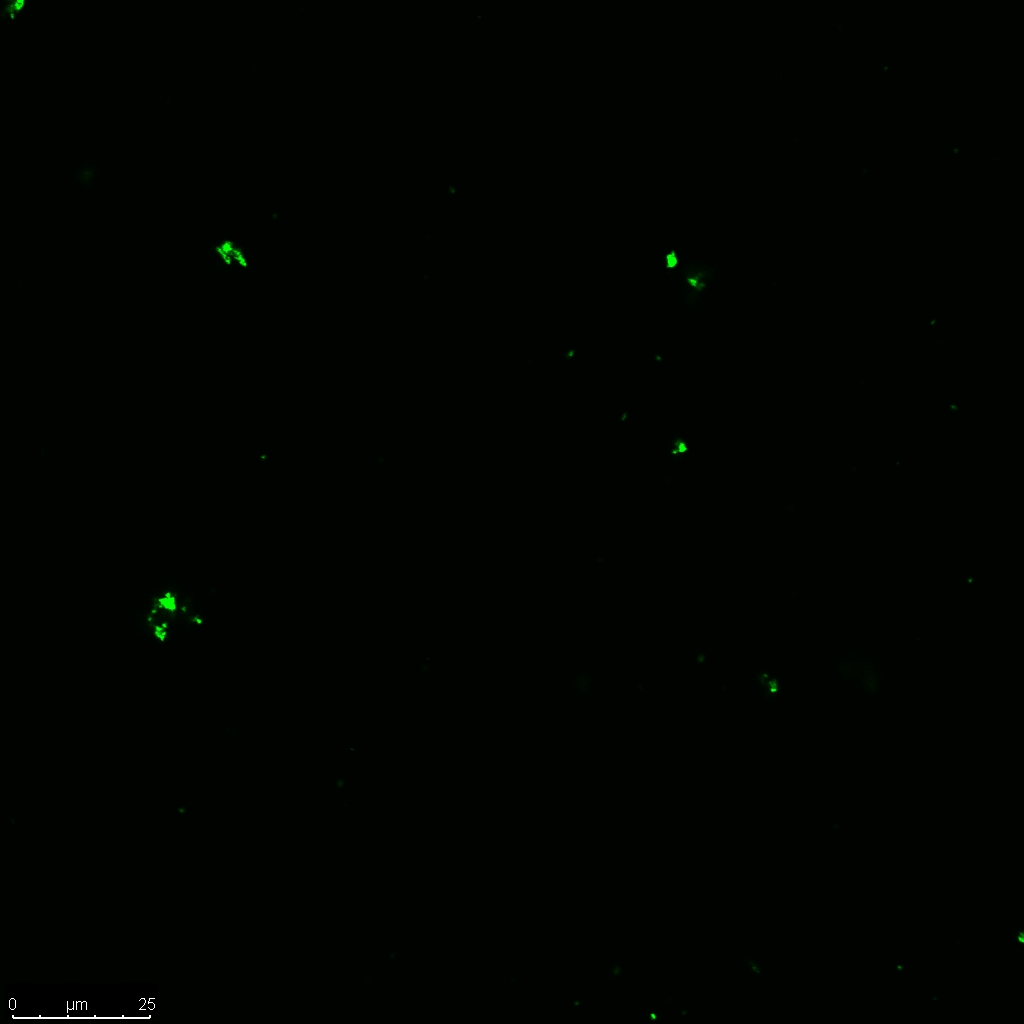

Supplement: Figure 2—source data 3. [file elife-94795-fig2-data3.zip › Figure 2B/replicate III/Figure 2B panel 6 Luc agg replicate 3 photo 1.jpg]

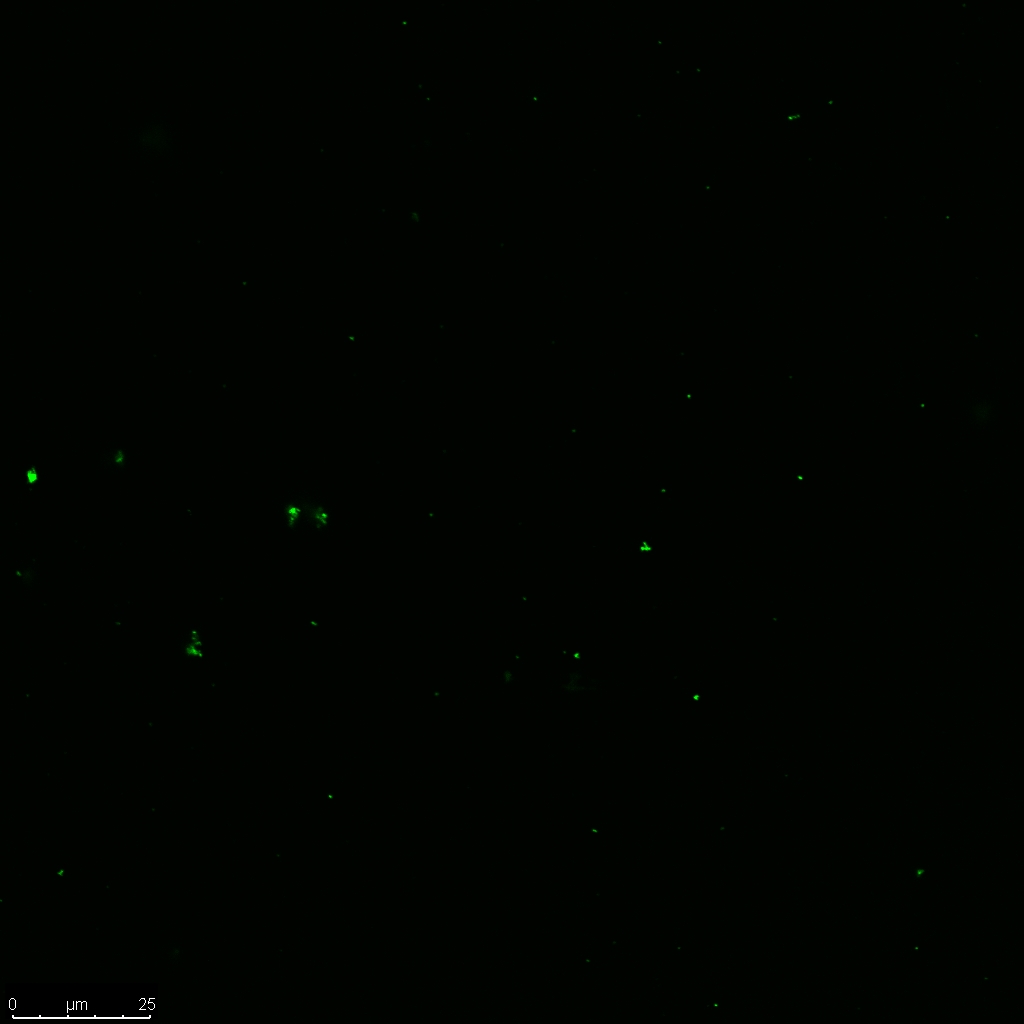

Supplement: Figure 2—source data 3. [file elife-94795-fig2-data3.zip › Figure 2B/replicate III/Figure 2B panel 6 Luc agg replicate 3 photo 2.jpg]

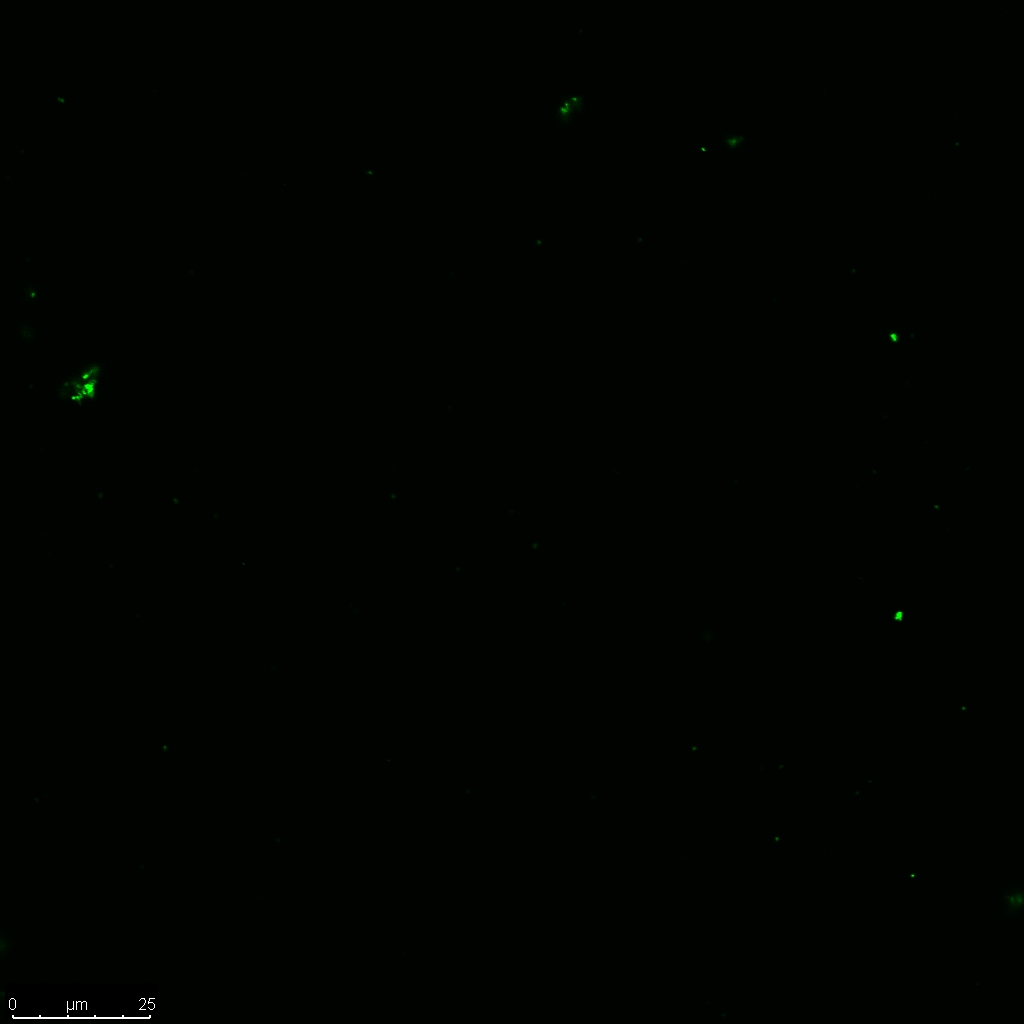

Supplement: Figure 2—source data 3. [file elife-94795-fig2-data3.zip › Figure 2B/replicate III/Figure 2B panel 6 Luc agg replicate 3 photo 3.jpg]

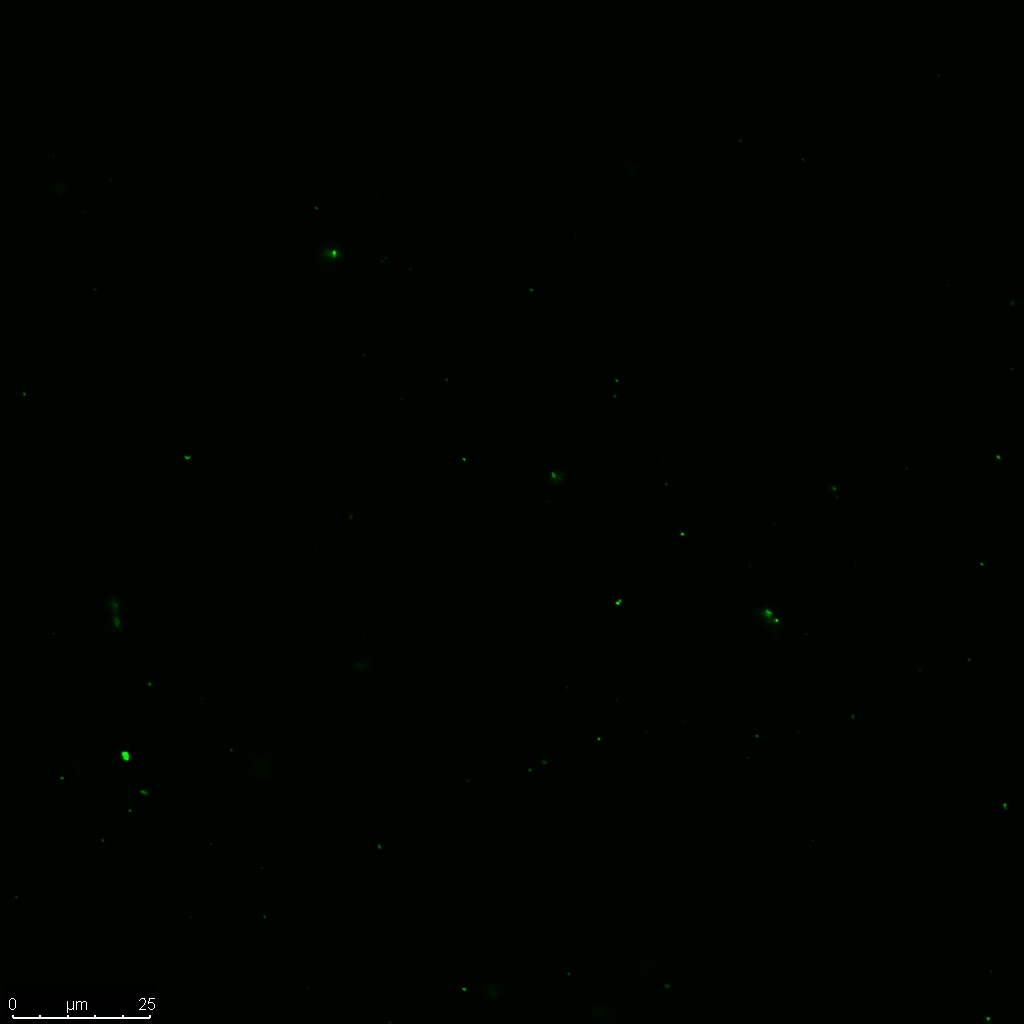

Supplement: Figure 2—source data 3. [file elife-94795-fig2-data3.zip › Figure 2B/replicate III/Figure 2B panel 6 Luc agg replicate 3 photo 4.jpg]

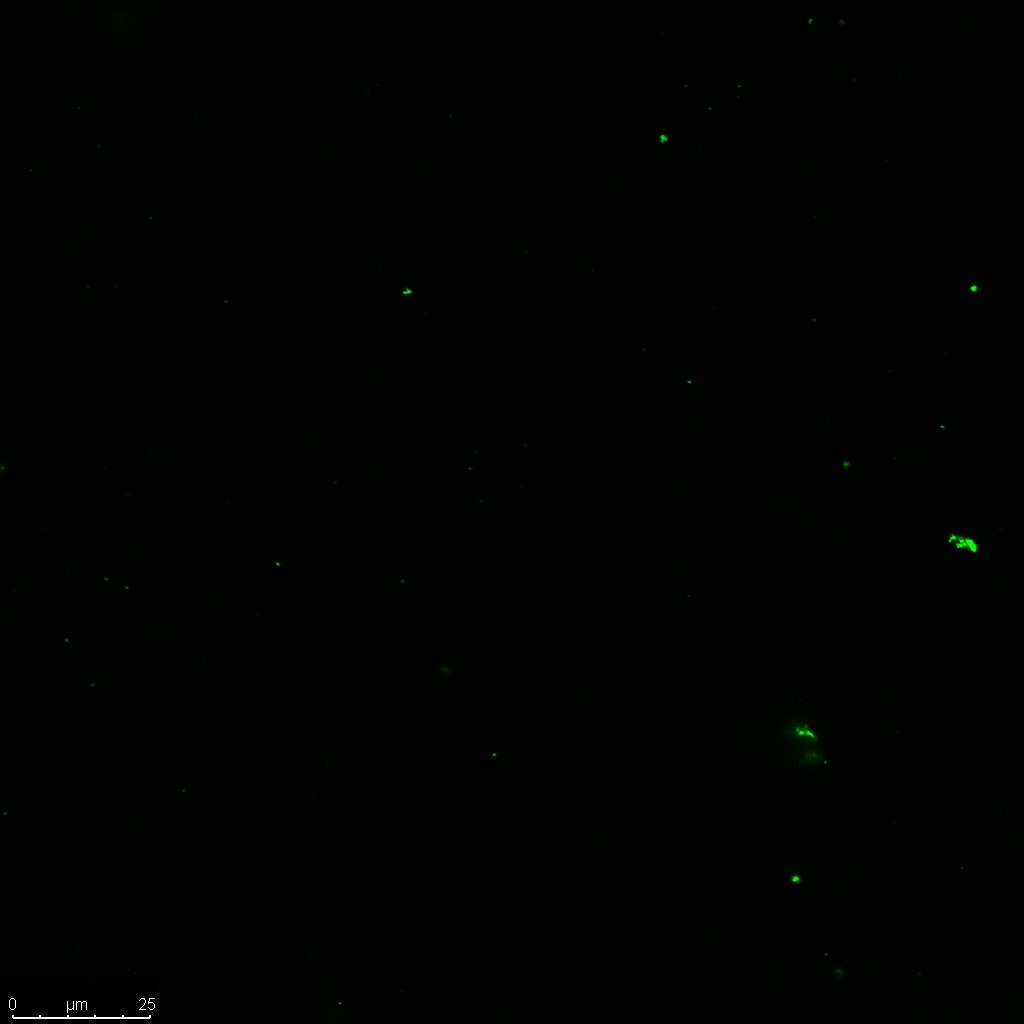

Supplement: Figure 2—source data 3. [file elife-94795-fig2-data3.zip › Figure 2B/replicate III/Figure 2B panel 6 Luc agg replicate 3 photo 5.jpg]

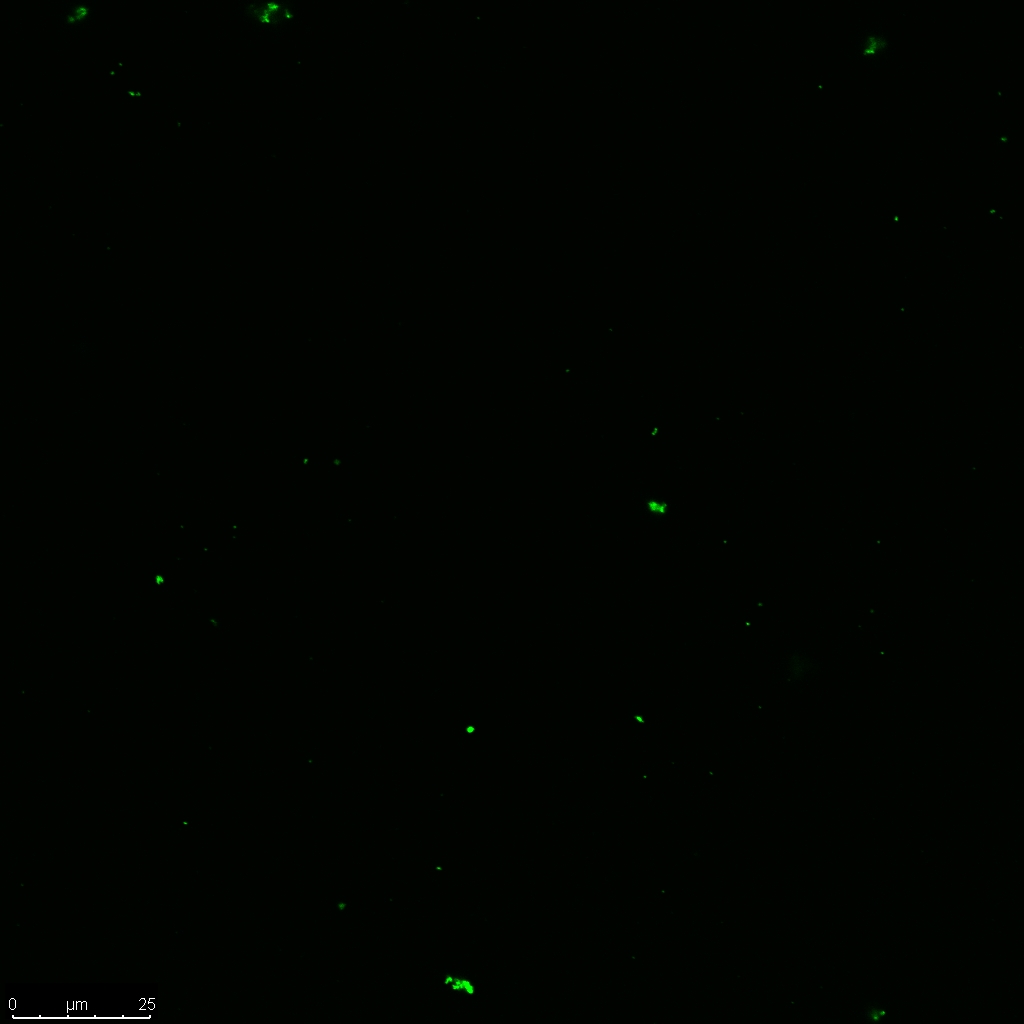

Supplement: Figure 2—source data 3. [file elife-94795-fig2-data3.zip › Figure 2B/replicate III/Figure 2B panel 6 Luc agg replicate 3 photo 6.jpg]

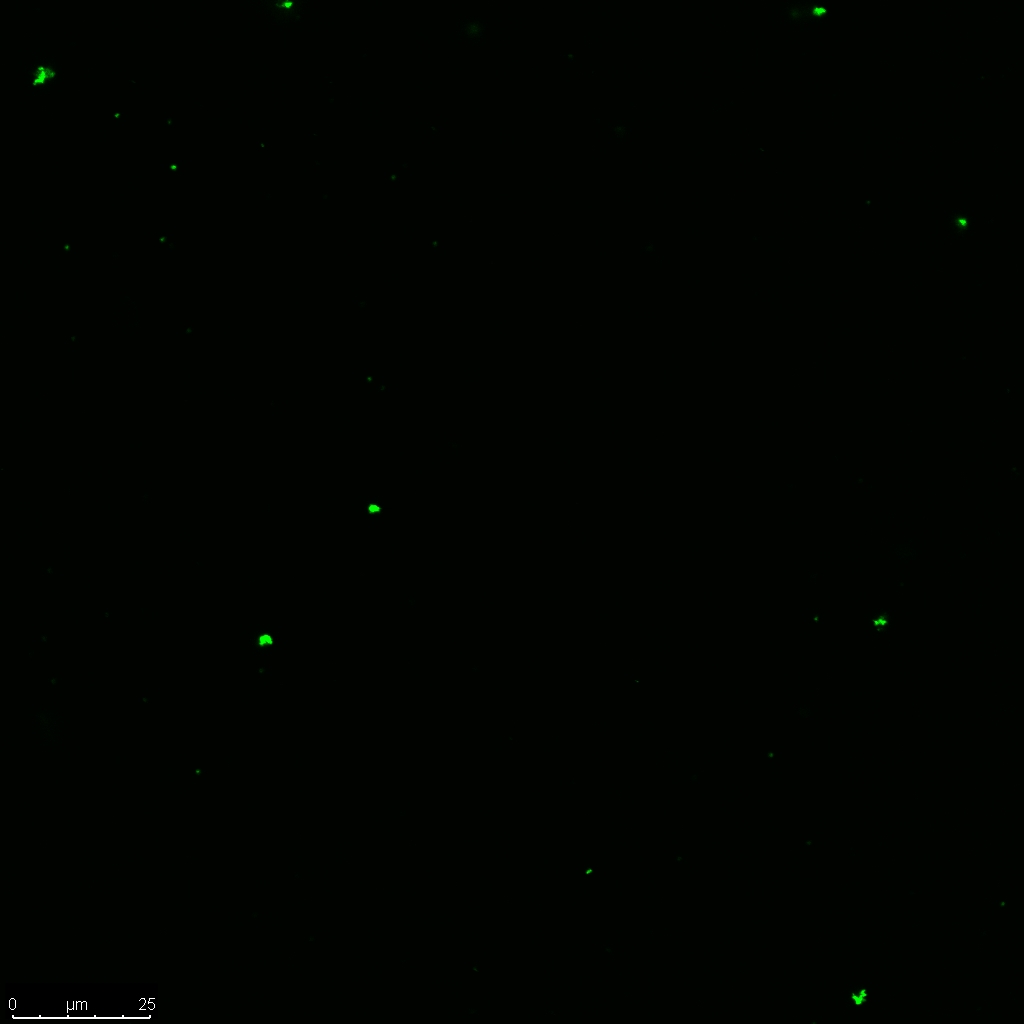

Supplement: Figure 2—source data 3. [file elife-94795-fig2-data3.zip › Figure 2B/replicate III/Figure 2B panel 6 Luc agg replicate 3 photo 7.jpg]

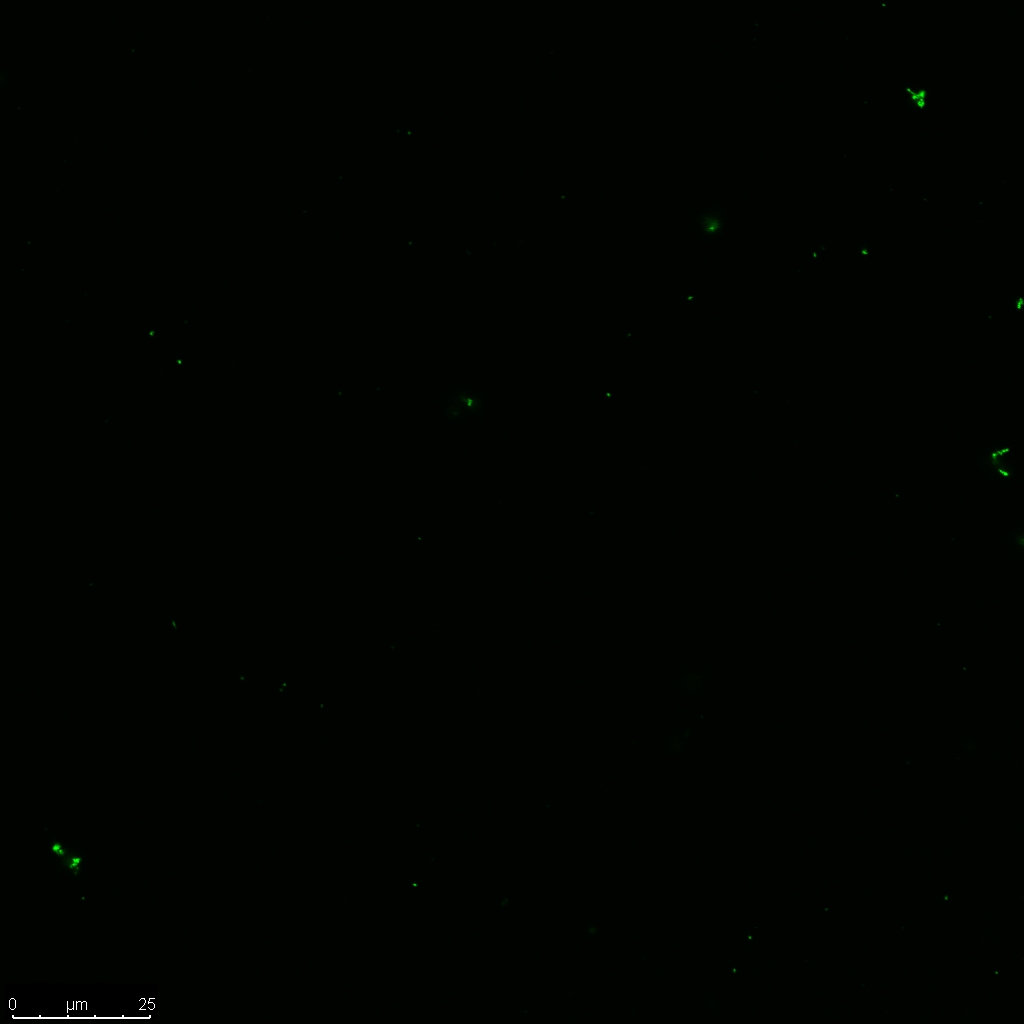

Supplement: Figure 2—source data 3. [file elife-94795-fig2-data3.zip › Figure 2B/replicate III/Figure 2B panel 6 Luc agg replicate 3 photo 8.jpg]

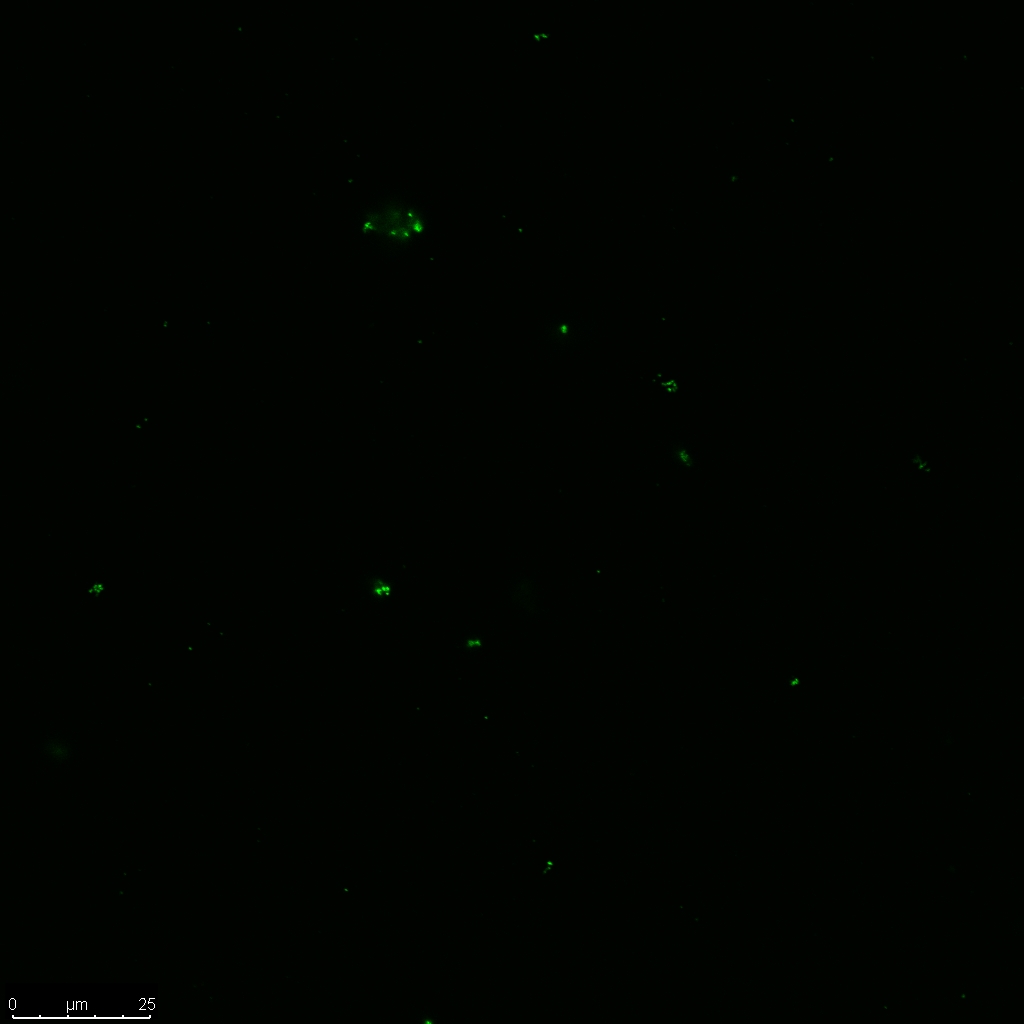

Supplement: Figure 2—source data 3. [file elife-94795-fig2-data3.zip › Figure 2B/replicate III/Figure 2B panel 6 Luc agg replicate 3 photo 9.jpg]

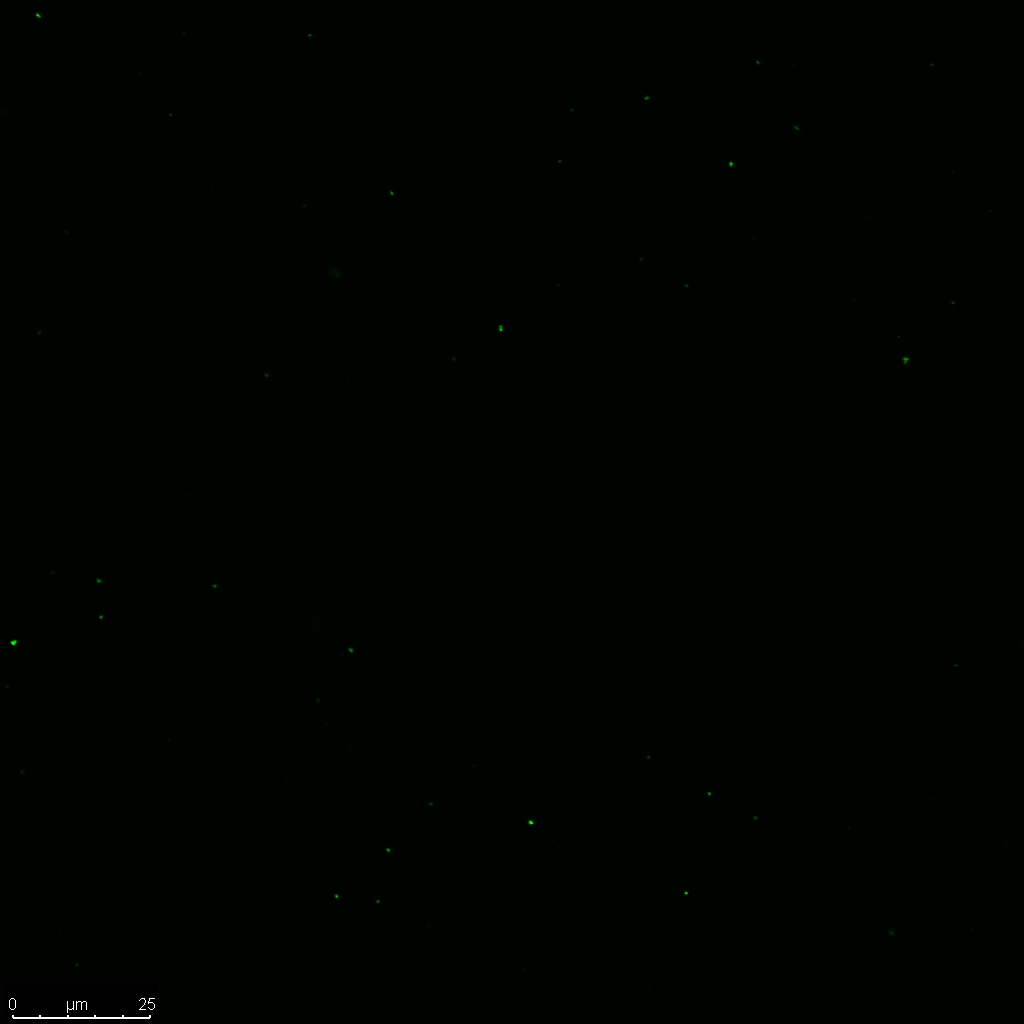

Supplement: Figure 2—source data 3. [file elife-94795-fig2-data3.zip › Figure 2B/replicate III/Figure 2B panel 6 Luc agg replicate 3 photo 10.jpg]

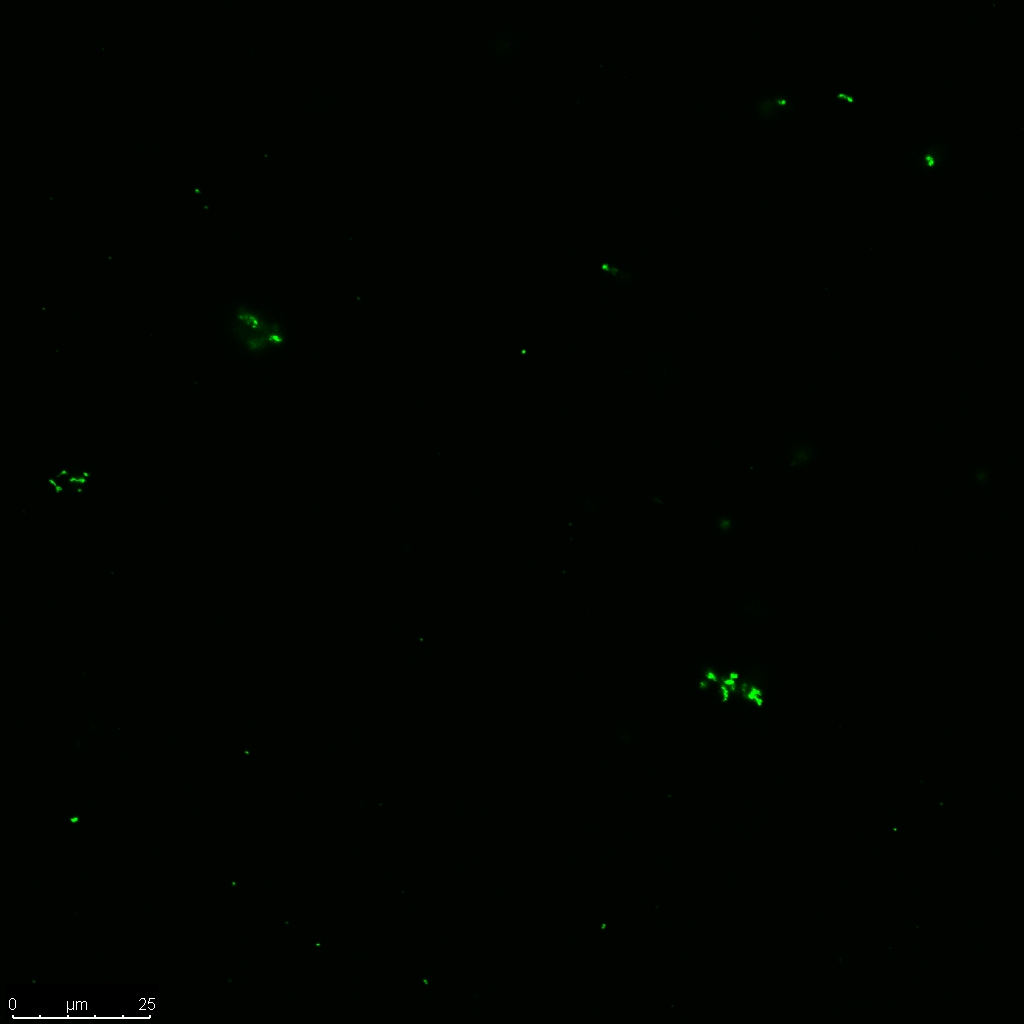

Supplement: Figure 2—source data 3. [file elife-94795-fig2-data3.zip › Figure 2B/replicate III/Figure 2B panel 5 Luc agg replicate 3 photo 1.jpg]

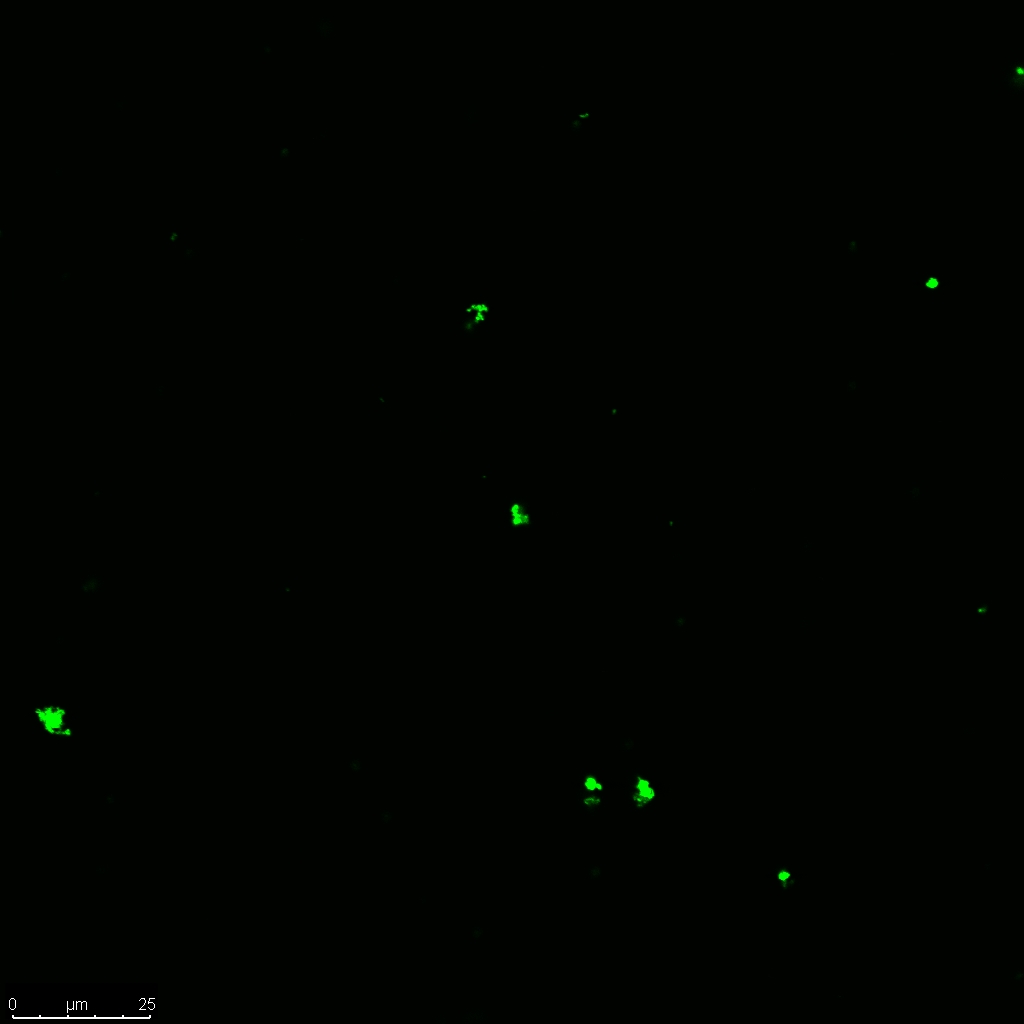

Supplement: Figure 2—source data 3. [file elife-94795-fig2-data3.zip › Figure 2B/replicate III/Figure 2B panel 5 Luc agg replicate 3 photo 2.jpg]

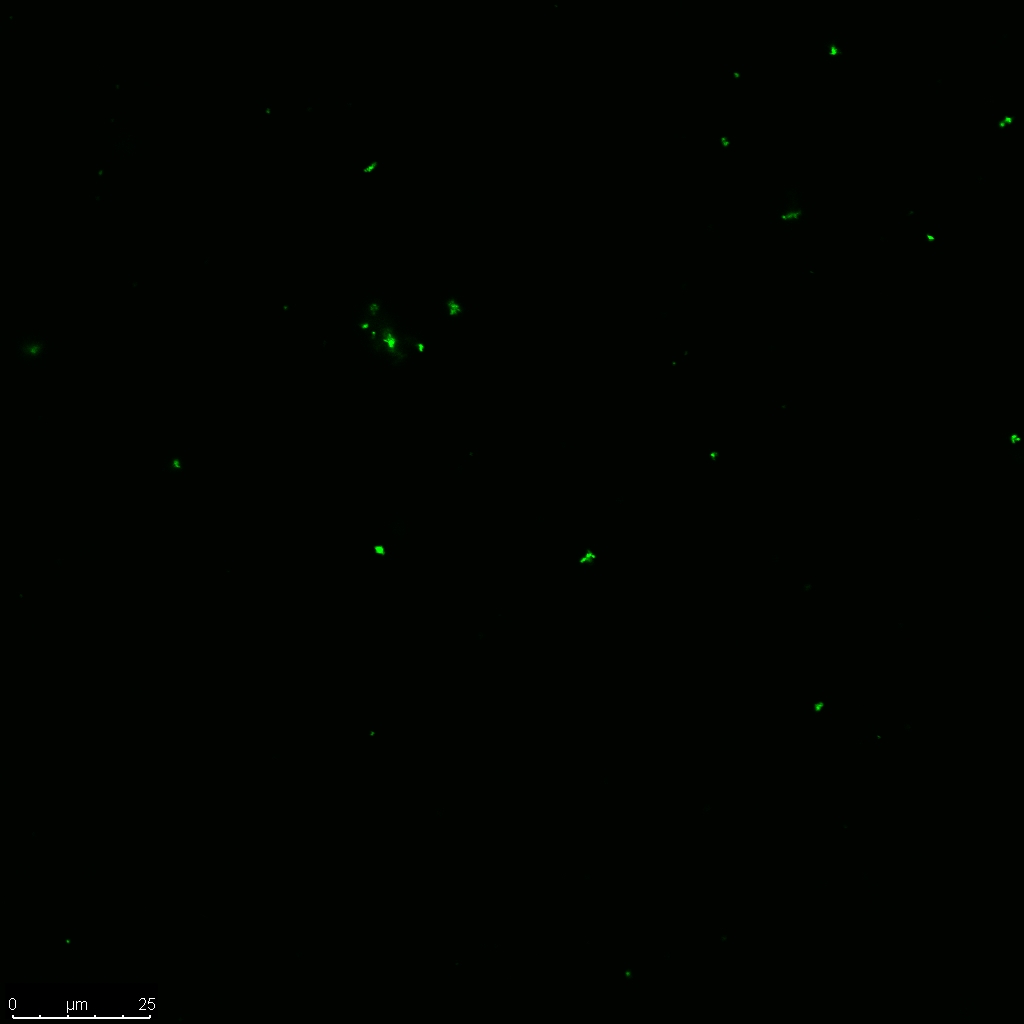

Supplement: Figure 2—source data 3. [file elife-94795-fig2-data3.zip › Figure 2B/replicate III/Figure 2B panel 5 Luc agg replicate 3 photo 3.jpg]

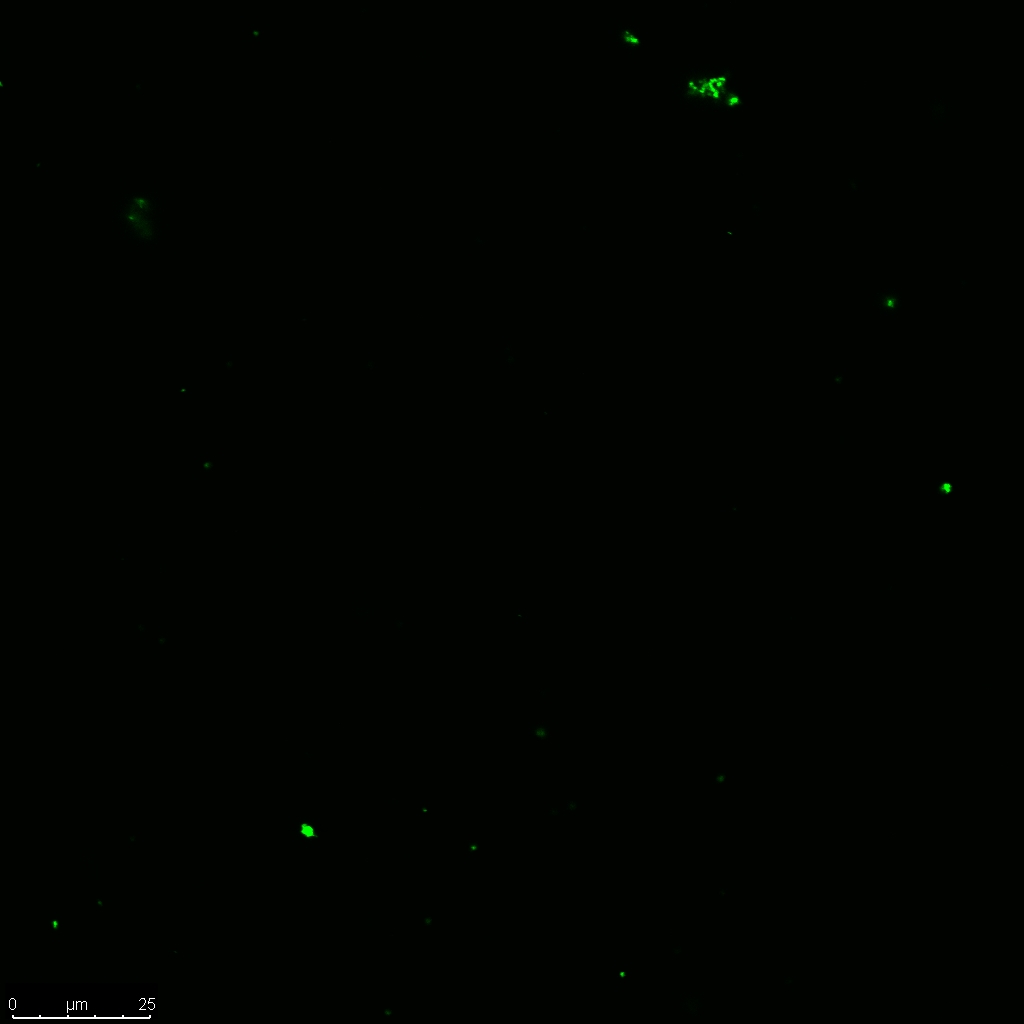

Supplement: Figure 2—source data 3. [file elife-94795-fig2-data3.zip › Figure 2B/replicate III/Figure 2B panel 5 Luc agg replicate 3 photo 4.jpg]

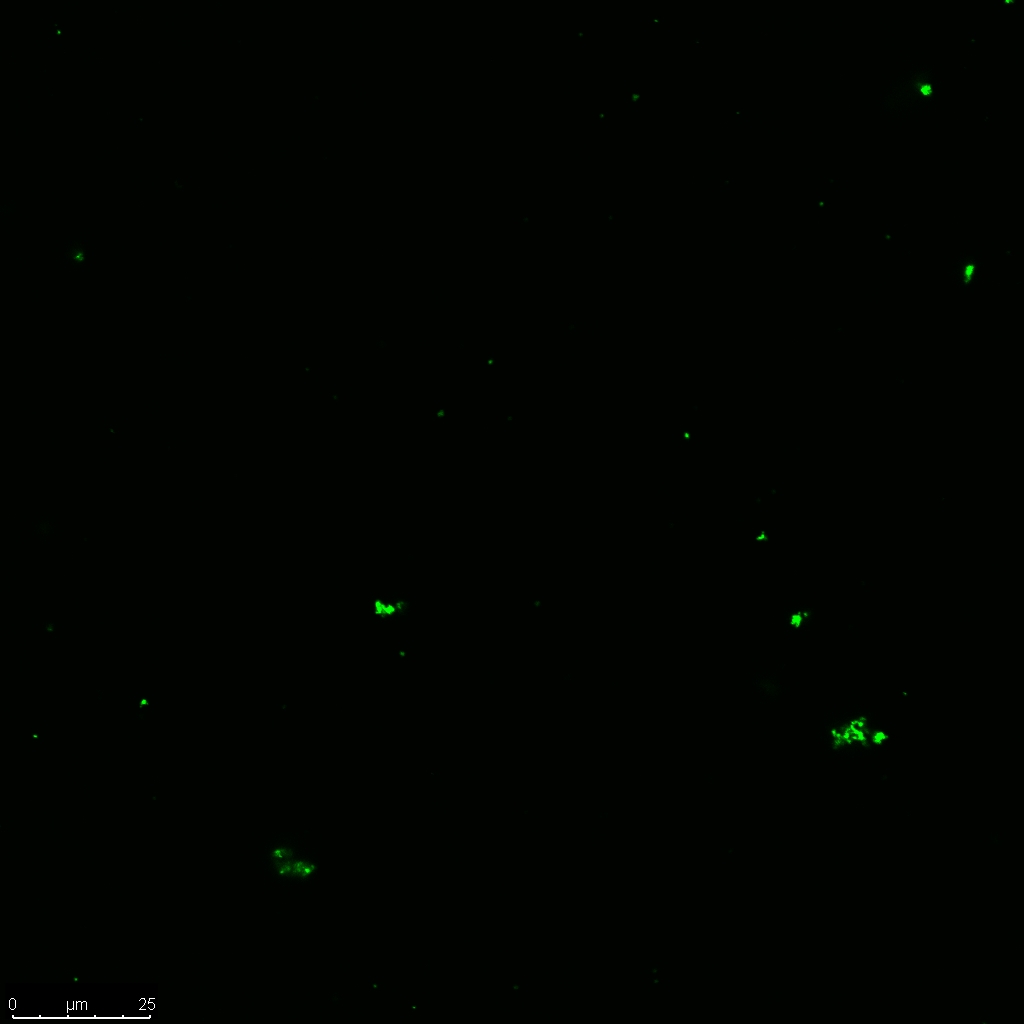

Supplement: Figure 2—source data 3. [file elife-94795-fig2-data3.zip › Figure 2B/replicate III/Figure 2B panel 5 Luc agg replicate 3 photo 5.jpg]

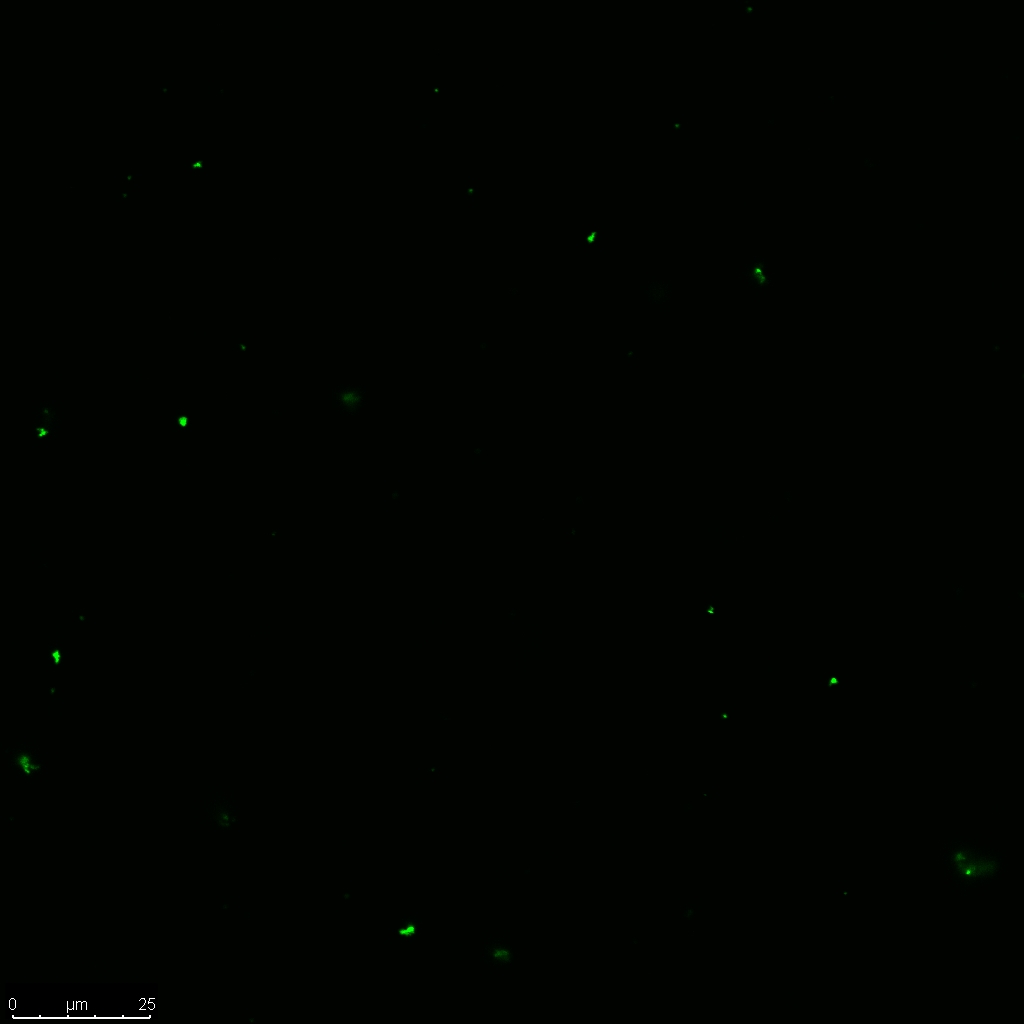

Supplement: Figure 2—source data 3. [file elife-94795-fig2-data3.zip › Figure 2B/replicate III/Figure 2B panel 5 Luc agg replicate 3 photo 6.jpg]

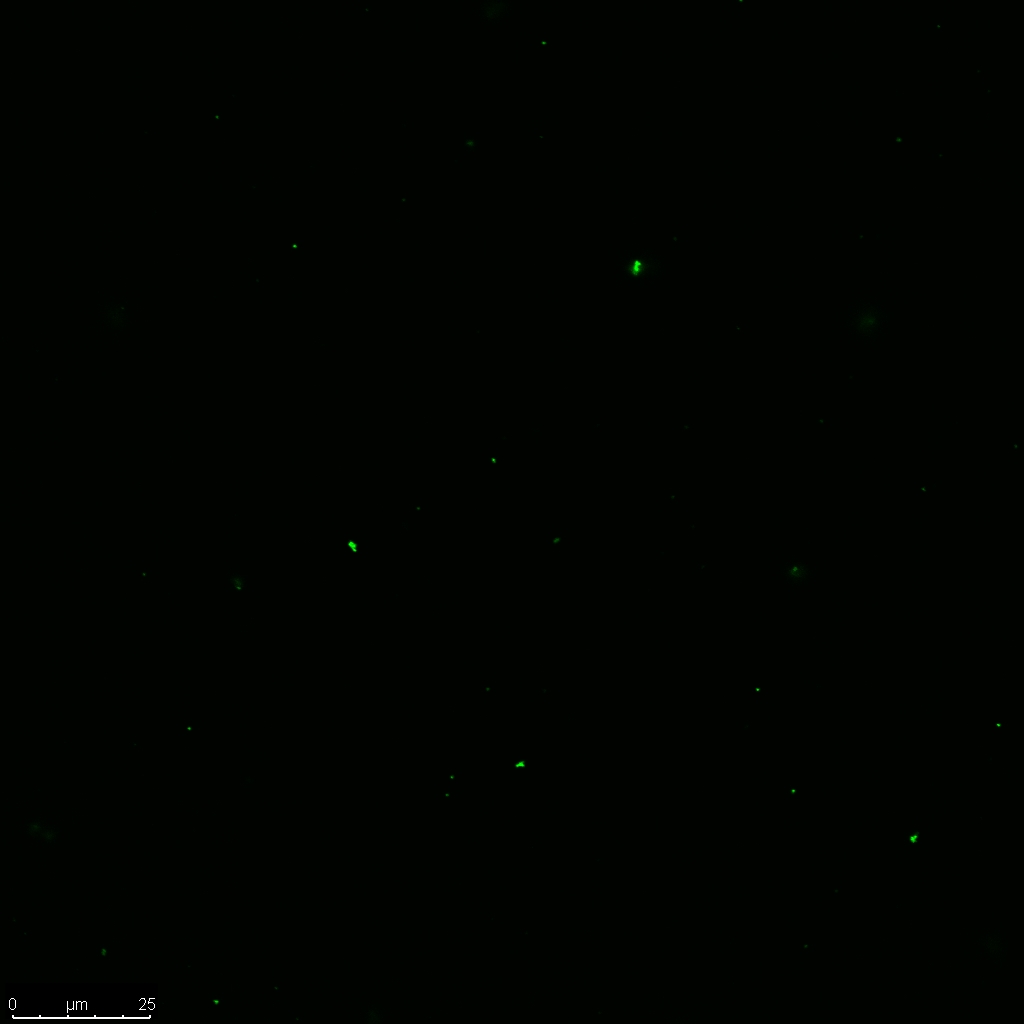

Supplement: Figure 2—source data 3. [file elife-94795-fig2-data3.zip › Figure 2B/replicate III/Figure 2B panel 5 Luc agg replicate 3 photo 7.jpg]

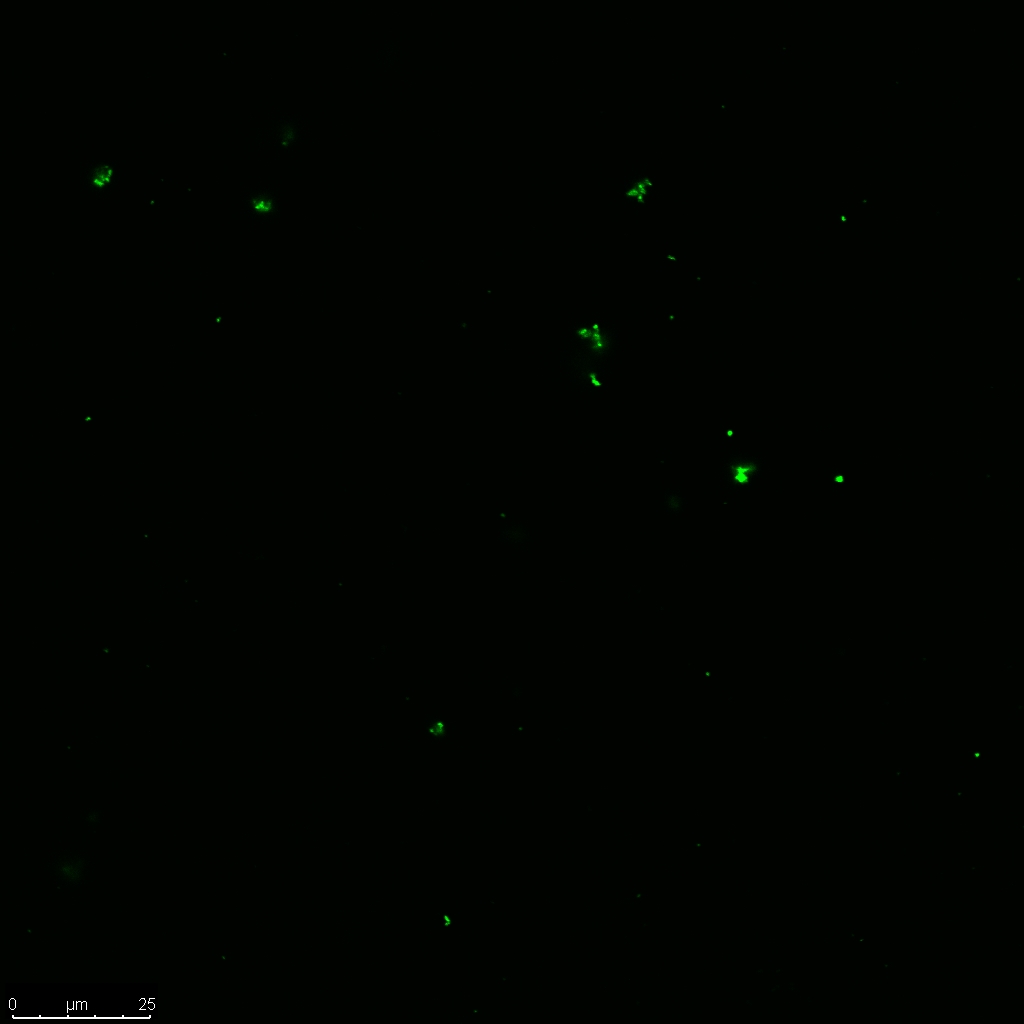

Supplement: Figure 2—source data 3. [file elife-94795-fig2-data3.zip › Figure 2B/replicate III/Figure 2B panel 5 Luc agg replicate 3 photo 8.jpg]

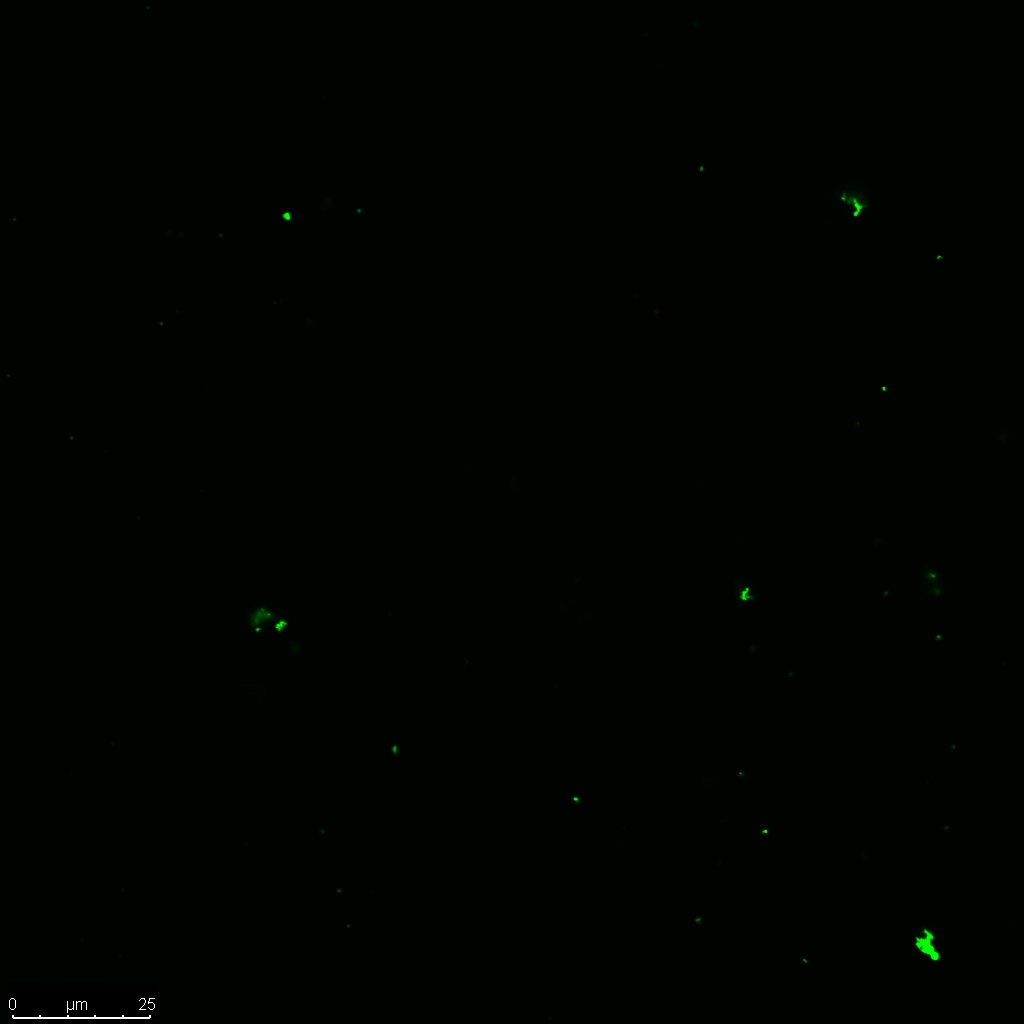

Supplement: Figure 2—source data 3. [file elife-94795-fig2-data3.zip › Figure 2B/replicate III/Figure 2B panel 5 Luc agg replicate 3 photo 9.jpg]

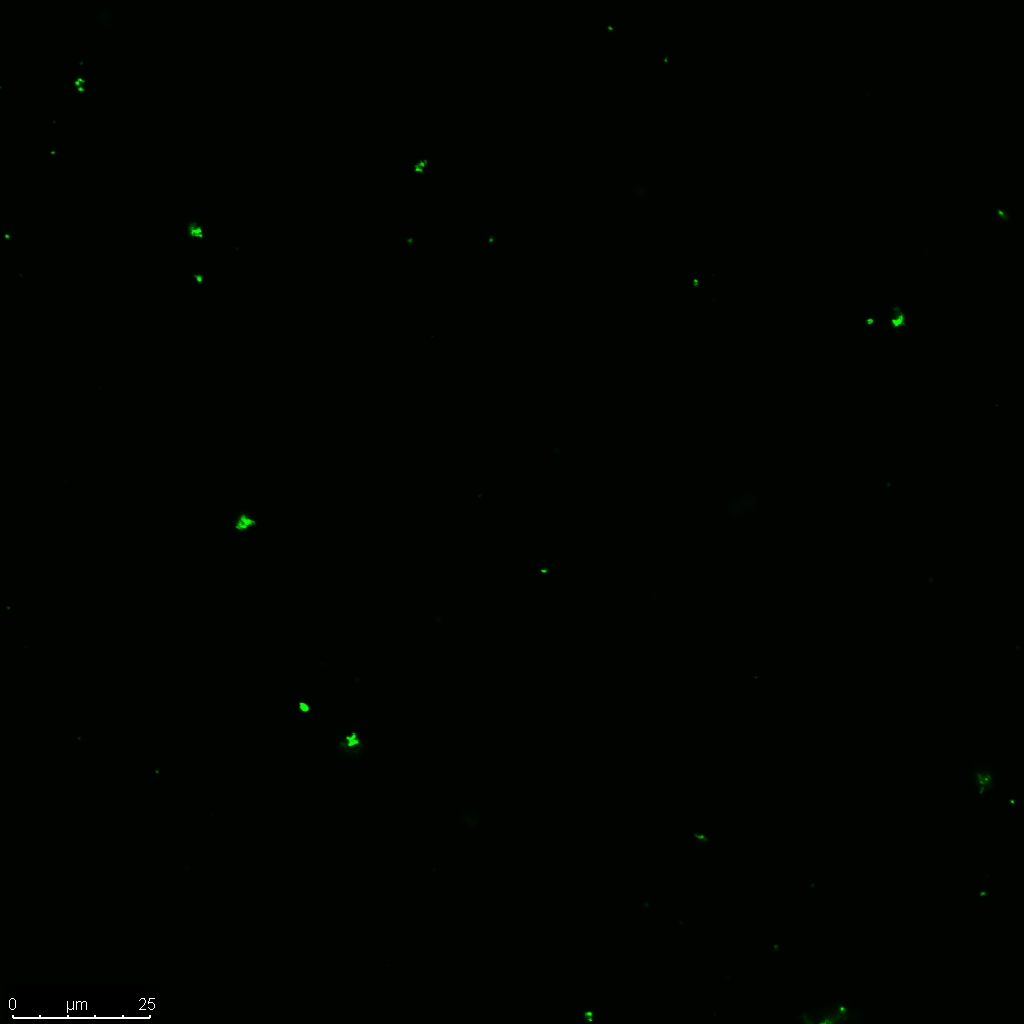

Supplement: Figure 2—source data 3. [file elife-94795-fig2-data3.zip › Figure 2B/replicate III/Figure 2B panel 5 Luc agg replicate 3 photo 10.jpg]

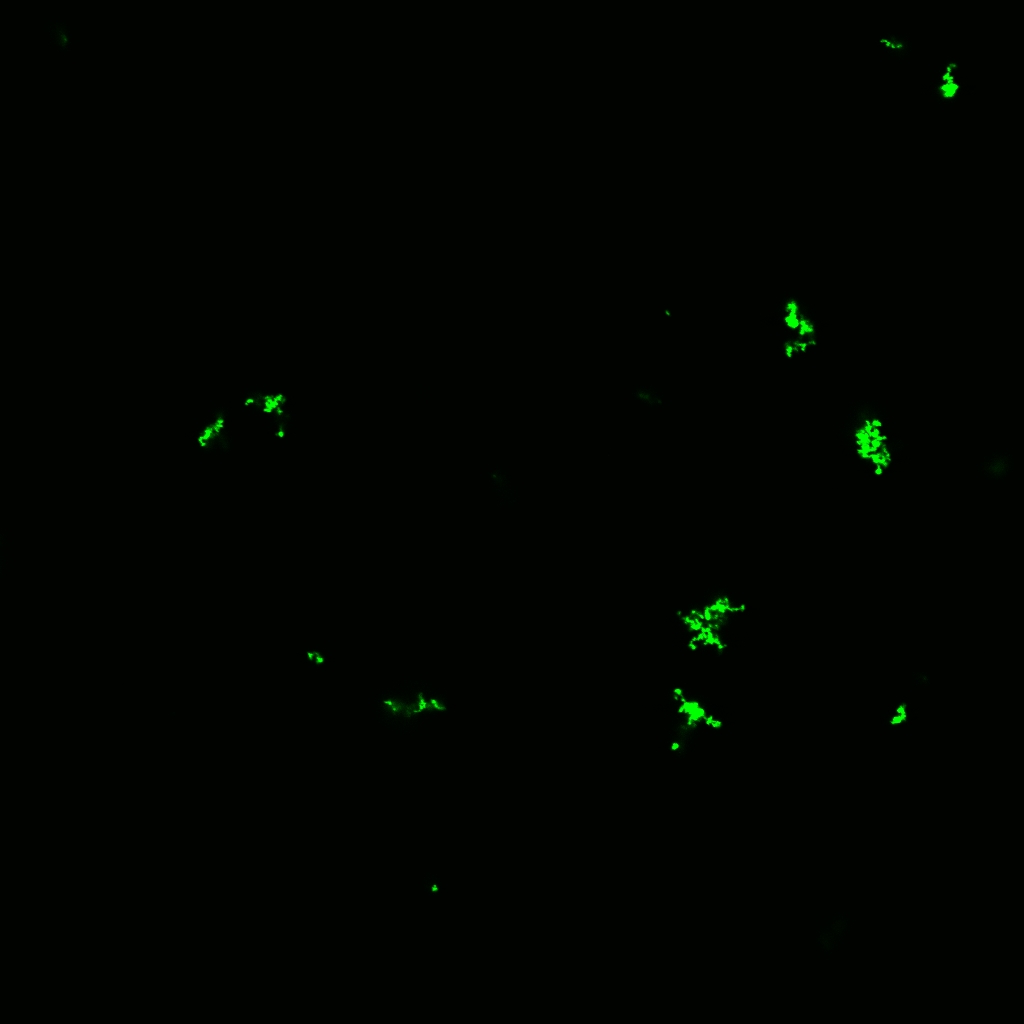

Supplement: Figure 2—source data 3. [file elife-94795-fig2-data3.zip › Figure 2B/Figure 2B panel 4 Luc agg SSS no ATP.jpg]

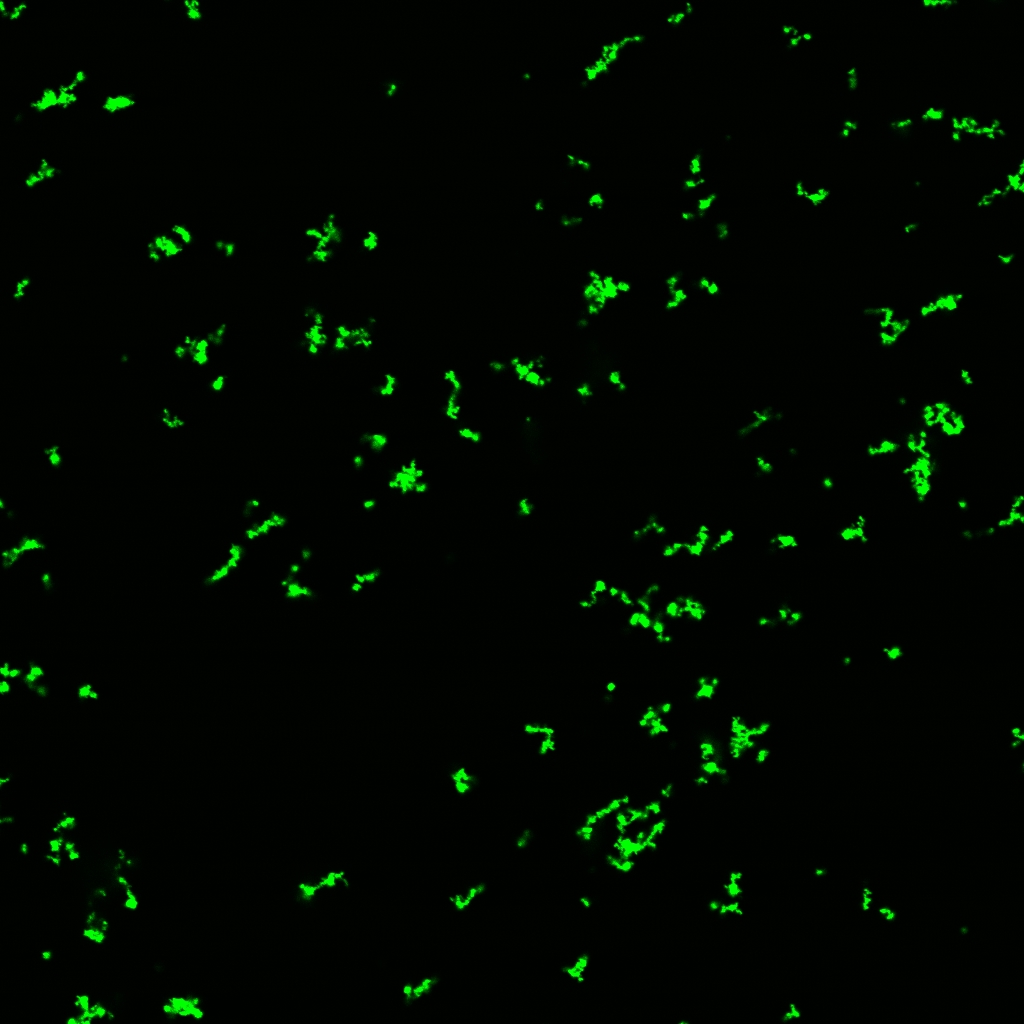

Supplement: Figure 2—source data 3. [file elife-94795-fig2-data3.zip › Figure 2B/Figure 2B panel 1 Luc agg.jpg]

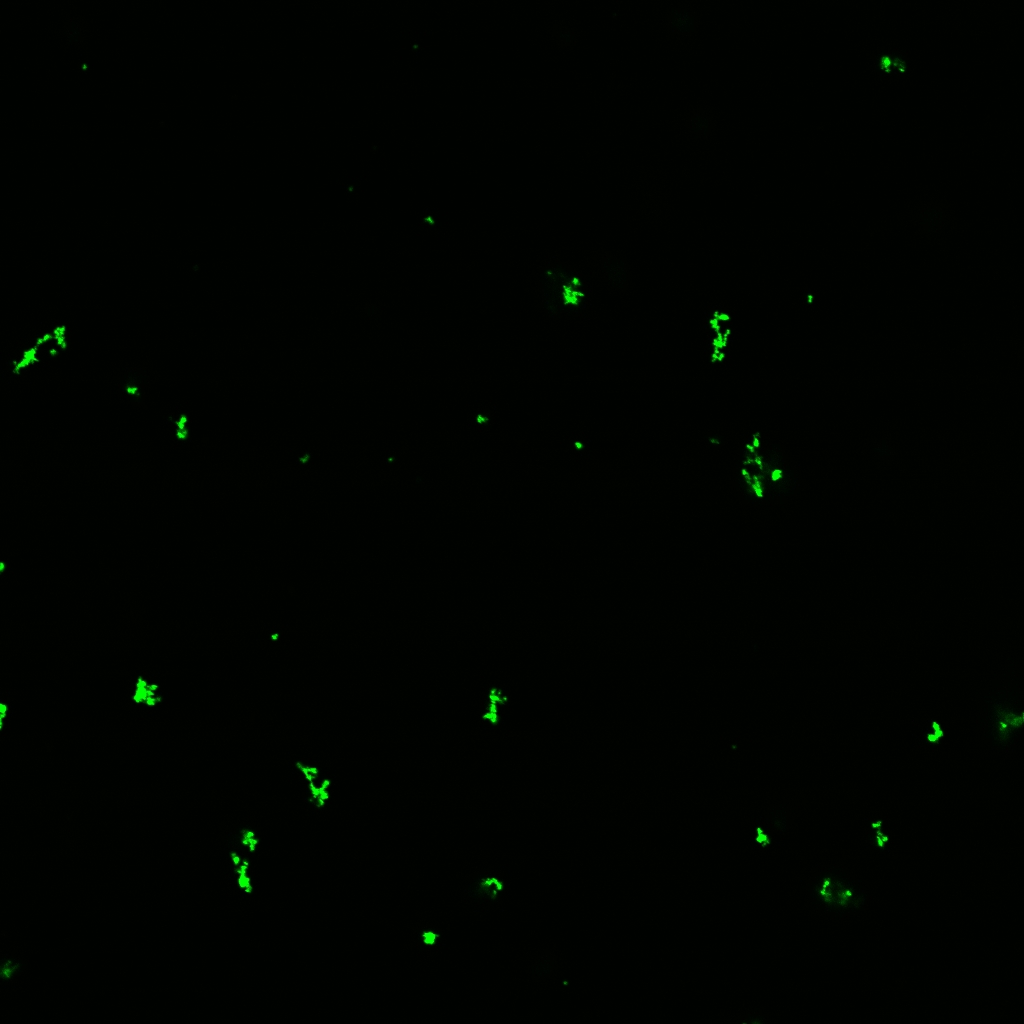

Supplement: Figure 2—source data 3. [file elife-94795-fig2-data3.zip › Figure 2B/Figure 2B panel 2 Luc agg YS.jpg]

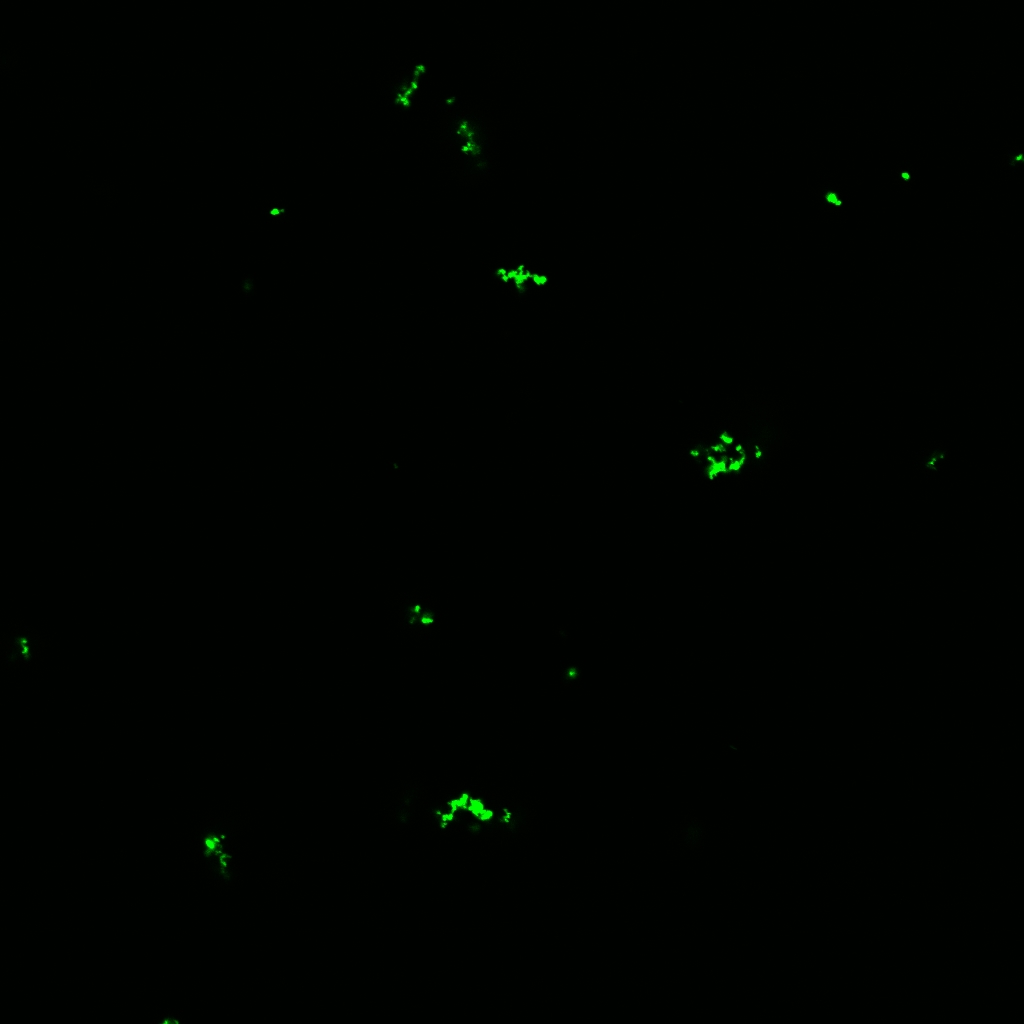

Supplement: Figure 2—source data 3. [file elife-94795-fig2-data3.zip › Figure 2B/Figure 2B panel 3 Luc agg YSS.jpg]

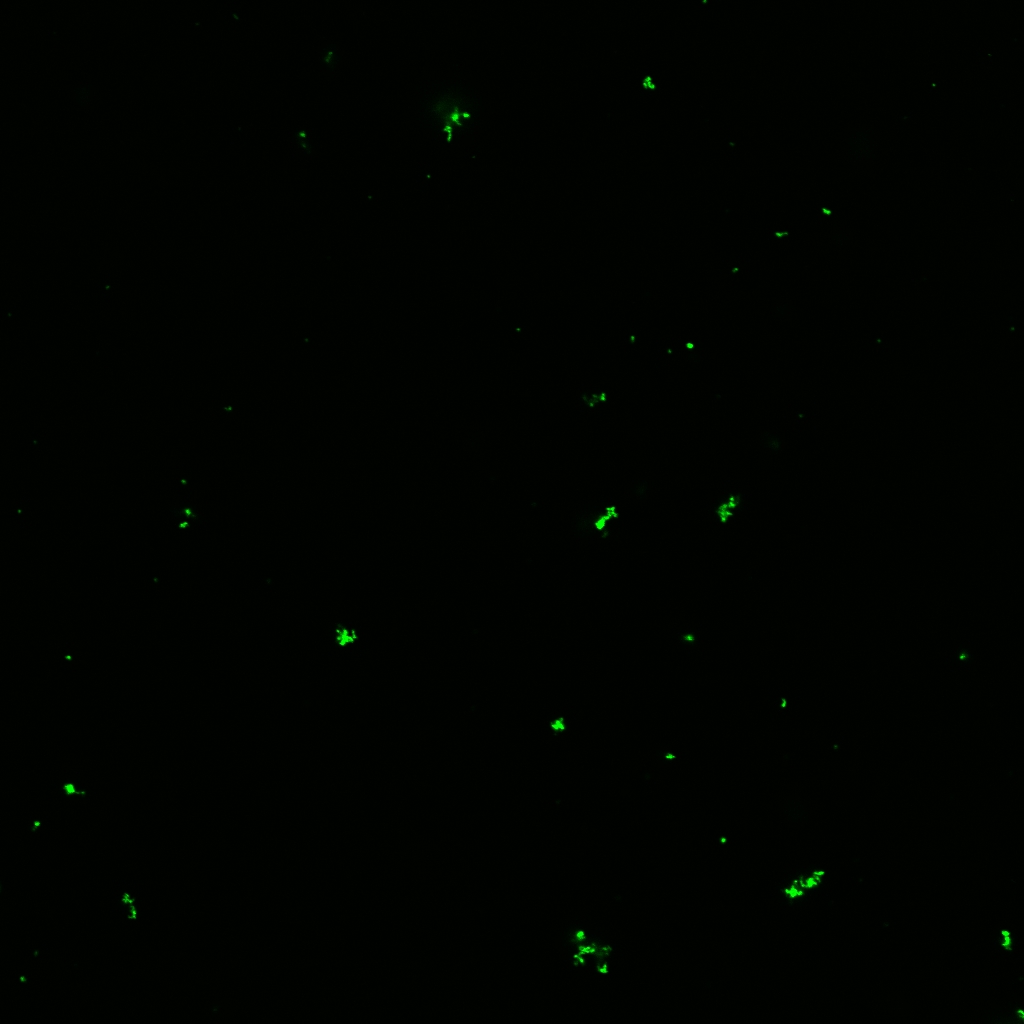

Supplement: Figure 2—source data 3. [file elife-94795-fig2-data3.zip › Figure 2B/Figure 2B panel 5 Luc agg SS.jpg]

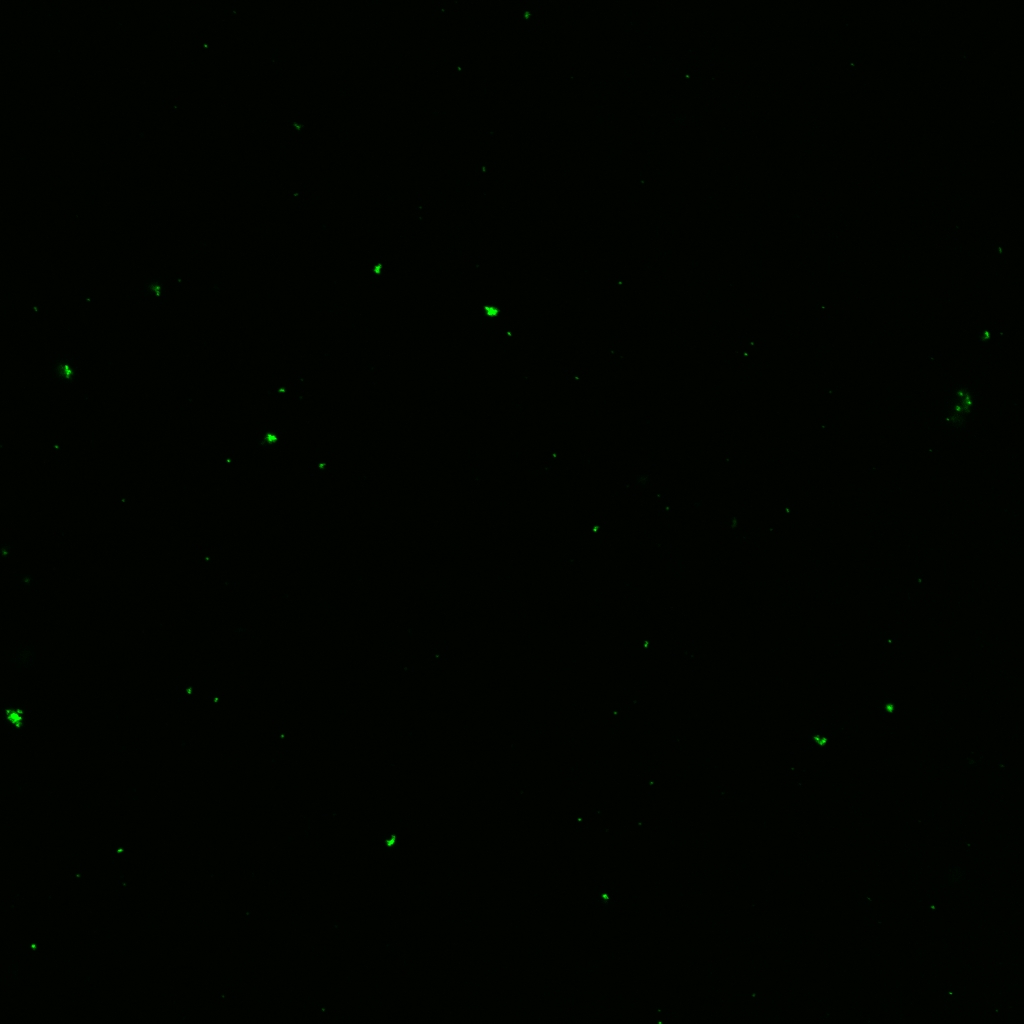

Supplement: Figure 2—source data 3. [file elife-94795-fig2-data3.zip › Figure 2B/Figure 2B panel 6 Luc agg SSS.jpg]

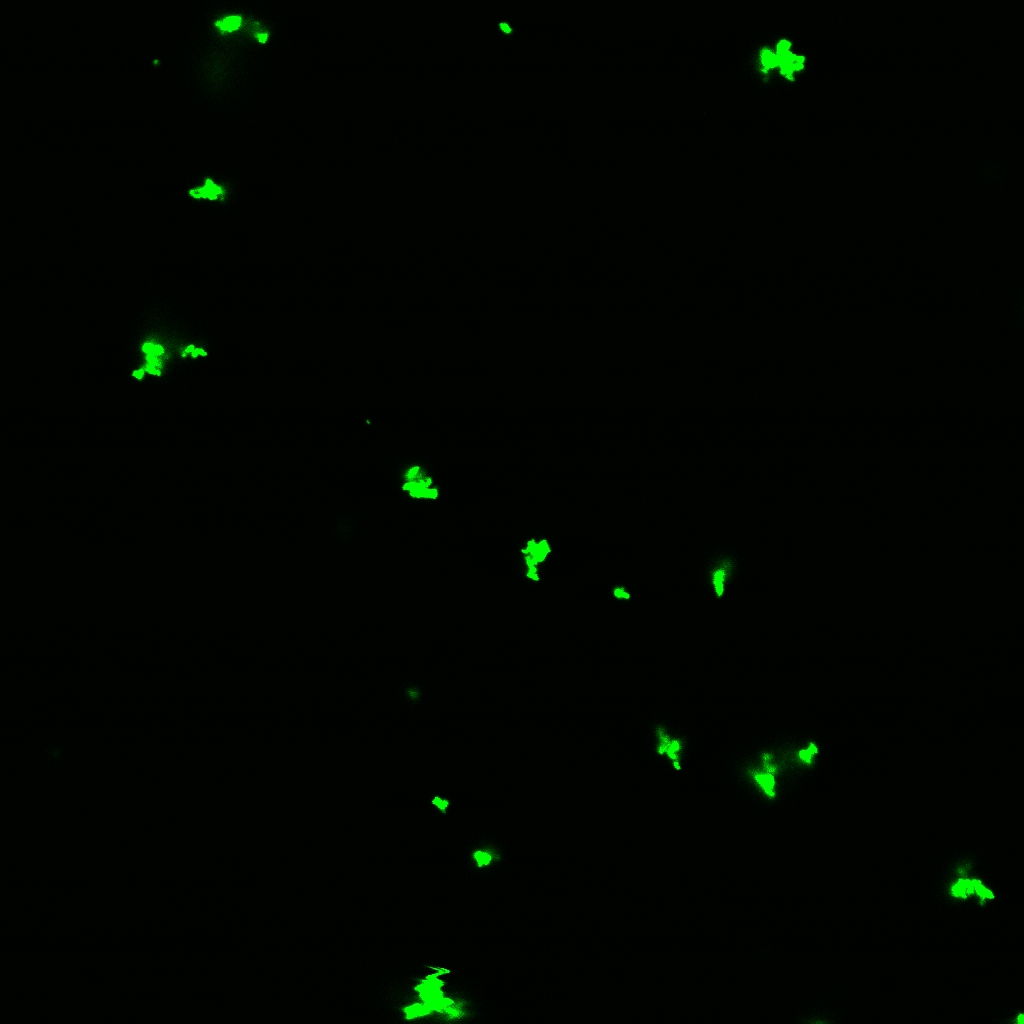

Supplement: Figure 3—figure supplement 1—source data 3. [file elife-94795-fig3-figsupp1-data3.zip › Figure 3—figure supplement 1B/Figure 3—figure supplement 1B.jpg]

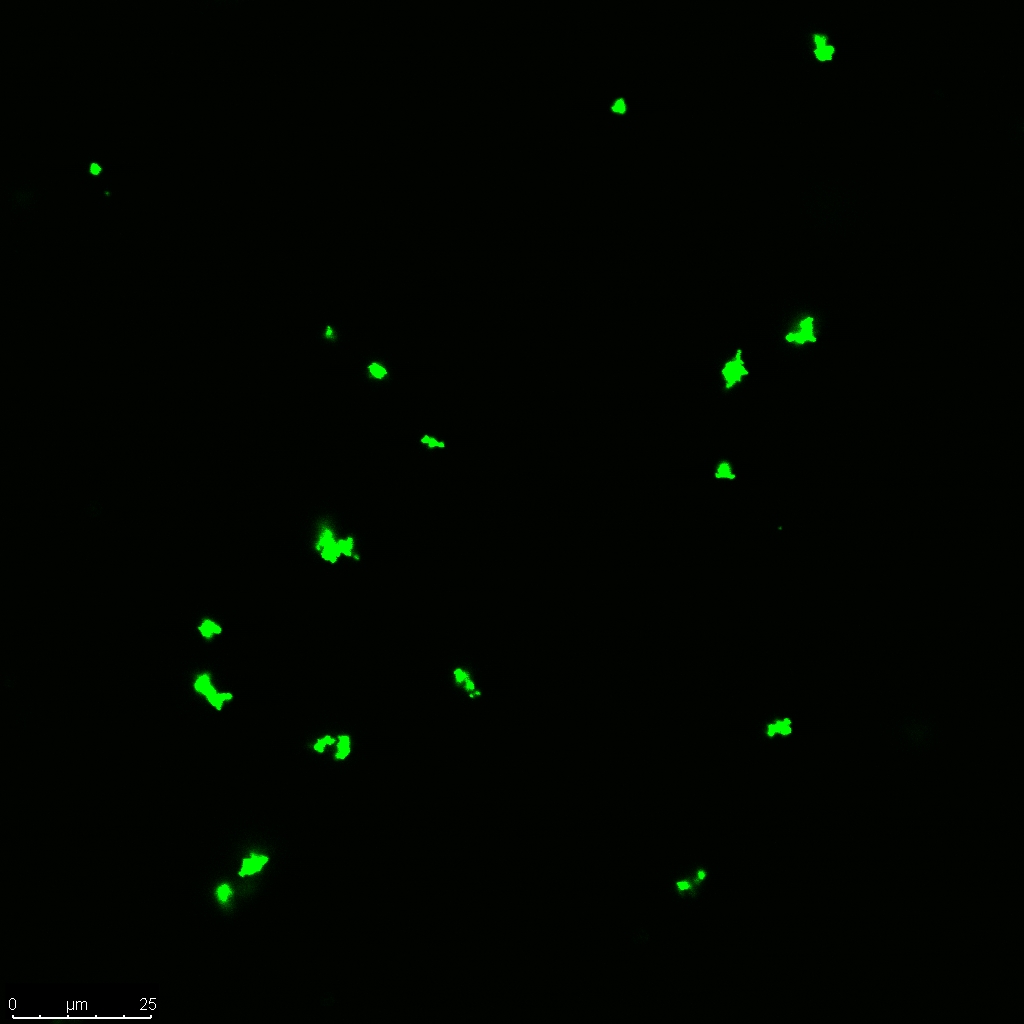

Supplement: Figure 3—figure supplement 1—source data 3. [file elife-94795-fig3-figsupp1-data3.zip › Figure 3—figure supplement 1B/replicate I/Figure 3—figure supplement 1B replicate 1 photo 1.jpg]

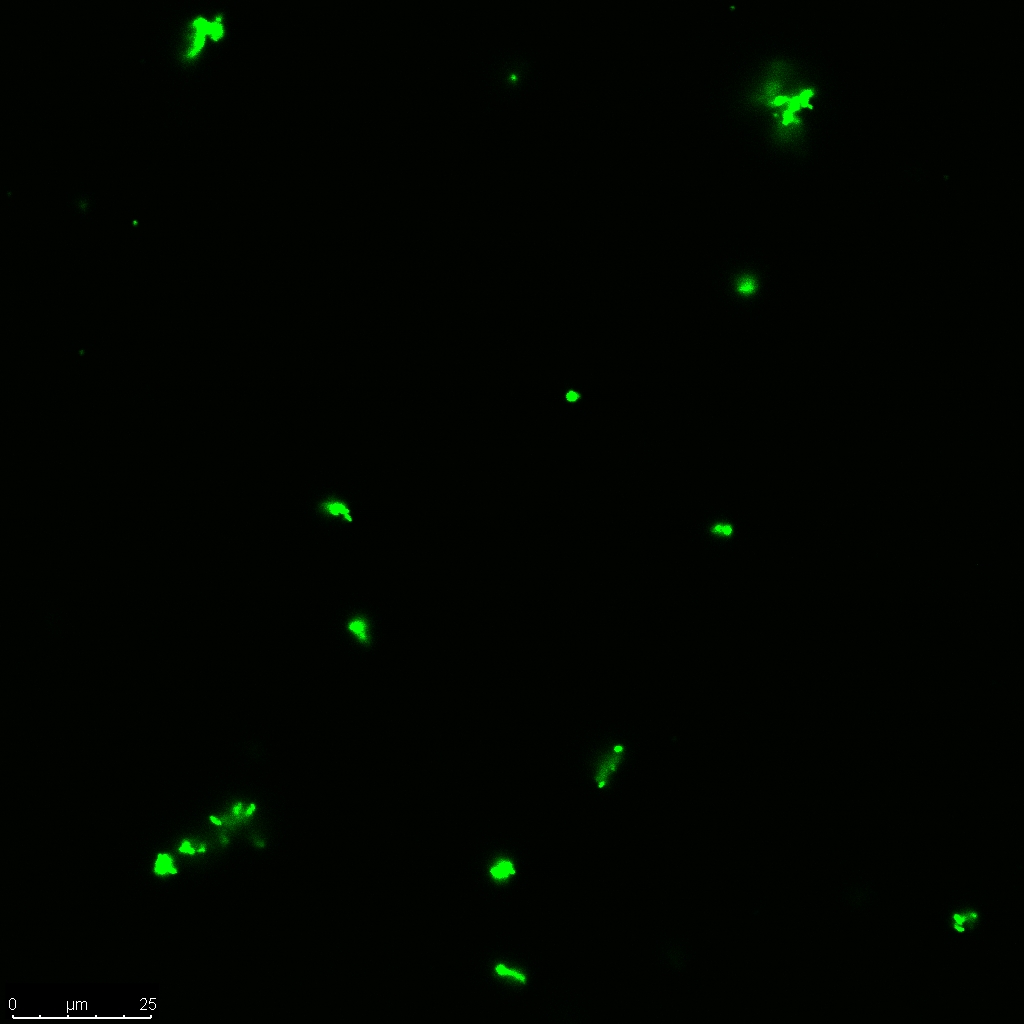

Supplement: Figure 3—figure supplement 1—source data 3. [file elife-94795-fig3-figsupp1-data3.zip › Figure 3—figure supplement 1B/replicate I/Figure 3—figure supplement 1B replicate 1 photo 2.jpg]

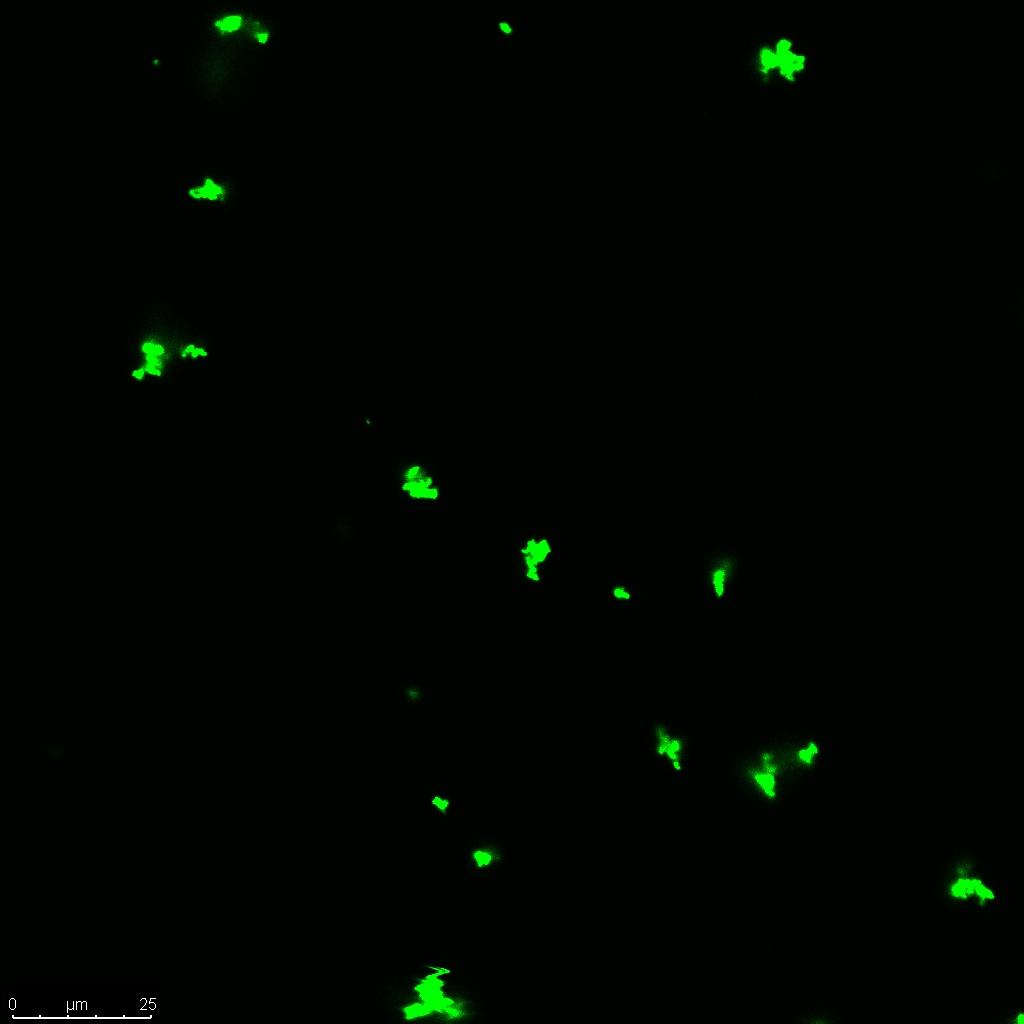

Supplement: Figure 3—figure supplement 1—source data 3. [file elife-94795-fig3-figsupp1-data3.zip › Figure 3—figure supplement 1B/replicate I/Figure 3—figure supplement 1B replicate 1 photo 3.jpg]

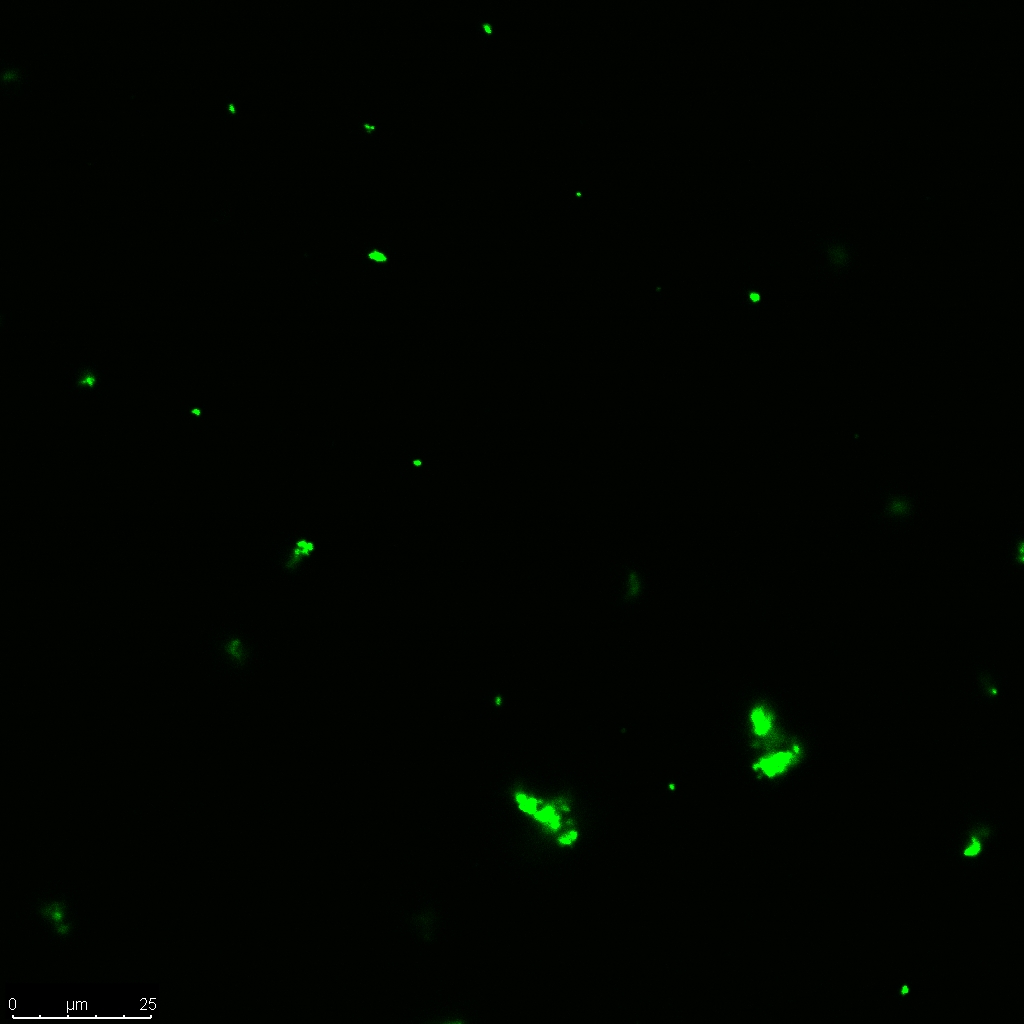

Supplement: Figure 3—figure supplement 1—source data 3. [file elife-94795-fig3-figsupp1-data3.zip › Figure 3—figure supplement 1B/replicate I/Figure 3—figure supplement 1B replicate 1 photo 4.jpg]

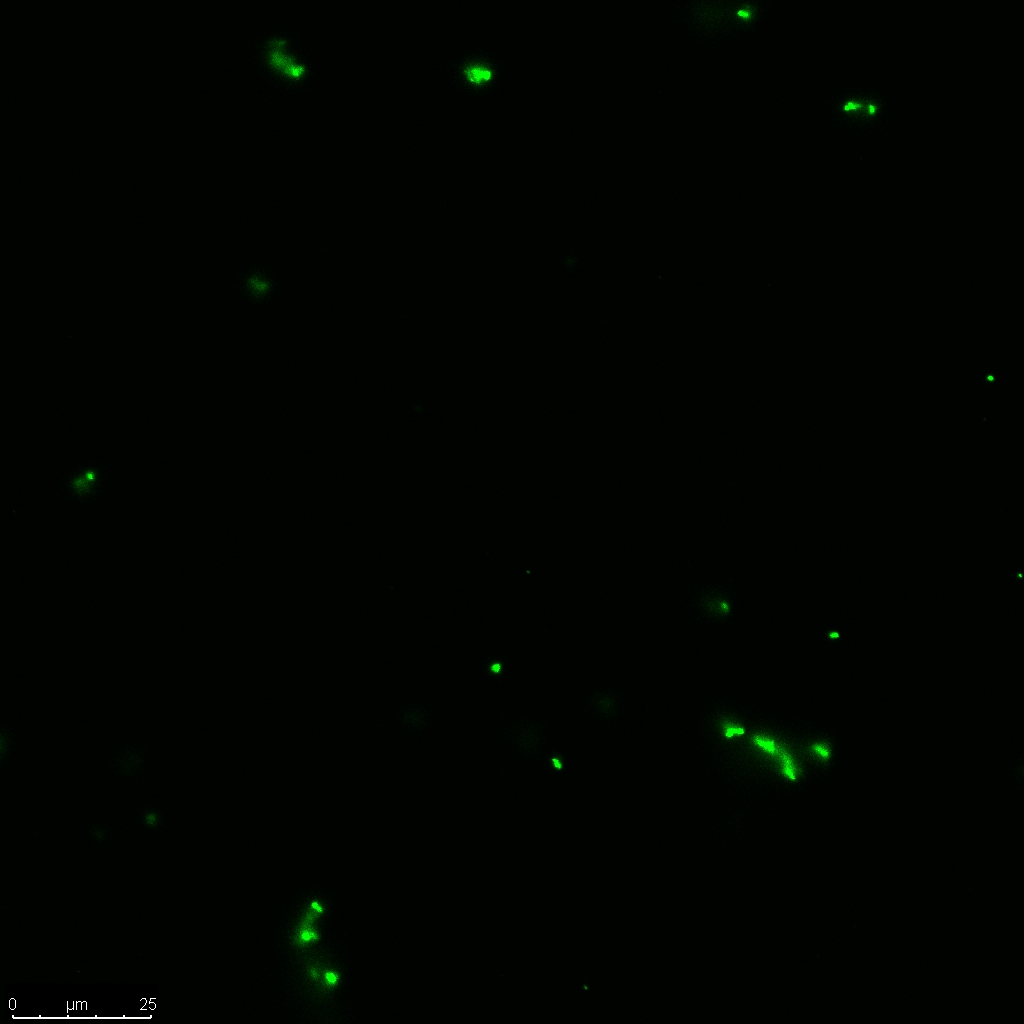

Supplement: Figure 3—figure supplement 1—source data 3. [file elife-94795-fig3-figsupp1-data3.zip › Figure 3—figure supplement 1B/replicate I/Figure 3—figure supplement 1B replicate 1 photo 5.jpg]

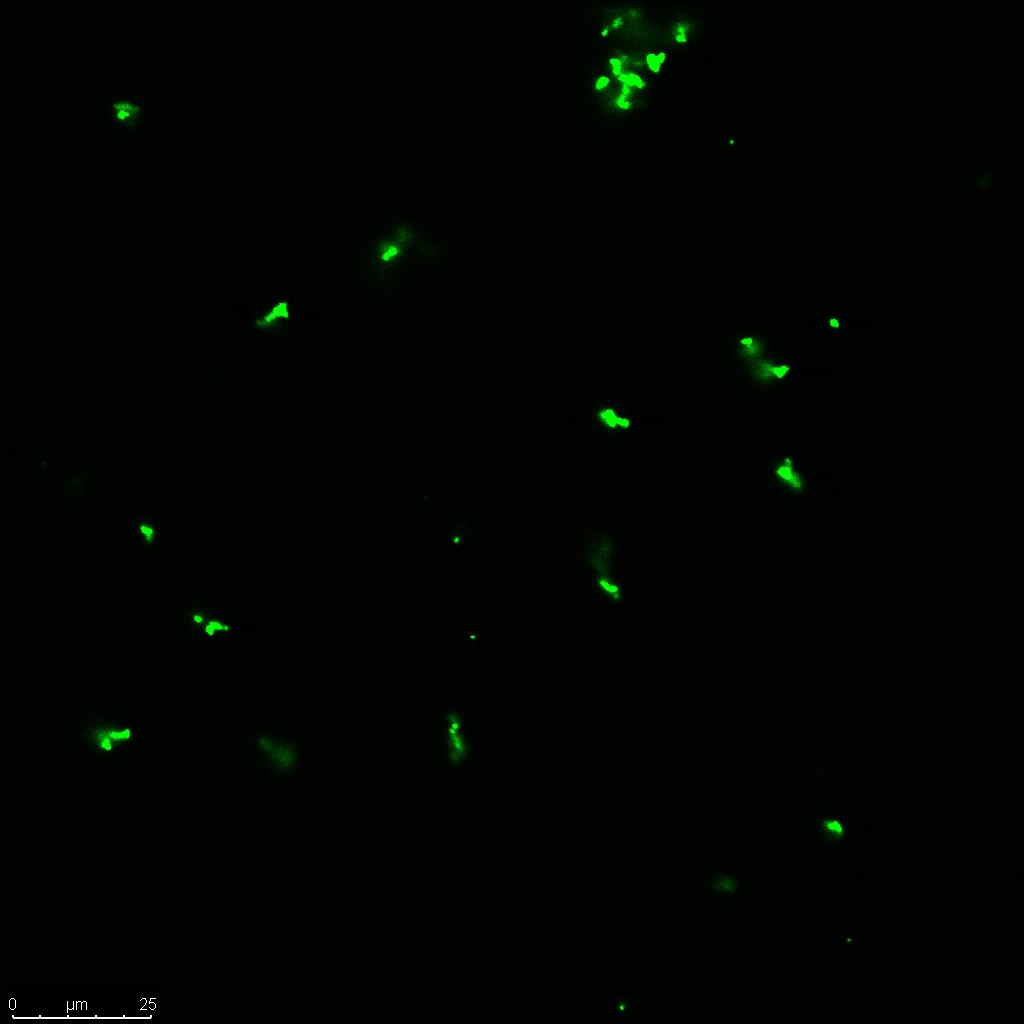

Supplement: Figure 3—figure supplement 1—source data 3. [file elife-94795-fig3-figsupp1-data3.zip › Figure 3—figure supplement 1B/replicate I/Figure 3—figure supplement 1B replicate 1 photo 6.jpg]

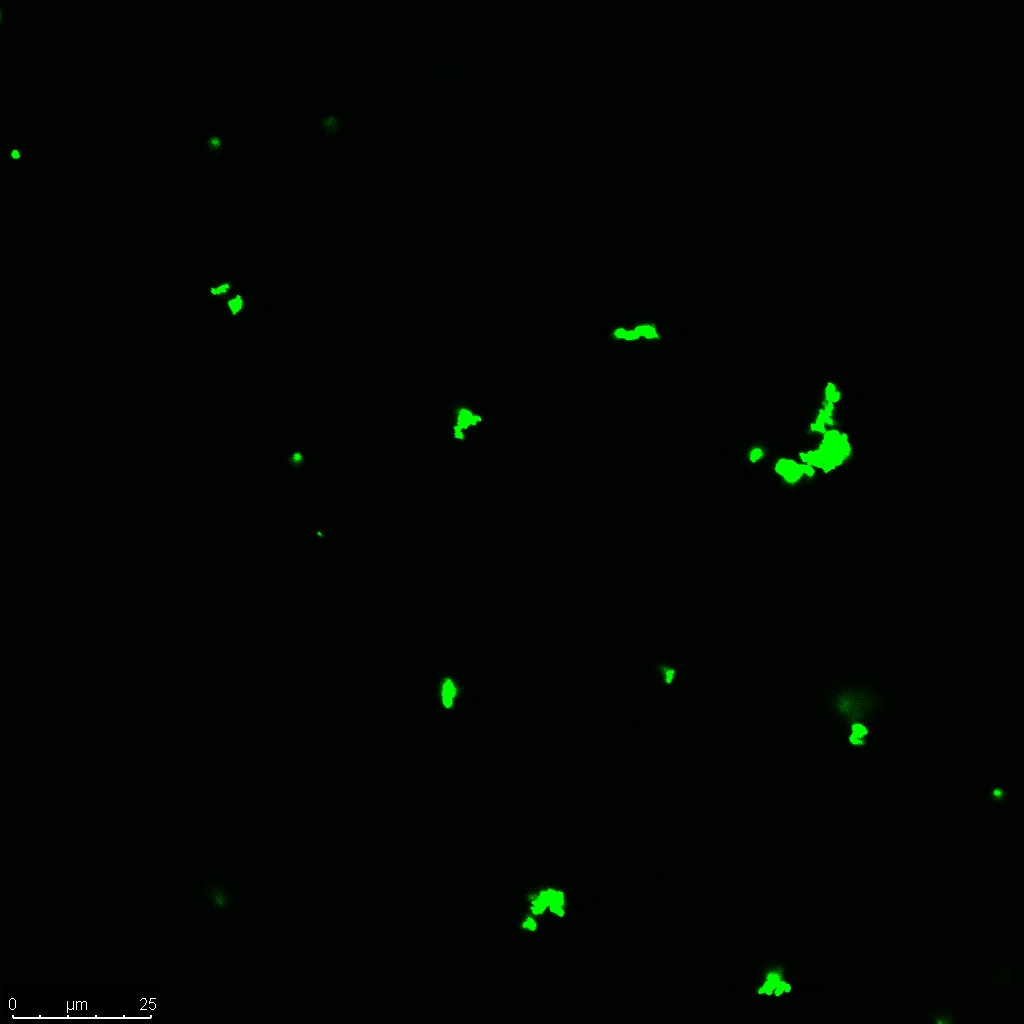

Supplement: Figure 3—figure supplement 1—source data 3. [file elife-94795-fig3-figsupp1-data3.zip › Figure 3—figure supplement 1B/replicate I/Figure 3—figure supplement 1B replicate 1 photo 7.jpg]

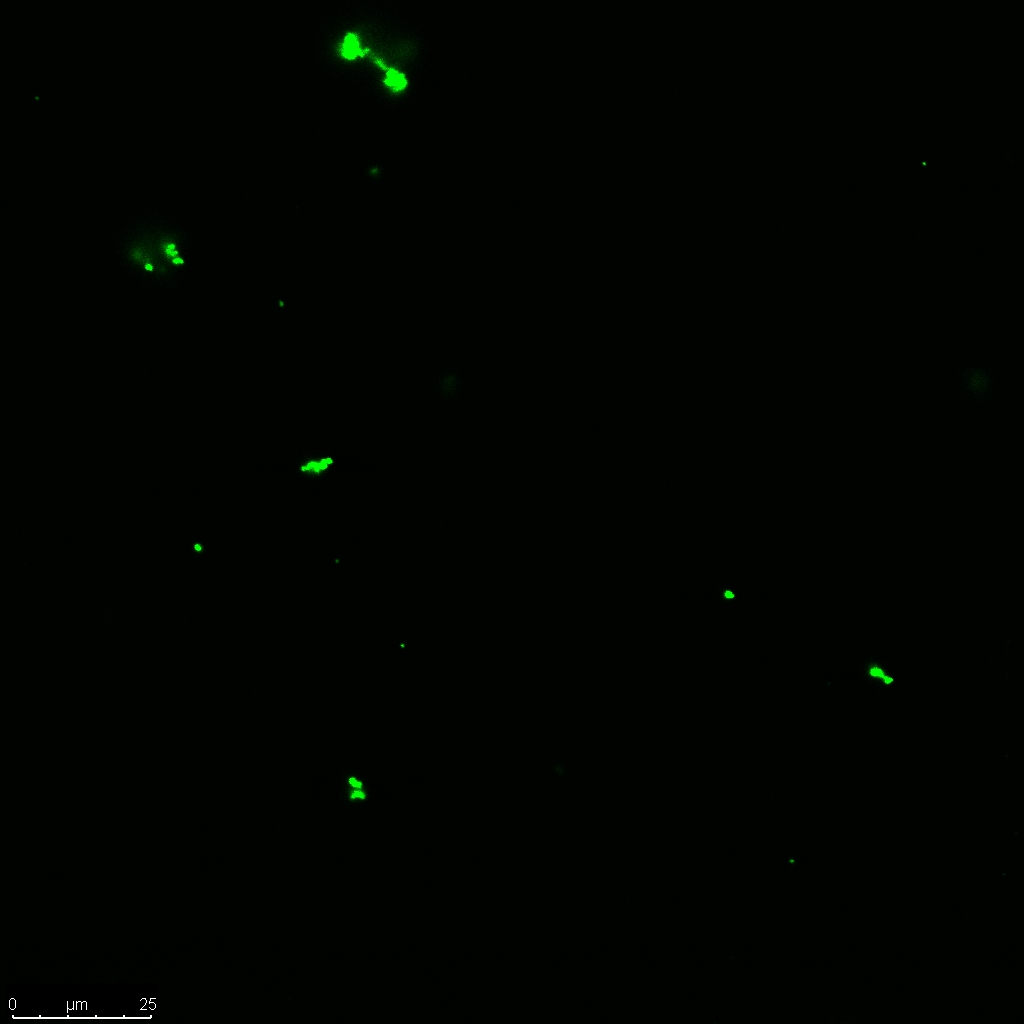

Supplement: Figure 3—figure supplement 1—source data 3. [file elife-94795-fig3-figsupp1-data3.zip › Figure 3—figure supplement 1B/replicate I/Figure 3—figure supplement 1B replicate 1 photo 8.jpg]

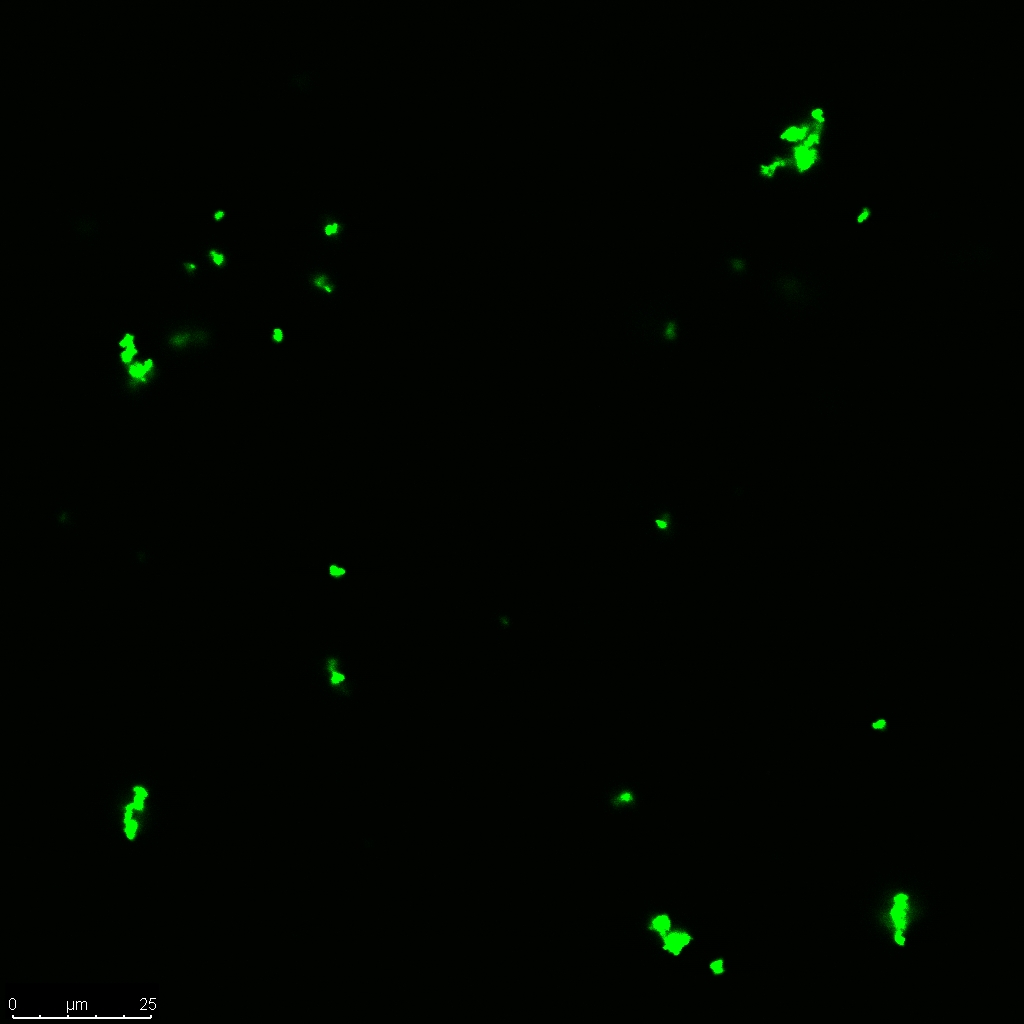

Supplement: Figure 3—figure supplement 1—source data 3. [file elife-94795-fig3-figsupp1-data3.zip › Figure 3—figure supplement 1B/replicate I/Figure 3—figure supplement 1B replicate 1 photo 9.jpg]

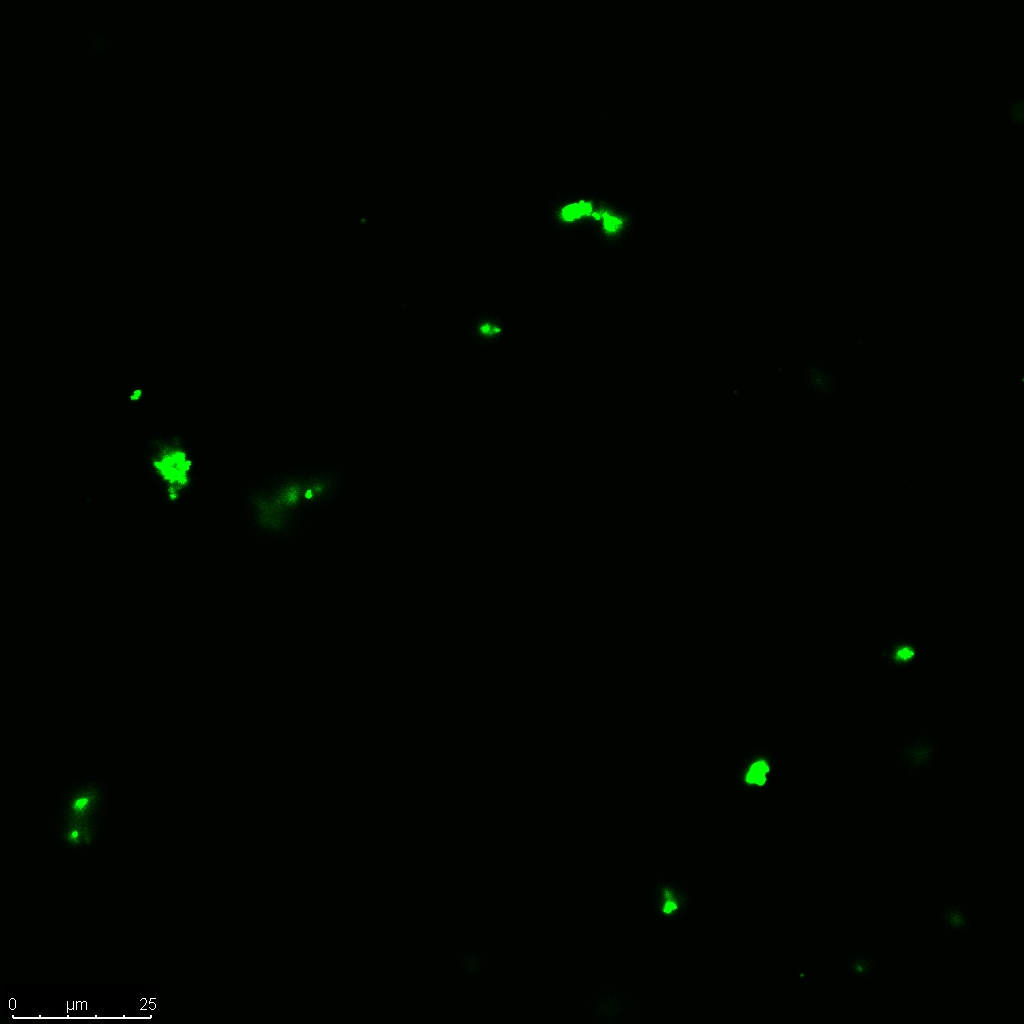

Supplement: Figure 3—figure supplement 1—source data 3. [file elife-94795-fig3-figsupp1-data3.zip › Figure 3—figure supplement 1B/replicate I/Figure 3—figure supplement 1B replicate 1 photo 10.jpg]

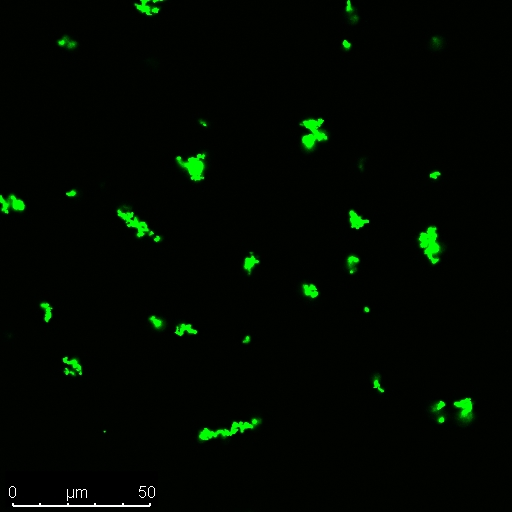

Supplement: Figure 3—figure supplement 1—source data 3. [file elife-94795-fig3-figsupp1-data3.zip › Figure 3—figure supplement 1B/replicate II/Figure 3—figure supplement 1B replicate 2 photo 1.jpg]

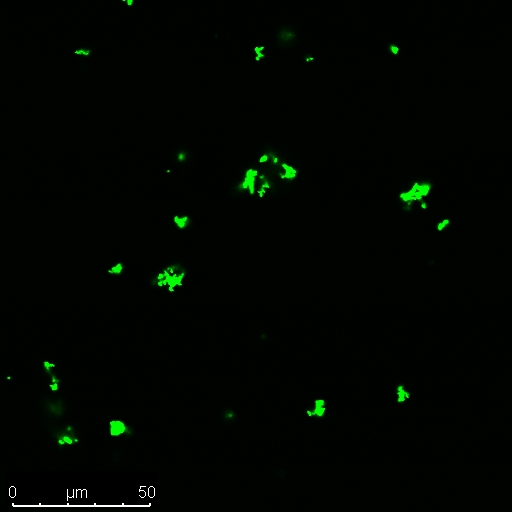

Supplement: Figure 3—figure supplement 1—source data 3. [file elife-94795-fig3-figsupp1-data3.zip › Figure 3—figure supplement 1B/replicate II/Figure 3—figure supplement 1B replicate 2 photo 2.jpg]

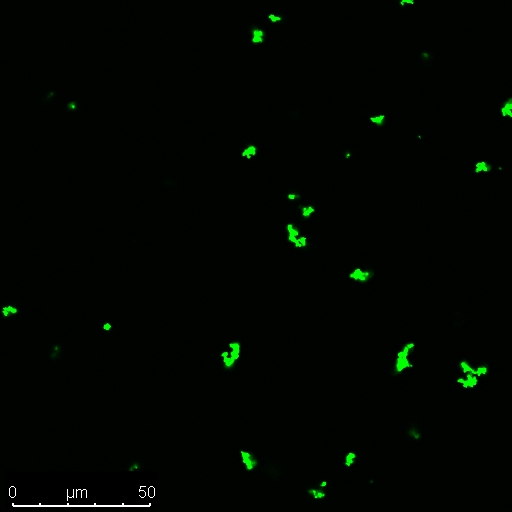

Supplement: Figure 3—figure supplement 1—source data 3. [file elife-94795-fig3-figsupp1-data3.zip › Figure 3—figure supplement 1B/replicate II/Figure 3—figure supplement 1B replicate 2 photo 3.jpg]
