# Supplementary figures and images for: Early steps of protein disaggregation by Hsp70 chaperone and class B J-domain proteins are shaped by Hsp110 (part 3 of 3)
Source: eLife. 2024 Oct 15;13:RP94795. doi: 10.7554/eLife.94795 (PMC11479587; doi:10.7554/eLife.94795)

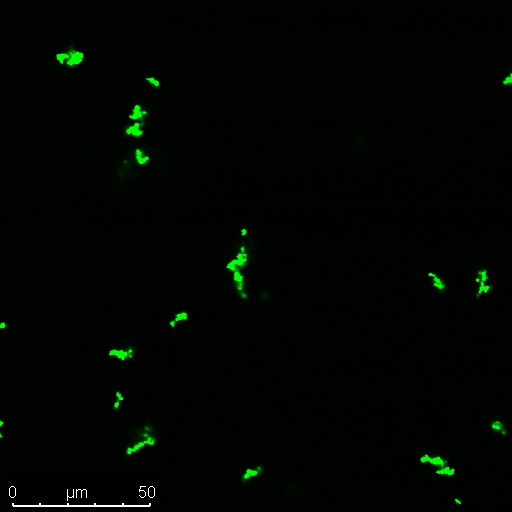

Supplement: Figure 3—figure supplement 1—source data 3. [file elife-94795-fig3-figsupp1-data3.zip › Figure 3—figure supplement 1B/replicate II/Figure 3—figure supplement 1B replicate 2 photo 4.jpg]

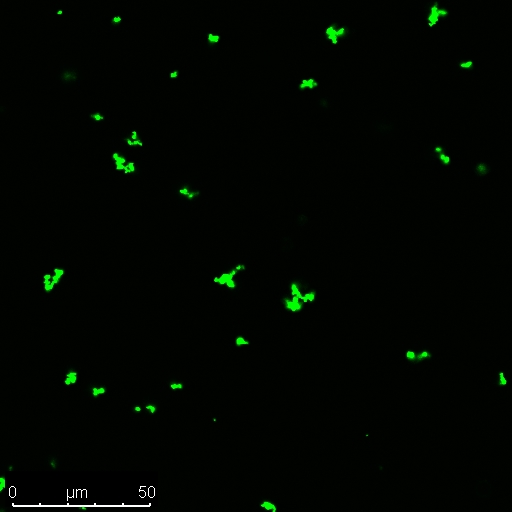

Supplement: Figure 3—figure supplement 1—source data 3. [file elife-94795-fig3-figsupp1-data3.zip › Figure 3—figure supplement 1B/replicate II/Figure 3—figure supplement 1B replicate 2 photo 5.jpg]

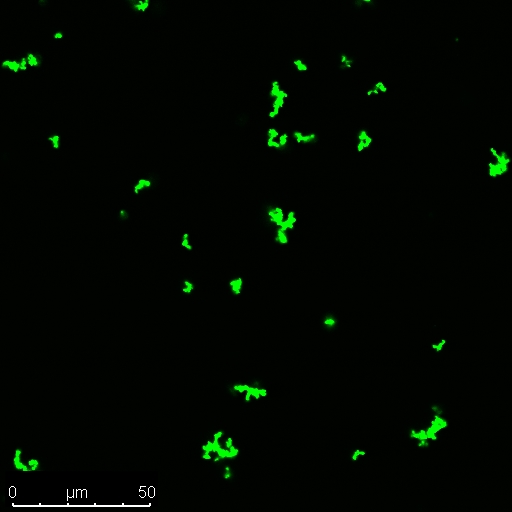

Supplement: Figure 3—figure supplement 1—source data 3. [file elife-94795-fig3-figsupp1-data3.zip › Figure 3—figure supplement 1B/replicate II/Figure 3—figure supplement 1B replicate 2 photo 6.jpg]

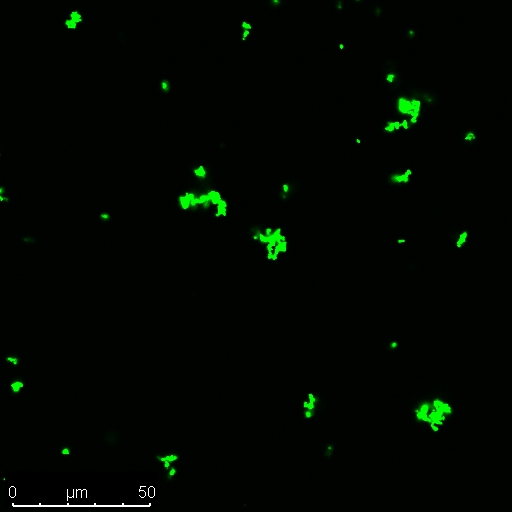

Supplement: Figure 3—figure supplement 1—source data 3. [file elife-94795-fig3-figsupp1-data3.zip › Figure 3—figure supplement 1B/replicate II/Figure 3—figure supplement 1B replicate 2 photo 7.jpg]

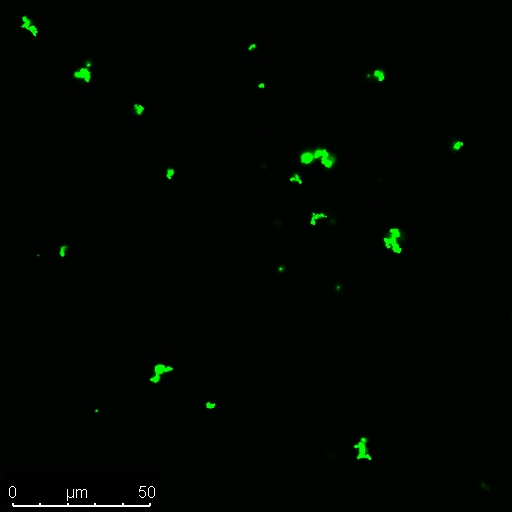

Supplement: Figure 3—figure supplement 1—source data 3. [file elife-94795-fig3-figsupp1-data3.zip › Figure 3—figure supplement 1B/replicate II/Figure 3—figure supplement 1B replicate 2 photo 8.jpg]

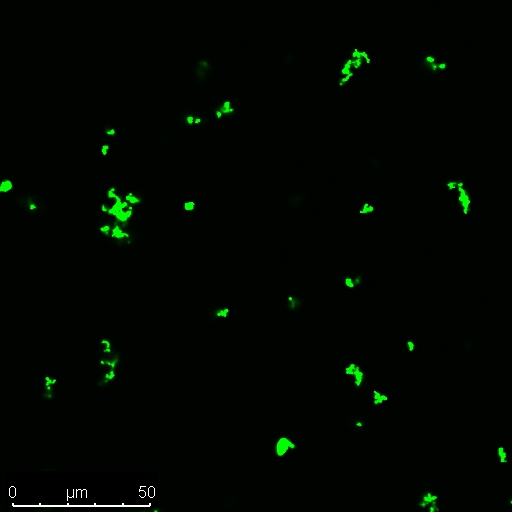

Supplement: Figure 3—figure supplement 1—source data 3. [file elife-94795-fig3-figsupp1-data3.zip › Figure 3—figure supplement 1B/replicate II/Figure 3—figure supplement 1B replicate 2 photo 9.jpg]

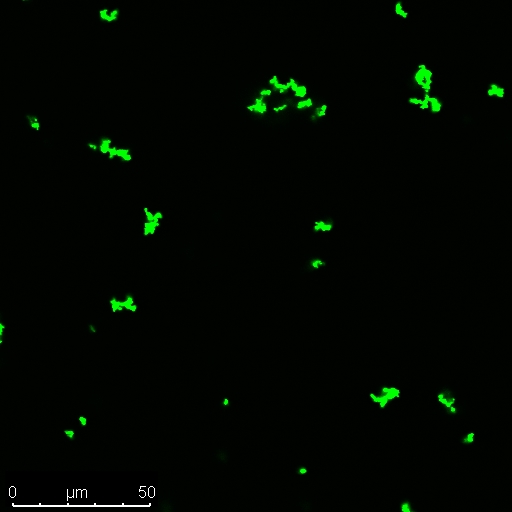

Supplement: Figure 3—figure supplement 1—source data 3. [file elife-94795-fig3-figsupp1-data3.zip › Figure 3—figure supplement 1B/replicate II/Figure 3—figure supplement 1B replicate 2 photo 10.jpg]

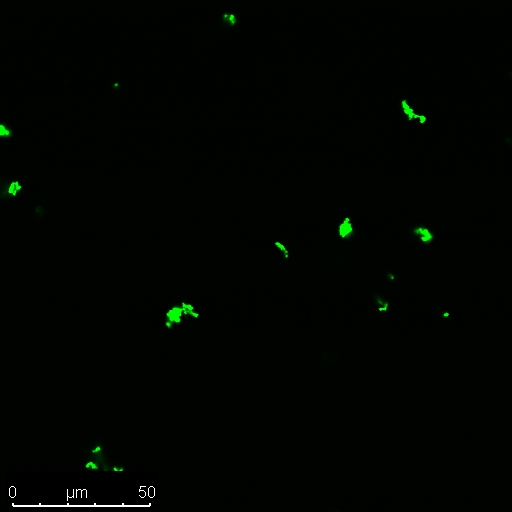

Supplement: Figure 3—figure supplement 1—source data 3. [file elife-94795-fig3-figsupp1-data3.zip › Figure 3—figure supplement 1B/replicate III/2024.04.09 powtorzenia recenzja.lif_Luc agg - SS 1uM Sse-powtorzenie3-001_ch00.jpg]

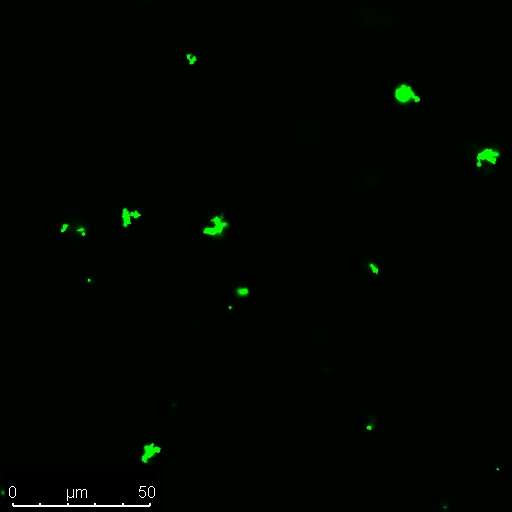

Supplement: Figure 3—figure supplement 1—source data 3. [file elife-94795-fig3-figsupp1-data3.zip › Figure 3—figure supplement 1B/replicate III/2024.04.09 powtorzenia recenzja.lif_Luc agg - SS 1uM Sse-powtorzenie3-002_ch00.jpg]

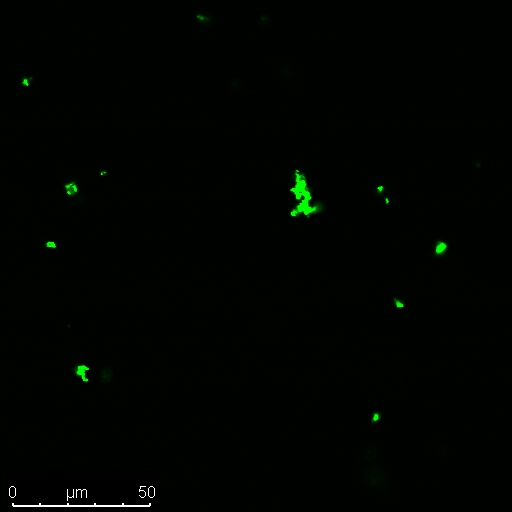

Supplement: Figure 3—figure supplement 1—source data 3. [file elife-94795-fig3-figsupp1-data3.zip › Figure 3—figure supplement 1B/replicate III/2024.04.09 powtorzenia recenzja.lif_Luc agg - SS 1uM Sse-powtorzenie3-003_ch00.jpg]

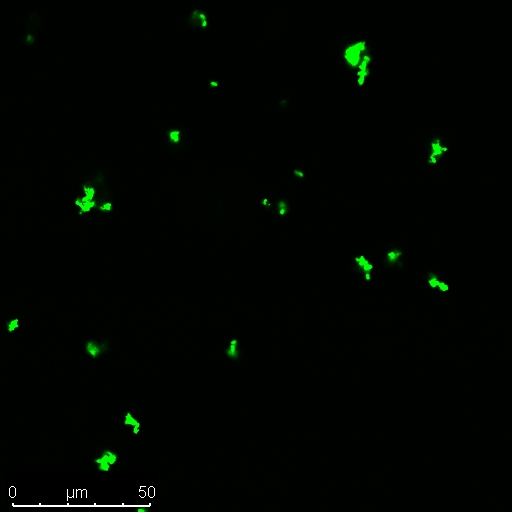

Supplement: Figure 3—figure supplement 1—source data 3. [file elife-94795-fig3-figsupp1-data3.zip › Figure 3—figure supplement 1B/replicate III/2024.04.09 powtorzenia recenzja.lif_Luc agg - SS 1uM Sse-powtorzenie3-004_ch00.jpg]

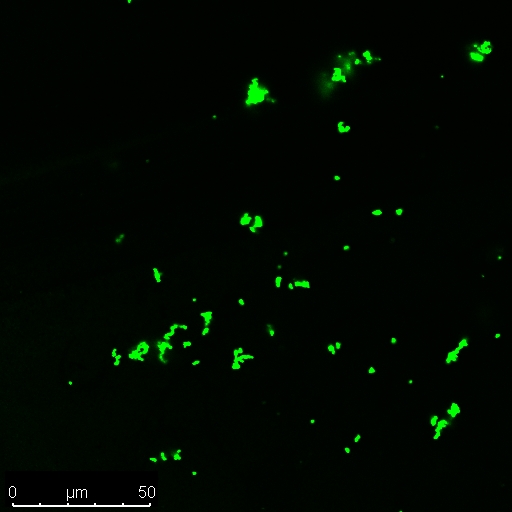

Supplement: Figure 3—figure supplement 1—source data 3. [file elife-94795-fig3-figsupp1-data3.zip › Figure 3—figure supplement 1B/replicate III/2024.04.09 powtorzenia recenzja.lif_Luc agg - SS 1uM Sse-powtorzenie3-005_ch00.jpg]

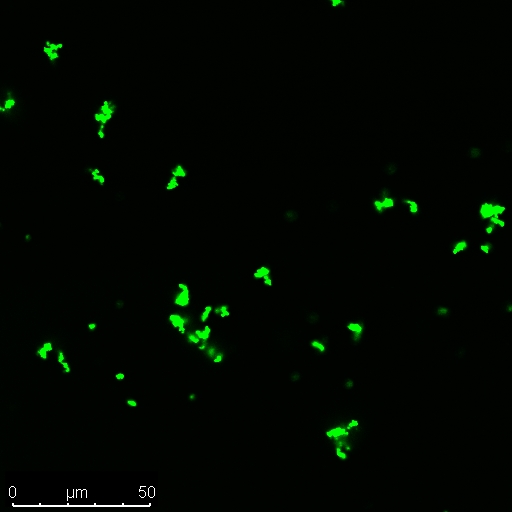

Supplement: Figure 3—figure supplement 1—source data 3. [file elife-94795-fig3-figsupp1-data3.zip › Figure 3—figure supplement 1B/replicate III/2024.04.09 powtorzenia recenzja.lif_Luc agg - SS 1uM Sse-powtorzenie3-006_ch00.jpg]

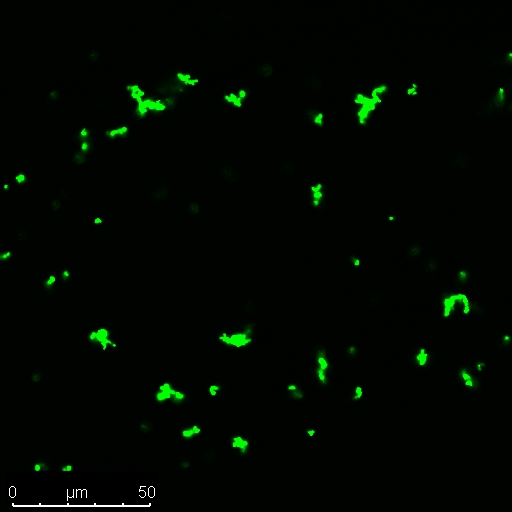

Supplement: Figure 3—figure supplement 1—source data 3. [file elife-94795-fig3-figsupp1-data3.zip › Figure 3—figure supplement 1B/replicate III/2024.04.09 powtorzenia recenzja.lif_Luc agg - SS 1uM Sse-powtorzenie3-007_ch00.jpg]

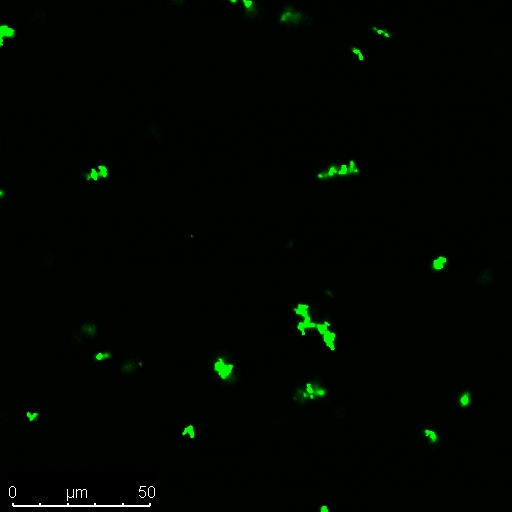

Supplement: Figure 3—figure supplement 1—source data 3. [file elife-94795-fig3-figsupp1-data3.zip › Figure 3—figure supplement 1B/replicate III/2024.04.09 powtorzenia recenzja.lif_Luc agg - SS 1uM Sse-powtorzenie3-008_ch00.jpg]

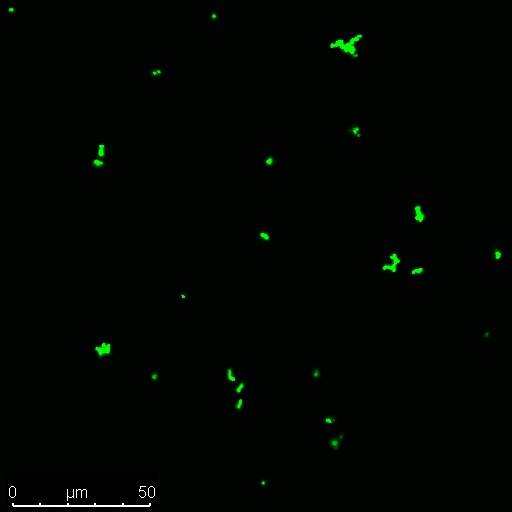

Supplement: Figure 3—figure supplement 1—source data 3. [file elife-94795-fig3-figsupp1-data3.zip › Figure 3—figure supplement 1B/replicate III/2024.04.09 powtorzenia recenzja.lif_Luc agg - SS 1uM Sse-powtorzenie3-009_ch00.jpg]

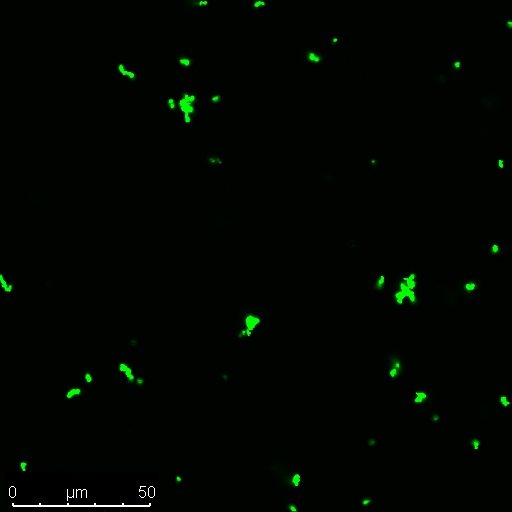

Supplement: Figure 3—figure supplement 1—source data 3. [file elife-94795-fig3-figsupp1-data3.zip › Figure 3—figure supplement 1B/replicate III/2024.04.09 powtorzenia recenzja.lif_Luc agg - SS 1uM Sse-powtorzenie3-010_ch00.jpg]
